# One-pot synthesis of oxazolidinones and five-membered cyclic carbonates from epoxides and chlorosulfonyl isocyanate: theoretical evidence for an asynchronous concerted pathway

**DOI:** 10.3762/bjoc.16.148

**Published:** 2020-07-21

**Authors:** Esra Demir, Ozlem Sari, Yasin Çetinkaya, Ufuk Atmaca, Safiye Sağ Erdem, Murat Çelik

**Affiliations:** 1 Department of Chemistry, Faculty of Science, Atatürk University, 25240 Erzurum, Turkey; 2 Department of Chemistry, Faculty of Arts and Sciences, Kırşehir Ahi Evran University, 40100 Kırşehir, Turkey; 3 Department of Food Technology, Oltu Vocational School, Atatürk University, 25400 Oltu, Erzurum, Turkey; 4 Department of Chemistry, Faculty of Arts and Sciences, Marmara University, Goztepe Campus, 34722 Istanbul, Turkey

**Keywords:** chlorosulfonyl isocyanate, computational modeling, cyclic carbonates, density functional theory, oxazolidinone

## Abstract

The one-pot reaction of chlorosulfonyl isocyanate (CSI) with epoxides having phenyl, benzyl and fused cyclic alkyl groups in different solvents under mild reaction conditions without additives and catalysts was studied. Oxazolidinones and five-membered cyclic carbonates were obtained in ratios close to 1:1 in the cyclization reactions. The best yields of these compounds were obtained in dichloromethane (DCM). Together with 16 known compounds, two novel oxazolidinone derivatives and two novel cyclic carbonates were synthesized with an efficient and straightforward method. Compared to the existing methods, the synthetic approach presented here provides the following distinct advantageous: being a one-pot reaction with metal-free reagent, having shorter reaction times, good yields and a very simple purification method. Moreover, using the density functional theory (DFT) method at the M06-2X/6-31+G(d,p) level of theory the mechanism of the cycloaddition reactions has been elucidated. The further investigation of the potential energy surfaces associated with two possible channels leading to oxazolidinones and five-membered cyclic carbonates disclosed that the cycloaddition reaction proceeds via an asynchronous concerted mechanism in gas phase and in DCM.

## Introduction

**Scheme 1:**
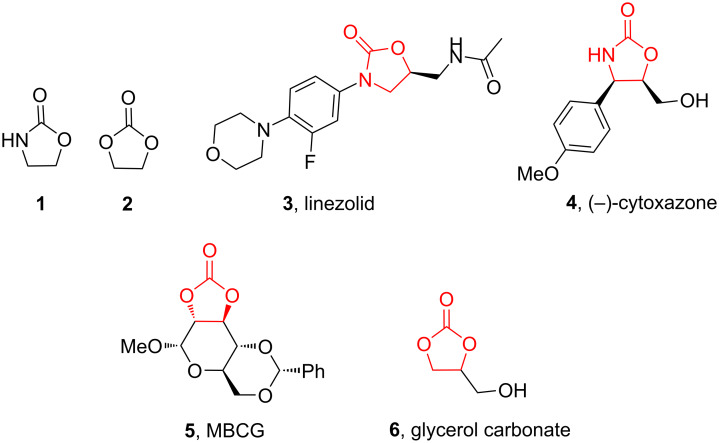
Oxazolidinone (**1**), five-membered cyclic carbonate (**2**) and some important compounds containing an oxazolidinone ring (**3**, **4**) or a five-membered cyclic carbonate (**5**, **6**).

Oxazolidinones (**1**), five-membered heterocyclic rings containing an ester group adjacent to a nitrogen atom, are important compounds in synthetic and pharmaceutical chemistry because of their considerable use as antibiotics [1], immunomodulators [2], antibacterials [3], as well as synthetic intermediates and chiral auxiliaries for various organic conversions [4–7]. Linezolid [1–3] (**3**) and cytoxazone [8–9] (**4**) are oxazolidinone derivatives having significant biological activities. Linezolid (**3**) is the first oxazolidinone drug approved in 2000 by the Food and Drug Administration (FDA) for the treatment of multidrug resistant Gram-positive bacterial infections (Scheme 1) [10]. Cytoxazone is a microbial metabolite exhibiting potent cytokine-modulating activity. Tedizolid phosphate (trade name Sivextro), which exhibits antibiotic activity is another oxazolidinone drug approved by the FDA in 2014 [11]. Befloxatone and toloxatone, *N*-substituted phenyloxazolidinone derivatives, are reversible inhibitors of monoamine oxidase (MAO) [12–13]. *N*-Aryloxazolidinedione compounds, which are toloxatone derivatives, have been reported to exhibit good affinity for human MAO-A [14].

Five-membered cyclic carbonate (1,3-dioxolan-2-one) (**2**) and its derivatives are valuable synthetic targets on account of several applications and pertinent properties. They are found in various natural and potential pharmaceutical products [15]. Moreover, they are used as electrolyte components in Li-ion rechargeable cells and as aprotic polar solvent with high boiling point as alternative of dangerous solvents because of their good biodegradability and low toxicity [16–18]. Synthetic intermediates for ring-opening polymerization of the compounds containing cyclic carbonates such as methyl 4,6-*O*-benzylidene-2,3-*O*-carbonyl-α-ᴅ-glucopyranoside (MBCG) (**5**) [19–20] and glycerol carbonate (**6)** [21] were also reported (Scheme 1).

Therefore, numerous synthetic approaches have been developed to date for the preparation of oxazolidinones and five-membered cyclic carbonates of various structures. The most well-known strategies for the synthesis of oxazolidinones are the reaction of an amino alcohol with phosgene [5,22], the carbonylation reaction of β-amino alcohols with CO_2_ or dialkyl carbonates [23–27], the multicomponent reaction of rare-earth metal amides [28], the reaction of CO_2_ with propargylamines or aziridines [29–30] and the cycloaddition reaction of epoxides with isocyanates [31–32]. On the other hand, for the synthesis of five-membered cyclic carbonates, the cycloaddition of CO_2_ to epoxides, the reaction with the metal complexes or catalysts, and the reaction of a diol with toxic phosgene are the most common processes [16–17,33–36].

CSI, a highly reactive and versatile isocyanate, reacts with epoxides to give five-membered cyclic carbonates and oxazolidinones [37–39]. In 1984, Keshava Murthy and Dhar reported the synthesis of five-membered cyclic carbonates and oxazolidinones from various epoxides in two steps using CSI and KOH in benzene/dichloromethane [40–41]. In 1986, De Meijere and co-workers reported the cycloaddition of CSI to epoxides at −78 °C to give five-membered cyclic carbonates and oxazolidinones [42]. They reported seven examples; three of these attempts resulted in five-membered cyclic carbonates as the sole product while two cases produced oxazolidinones, and the other two reactions gave mixtures of two products. These reports prompted us to explore this reaction in more detail. In our previous studies, we investigated the reactions of CSI with various substrates such as carboxylic acids, alkenes and allyl or benzyl alcohols [43–46]. As a continuation of these studies, we performed one-pot syntheses of the title compounds by optimizing the reaction of CSI with epoxides in different solvents under mild conditions and compared the reaction mechanism with previously proposed mechanisms using theoretical calculations.

**Scheme 2:**
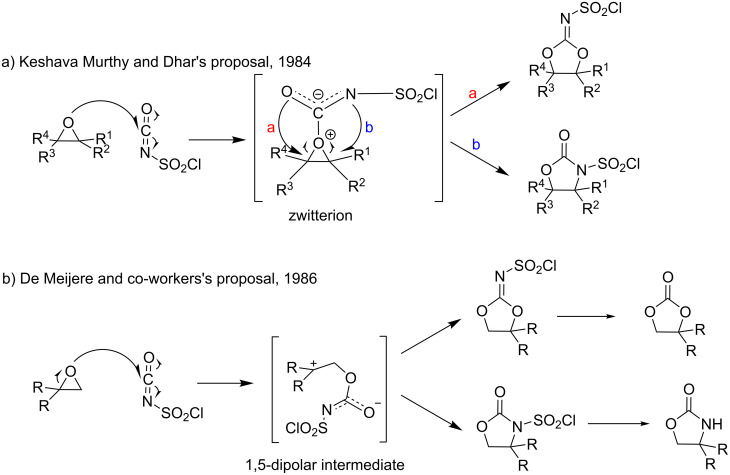
Proposed mechanisms by Keshava Murthy and Dhar [41] and De Meijere and co-workers [42].

Keshava Murthy and Dhar [41] postulated a mechanism involving a zwitterionic intermediate. C–O bond cleavage in this unstable and strained intermediate gives rise to a short-lived carbonium ion which will be attacked by the nucleophilic part of the zwitterion in a concerted way (Scheme 2a). De Meijere and co-workers [42] proposed a mechanism involving a 1,5-dipolar intermediate (Scheme 2b).

To the best of our knowledge, there is no computational mechanistic study in the literature regarding the reaction of epoxides with CSI. On the other hand, the reactions of isocyanates with monofluoroalkenes and nitrones were modeled with the Møller–Plesset (MP2) perturbation theory and M06-2X functional, respectively [37,47]. According to these computational studies, such reactions of isocyanates may proceed through a concerted pathway. The remaining uncertainties in the mechanisms of the similar reactions inspired us to carry out quantum chemical calculations for the formation of oxazolidinone and five-membered cyclic carbonates.

## Results and Discussion

First, we synthesized various epoxides (**7a–j**) in the presence of *meta*-chloroperbenzoic acid (*m*-CPBA), from the corresponding alkenes dissolved in DCM at room temperature. The general experimental conditions for conversion of alkenes to related epoxides were given in Supporting Information File 1. For the synthesis of oxazolidinones and five-membered cyclic carbonates, the most effective solvent was determined based on the reaction of 8-oxabicyclo[5.1.0]octane (**7b**) with CSI (Table 1) which was the first reaction performed in this study. The reaction was carried out in acetone, THF, acetonitrile, dichloromethane, toluene, and *n*-hexane/dichloromethane. While no reaction was observed in diethyl ether, the best conversion was achieved in dichloromethane. Benzene was not used as a solvent because of having toxic and carcinogenic effects.

**Table 1:** Solvent optimization for the synthesis of five-membered cyclic carbonate **8b** and oxazolidinone **9b** from epoxide **7b**.

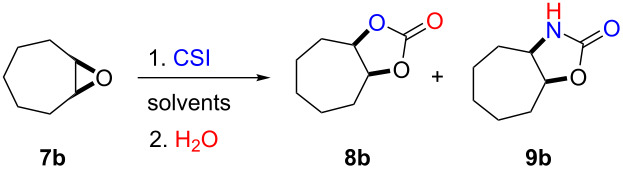			

Entry	Solvents	Products (%)^a^	

		Five-membered cyclic carbonate **8b**	Oxazolidinone **9b**

1	acetone	11	15
2	THF	15	12
3	diethyl ether	no reaction	
4	CH_3_CN	39	34
**5**	**dichloromethane**	**48**	**45**
6	toluene	13	15
7	*n*-hexane/dichloromethane (1:1)	21	19

^a^Isolated yield.

Herein, we report mild reaction conditions for the one-pot synthesis of oxazolidinones and five-membered cyclic carbonates from various epoxides (**7a–j**) at room temperature without using any catalyst.

After having identified the optimal conditions, various epoxides were treated with chlorosulfonyl isocyanate at room temperature to give mixtures of cyclic carbonates and oxazolidinones in ratios close to 1:1 as shown in Table 2. *trans*-Stilbene (**7d**) and *cis***-**stilbene epoxids (**7e**) in the presence of CSI gave *trans*-4,5-diphenyl-1,3-dioxolan-2-one (**8d**), *trans*-4,5-diphenyloxazolidin-2-one (**9d**) and *cis*-4,5-diphenyl-1,3-dioxolan-2-one (**8e**), *cis*-4,5-diphenyloxazolidin-2-one (**9e**), respectively (Table 2). These results show that the relative configuration is preserved. 4*-*Phenyl-1,3-dioxolan-2-one (**8f**) and 4-phenyloxazolidin-2-one (**9f**) were obtained from the reaction of CSI with styrene oxide (**7f**) showing the regioselective nature of the reaction. In addition, we report the synthesis of the novel oxazolidinone derivatives **8i**, and **8j** and 1,3-dioxolan-2-ones **9c** and **9i.** Furthermore, a more efficient and straightforward method for the formation of the previously known **8a–h**, **9a–b**, **9d–h** and **9j** is being described.

**Table 2:** Direct conversion of epoxides **7a–j** with CSI into five-membered cyclic carbonates **8a–j** and oxazolidinones **9a–j**.

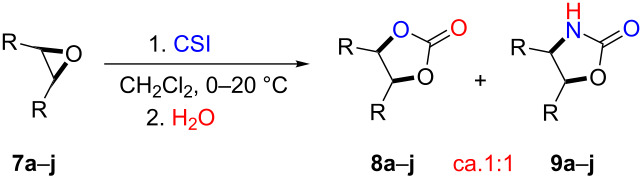			

Entry	Substrates^a^	Products^b^ (%)	

		Five-membered cyclic carbonates^c^	Oxazolidinones^c^

1	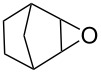 **7a**	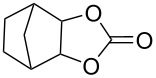 42% **8a** [48]	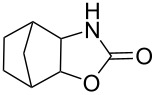 38% **9a** [49]
2	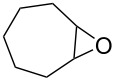 **7b**	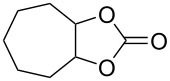 48% **8b** [50]	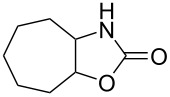 45% **9b** [51]
3	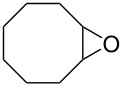 **7c**	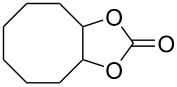 51% **8c** [50]	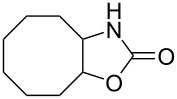 40% **9c**
4	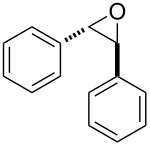 **7d**	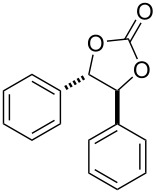 43% **8d** [50]	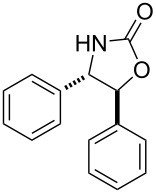 34% **9d** [52]
5	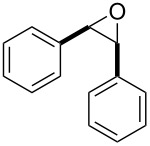 **7e**	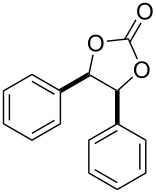 44% **8e** [50]	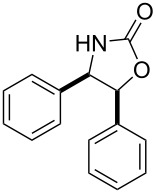 41% **9e** [52]
6	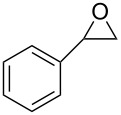 **7f**	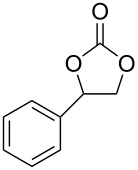 49% **8f** [16]	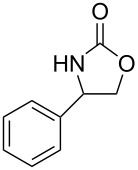 42% **9f** [16]
7	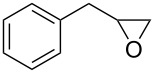 **7g**	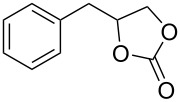 41% **8g** [16]	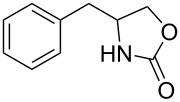 35% **9g** [53]
8	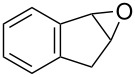 **7h**	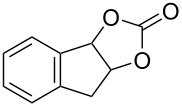 45% **8h** [54]	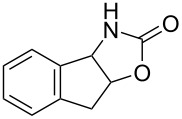 40% **9h** [52]
9	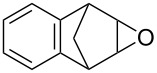 **7i**	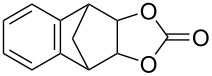 43% **8i**	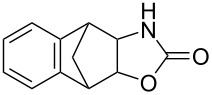 37% **9i**
10	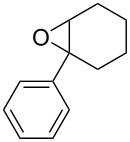 **7j**	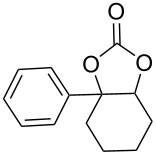 44% **8j**	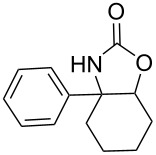 42% **9j** [55]

^a^Synthesis of epoxides **7a–j**: alkenes (1 equiv), *m*-CPBA (1.2 equiv), in DCM; ^b^isolated yield; ^c^literature.

In the study of Keshava Murthy and Dhar, five-membered cyclic carbonates and oxazolidinones from epoxides were synthesized in two stages using CSI and KOH in dry benzene/dichloromethane (5:1) at −10 °C [40–41]. They reported five-membered cyclic carbonates as the main products in the reaction mixture in good yields (83.9–95.9%, totally), but with only five examples. It is also known today that benzene is carcinogenic, and not preferred as a solvent unless it is necessary. This two-step methodology required several purification methods. However, our purification process is remarkably simple and shorter. Moreover, using ten examples (Table 2), twenty distinct products were synthesized in good yields (76–93%, totally).

On the other hand, in the study of De Meijere and co-workers, the reaction started at −78 °C to give five-membered cyclic carbonates and oxazolidinones using seven examples [42]. Three of these attempts resulted in five-membered cyclic carbonates as the sole products while in two cases oxazolidinones were produced, and the other two reactions gave mixtures of two products. The purification process of this method also required several steps resulting in relatively lower yields (20–67% yields, totally). Compared to this study, our study provided higher yields in shorter reaction times under mild conditions using a simple purification method. Apparently, our protocol describes a reasonable methodology for the conversion of epoxides to protected 1,2-diols and 2-amino alcohols. Attention is drawn on these 1,2-oxygen and/or nitrogen units since they are present in natural products ranging from small molecules, such as sugars, lipids and amino acids to huge molecules [56].

### Computational results

A detailed mechanistic investigation of the synthesis of oxazolidinone and five-membered cyclic carbonate derivatives by the reaction between epoxide **7f** and CSI has been performed.

#### Formation of oxazolidinone **9f**

**Figure 1:**
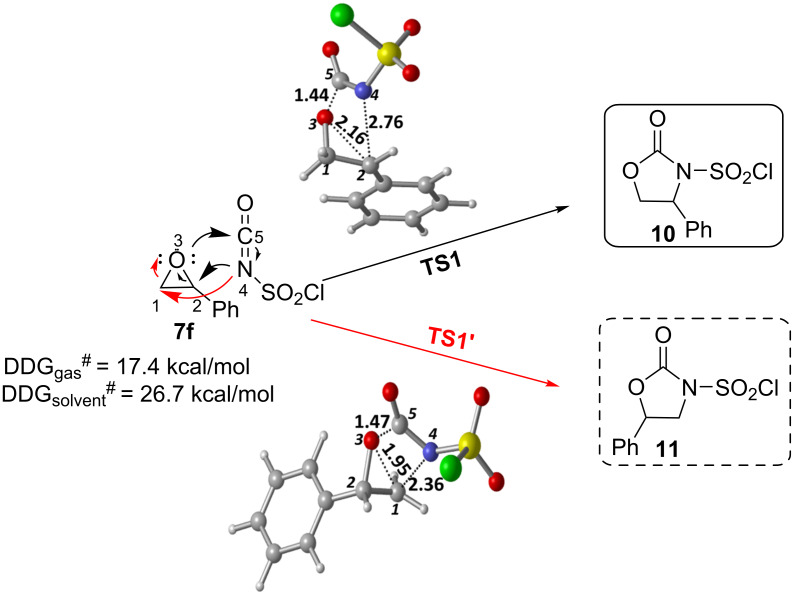
Possible pathways for the formation of oxazolidinone intermediates **10** and **11**. Optimized transition structures at PCM/M06-2X/6-31+G(d,p)//M06-2X/6-31+G(d,p) level in DCM. Distances are given in Å.

There are two possible channels for the cyclization reaction of epoxide **7f** with CSI to form oxazolidinone intermediates **10** and **11** as shown in Figure 1. In both transition states it is found that the ring-opening reaction of the epoxide, a nucleophilic attack of N4 onto C1 or C2 and an attack of O3 on C5 occur in an asynchronous concerted manner. The first transition state (**TS1**) corresponds to the nucleophilic attack of N4 onto the C2 of **7f** leading to oxazolidinone intermediate **10**. The alternative transition state (**TS1′**) corresponds to the nucleophilic attack of N4 onto the less sterically encumbered C1 atom of the epoxide **7f** forming intermediate **11**. Optimized geometries of transition structures are depicted in Figure 1.

**Figure 2:**
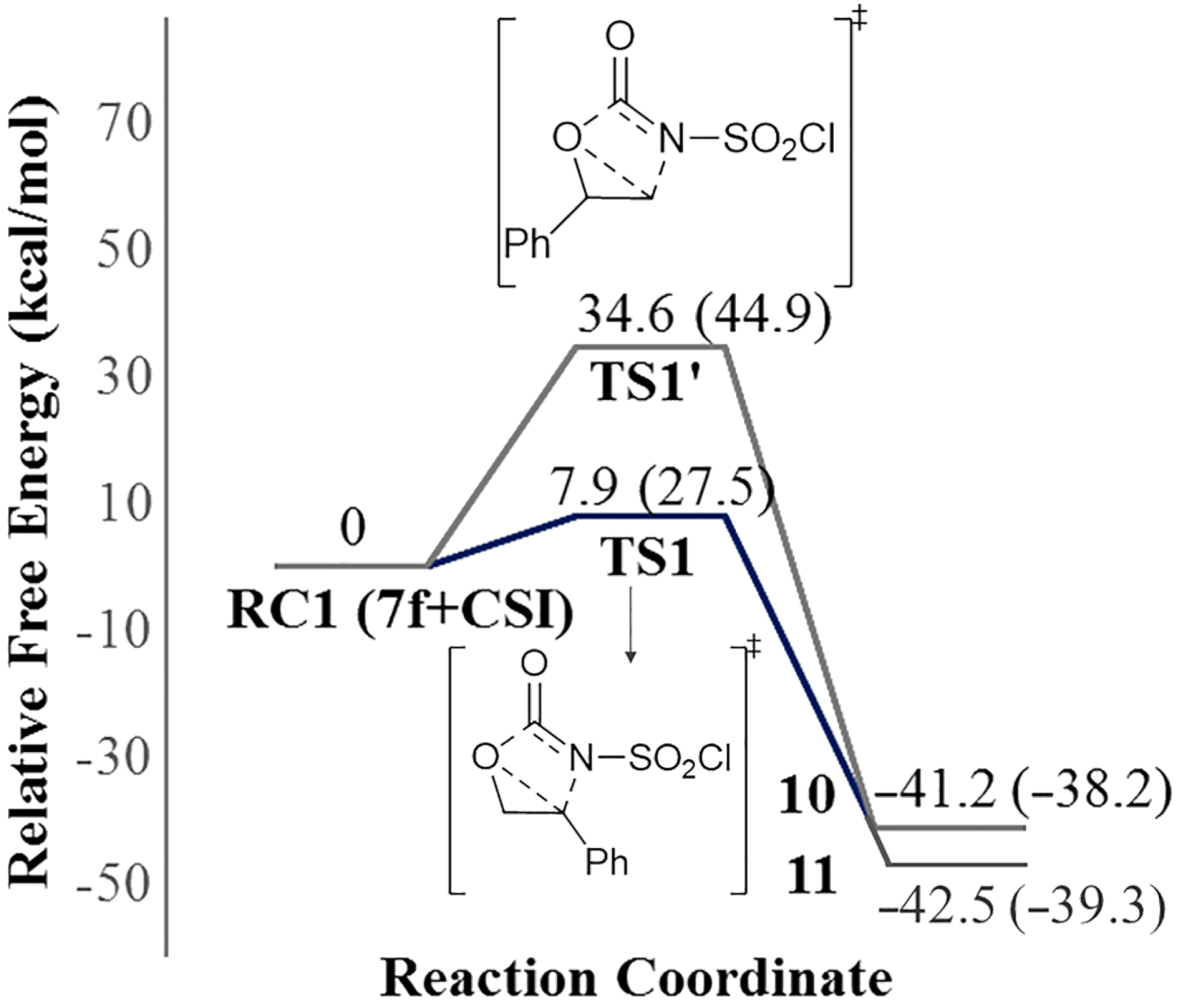
Potential energy profile related to the formation of oxazolidinone intermediates **10** and **11** at the PCM/M06-2X/6-31+G(d,p)//M06-2X/6-31+G(d,p) level in DCM. Gas phase energies are shown in parenthesis. (The polarization effect of the solvent was considered implicitly.)

Our calculated results for the reaction indicate 17.4 kcal/mol (gas phase) and 26.7 kcal/mol (in DCM) preference for the **TS1** over the **TS1′** (Figure 2). Therefore, attack by N4 of CSI on the C2 of epoxide is found to be energetically the most favored approach.

**Figure 3:**
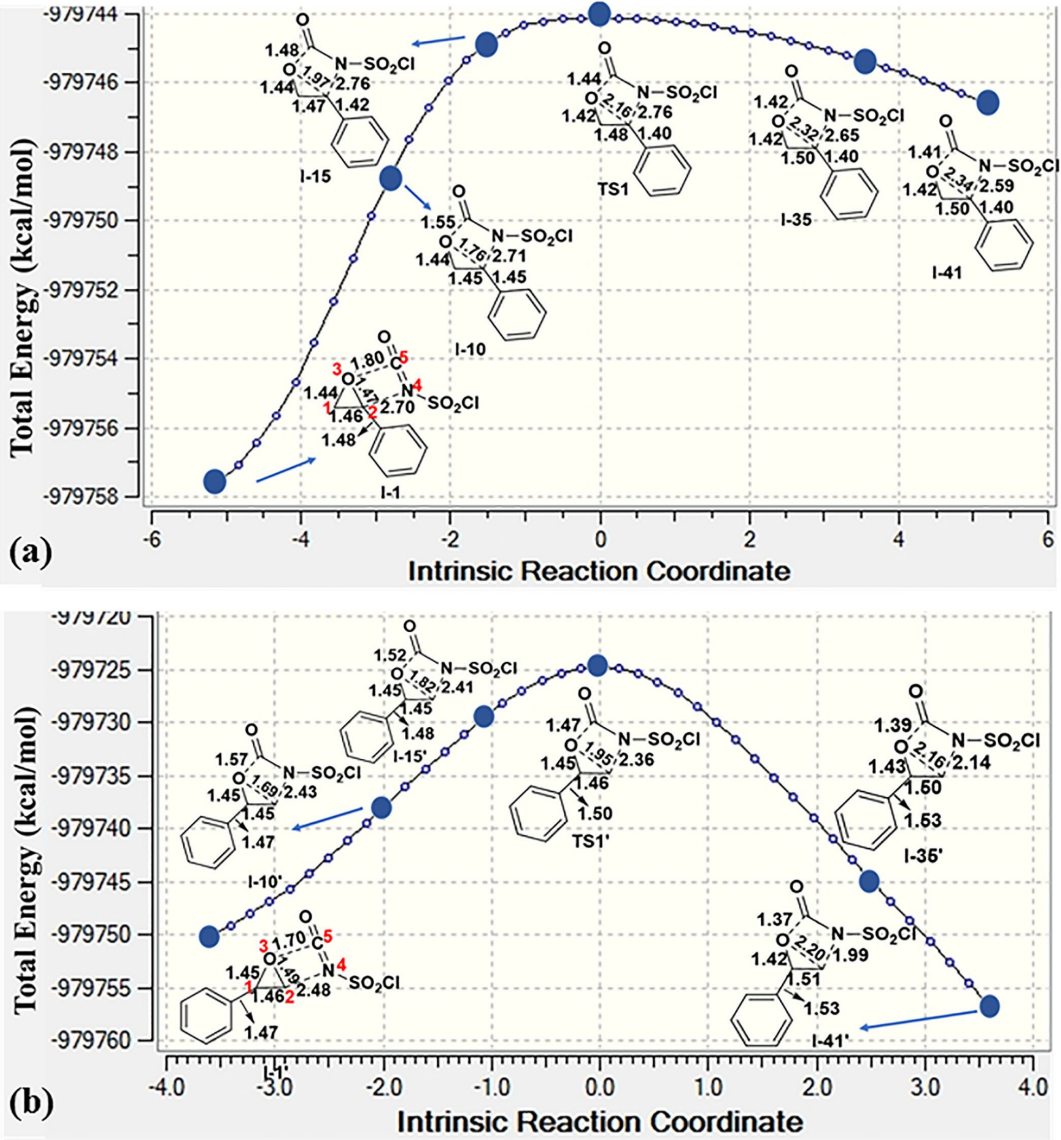
IRC calculated for the formation of (a) **10** and (b) **11** at M06-2X/6-31+G(d,p) level. **I-1**, **I-15**, **I-35**, **I-41**, etc. are the selected points along the coordinate. Distances are given in Å.

The epoxide ring opening and formation of the O–C(=O) bond are almost completed before the C–N bond is formed. The changes in bond lengths along the intrinsic reaction coordinate (IRC) are depicted in Figure 3a and b as an acceptable approach in the literature [57]. For the formation of **10**, the O–C(=O) distance is shortened and C2–O3 bond is elongated rapidly until reaching the product, while the C2–N4 distance is shortened from 2.76 Å in **TS1** to 2.59 Å in **I-41** (Figure 3a). Note that **I-41** is not yet the product but the 41st point in the IRC where the C2–N4 distance will eventually decrease to the bond distance when the number of IRC points are increased. These results refer to asynchronous events. The same trend is observed for the formation of **11** as shown in Figure 3b. Noteworthy, the C2–N4 bond length does not change much along the IRC for the formation of **10**; however, it is shortened more rapidly to give **11**. The presence of partial double bond between C2–C(Ph) (benzylic position) allows electron delocalization around the reacting center, which results in stabilization of the transition state and so lowering the activation energy barrier (Figure 3a). On the other hand, stabilization of the benzylic cation is not possible along the IRC path for **TS1′** (Figure 3b), since the bond distance C2–C(Ph) is found as around 1.50 Å showing a single bond character. This can be the main reason for the predominant formation of intermediate **10** which results in the regioselective formation of oxazolidinone **9f**

**Figure 4:**
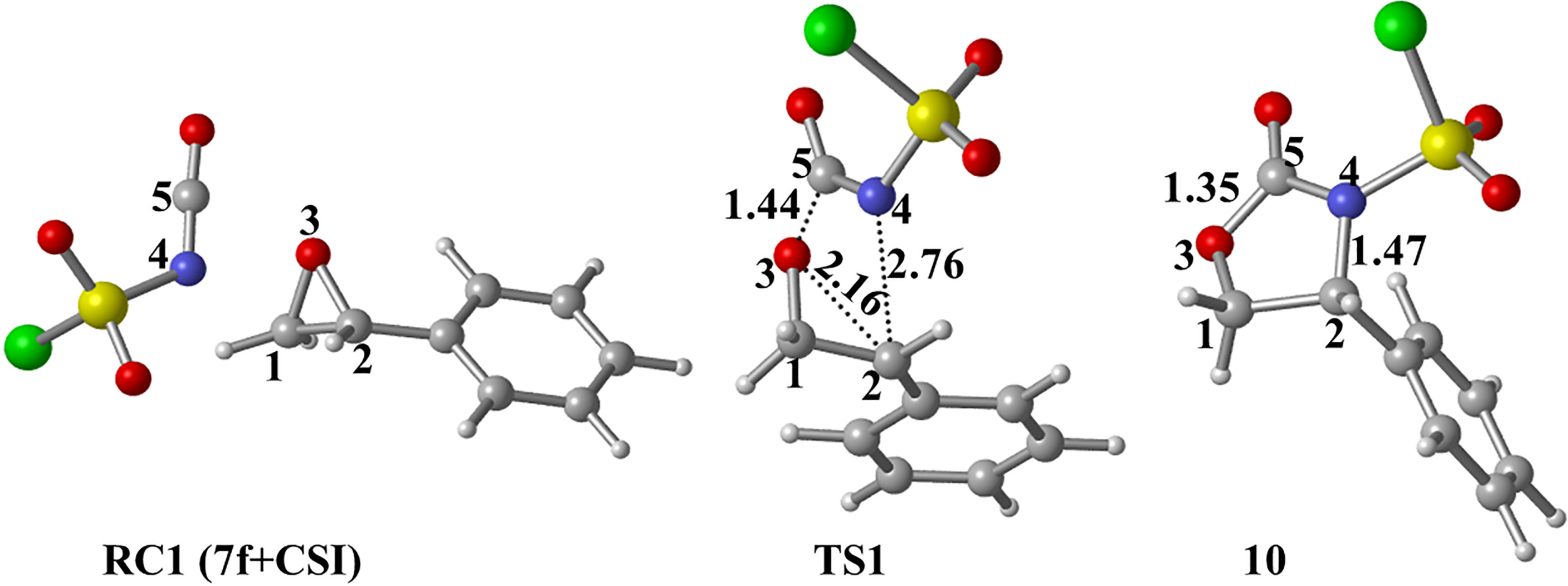
Optimized geometries for the stationary points for the formation of **10** at PCM(DCM)/M06-2X/6-31+G(d,p)//M06-2X/6-31+G(d,p) level (common step of path 1a, path 1b and path 2). Distances are given in Å.

Optimized geometries for reactant complex **RC1 (7f+CSI)**, transition state **TS1** and **10** for the selected path are depicted in Figure 4. This step is common for all paths studied which will be described below.

**Scheme 3:**
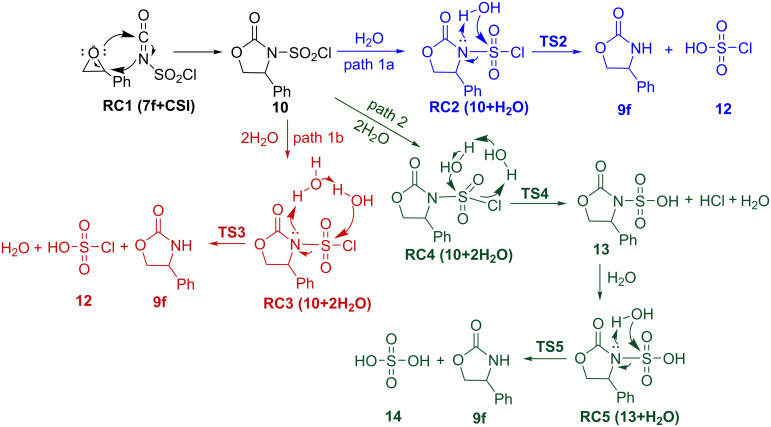
Proposed mechanisms for the formation of oxazolidinone **9f**.

**Figure 5:**
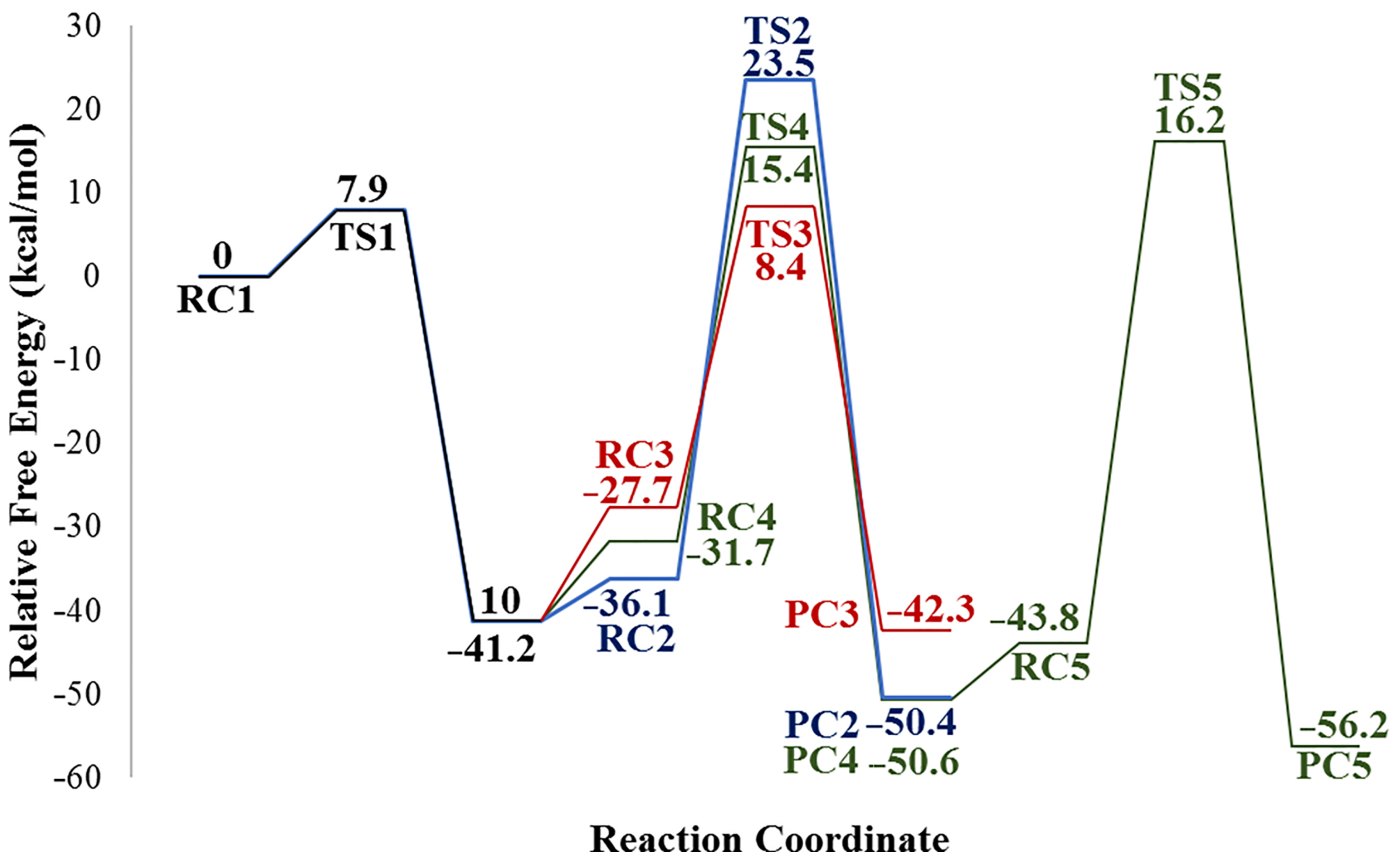
Potential energy profiles for paths 1a (blue), 1b (red), 2 (green) and relative Gibbs free energies (kcal/mol) in DCM related to the formation of **9f** at PCM(DCM)/M06-2X/6-31+G(d,p)//M06-2X/6-31+G(d,p) level.

Once **10** is formed, the next step is addition of water. This step can occur along three different pathways namely path 1a, path 1b and path 2 as shown in Scheme 3. The potential energy profile of each path was generated relative to the energy of the initial reactant complex **RC1 (7f+CSI)** (Figure 5). Paths 1a and 1b represent the protonation of the ring nitrogen by one and two water molecules, respectively, and the departure of **12**. In path 1a, the transformation of the **TS2** to **9f** involves the shortening of the N4–H8 distance from 1.46 to 1.01 Å and S6–O7 distance from 1.89 to 1.54 Å (Supporting Information File 1, Figure S1). This path occurs via the four-membered ring transition state **TS2** with an energy barrier of 23.5 kcal/mol relative to **RC1 (7f+CSI)**.

**Figure 6:**
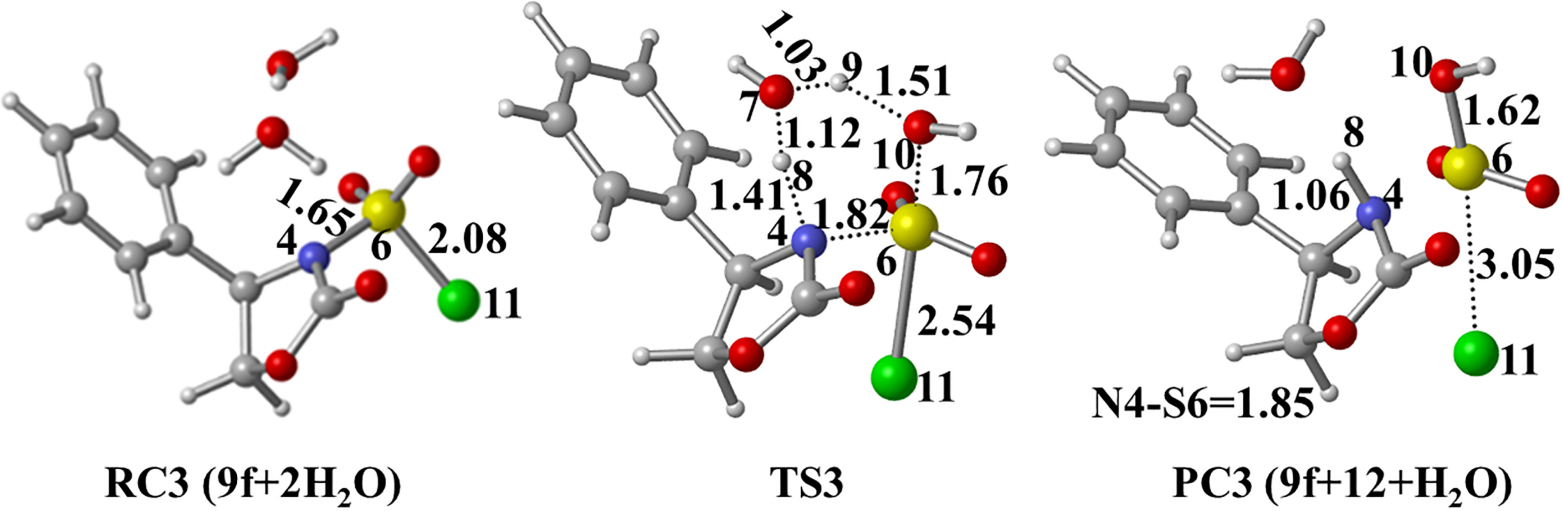
Optimized geometries for the stationary points of path 1b at PCM(DCM)/M06-2X/6-31+G(d,p)//M06-2X/6-31+G(d,p) level. Distances are given in Å.

Another scenario (path 1b) is the direct participation of two water molecules in six-membered **TS3** leading to the target product **9f**. As can be seen from Figure 6, the distance of N4–H8 is calculated as 1.41 Å in **TS3**, which is further shortened to 1.06 Å in **PC3 (9f+12+H_2_O)**. Obviously, the proton shuttle activation mechanism pathway is energetically more favorable, which involves a lower barrier of 8.4 kcal/mol with respect to **RC1 (7f+CSI)** (Figure 5).

Alternatively, the mechanism may involve path 2 where the addition of water molecules to the chlorosulfonyl moiety and the departure of H_2_SO_4_ are observed (Scheme 3). The first step of path 2 involves addition of two water molecules to **RC4 (10+2H_2_O)** resulting in elimination of hydrated HCl and formation of **13**. The calculated free energy of activation was found to be 15.4 kcal/mol with respect to **RC1 (7f+CSI)** (Figure 5). The final step of path 2 takes place from **RC5 (13+H_2_O)** passing through **TS5** and forming the target product **9f**. This step requires an activation free energy of 16.2 kcal/mol with respect to the initial reactant complex **RC1 (7f+CSI)** (Figure 5). The overall process is exothermic by 56.2 kcal/mol. Three-dimensional (3D) views of all the optimized structures of path 2 are illustrated in Supporting Information File 1, Figure S2.

As can be seen from the potential energy profile (Figure 5), water addition to **10** is likely to be the rate-determining step for all reaction pathways. Comparison of the calculated Gibbs free energies of activation in DCM reveals that path 1b is the most plausible mechanism among the paths studied.

**Scheme 4:**
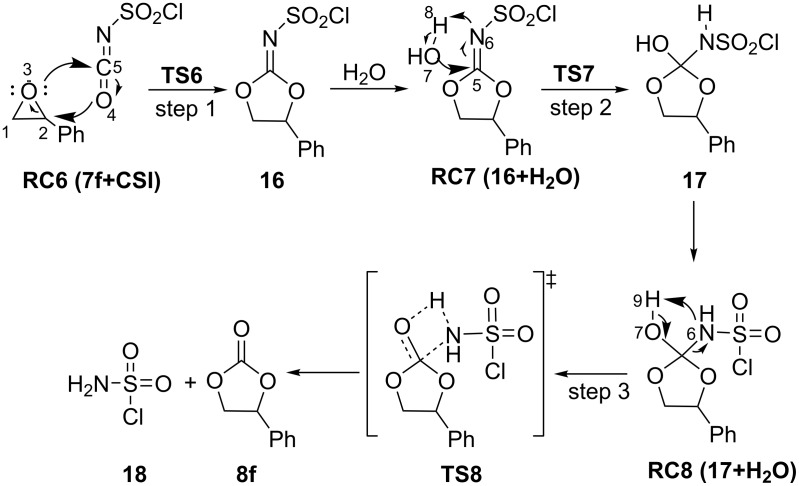
Proposed mechanism for the formation of five-membered cyclic carbonate **8f**.

The reaction mechanism for the formation of five-membered cyclic carbonate **8f** has also been investigated theoretically and it is described below (Scheme 4).

**Figure 7:**
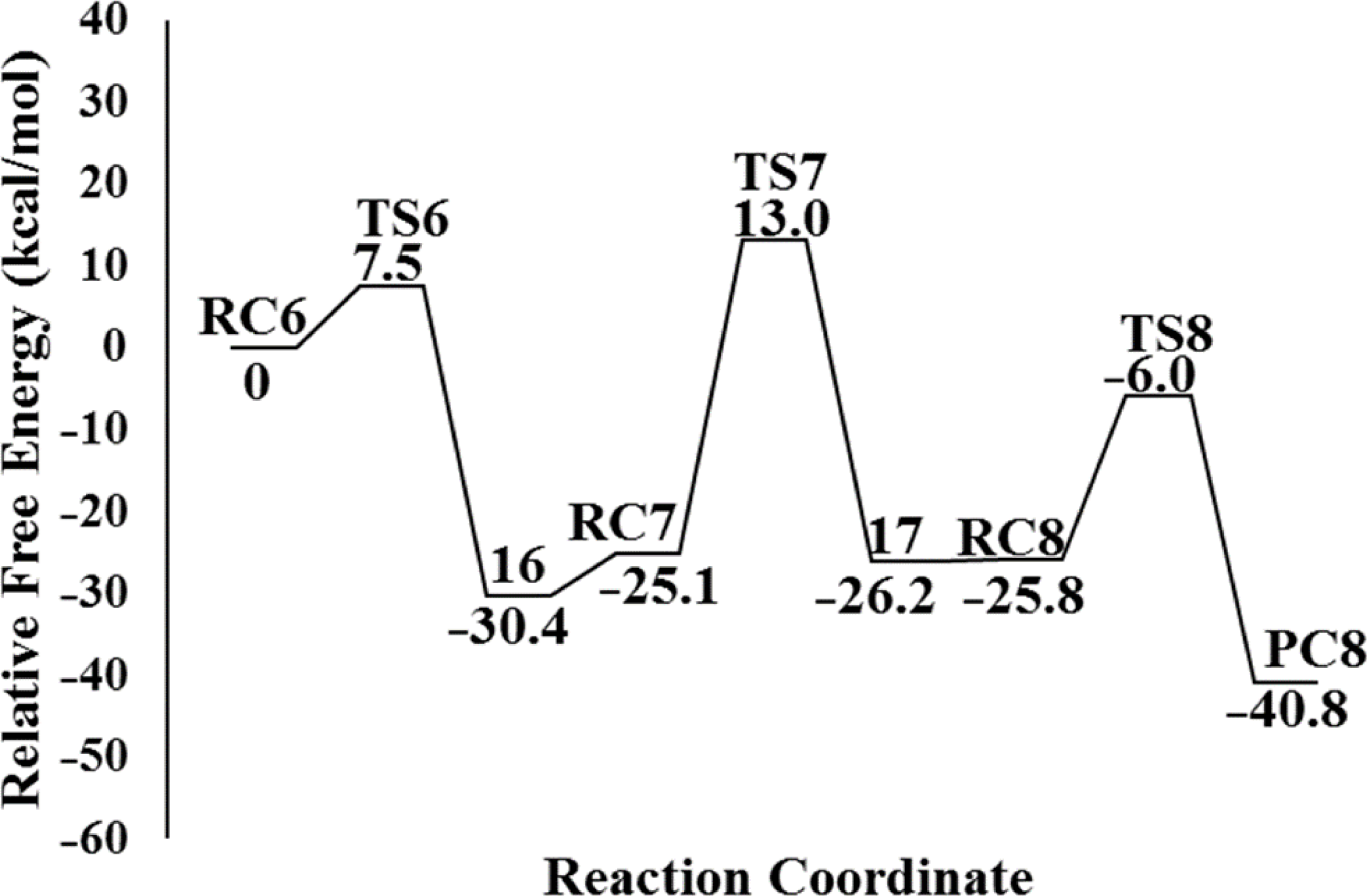
Potential energy profile and relative Gibbs free energies (kcal/mol) in DCM related to the formation of **8f** at PCM(DCM)/M06-2X/6-31+G(d,p)//M06-2X/6-31+G(d,p) level.

**Figure 8:**
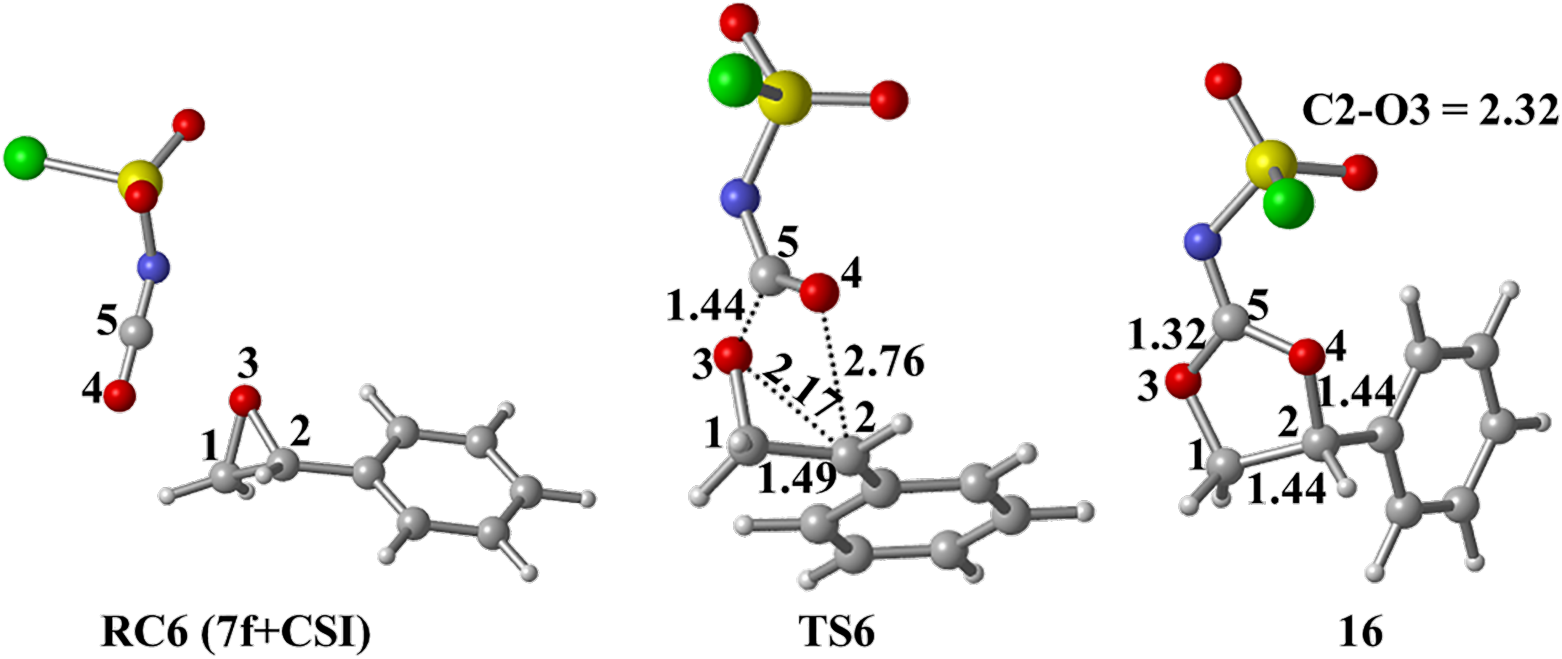
Optimized geometries for the stationary points of step 1 for the formation of **16** at PCM(DCM)/M06-2X/6-31+G(d,p)//M06-2X/6-31+G(d,p) level. Distances are given in Å.

A similar transition state has been proposed for the formation of **8f** in the presence of CSI. The mechanism is thought to proceed by ring opening of the epoxide **7f** at the 2-position, followed by nucleophilic attack of O4 on C2 to afford **16**. The formation of **16** is exergonic by 30.4 kcal/ mol relative to **RC6 (7f+CSI)** (Figure 7). The optimized geometries are illustrated in Figure 8.

**Figure 9:**
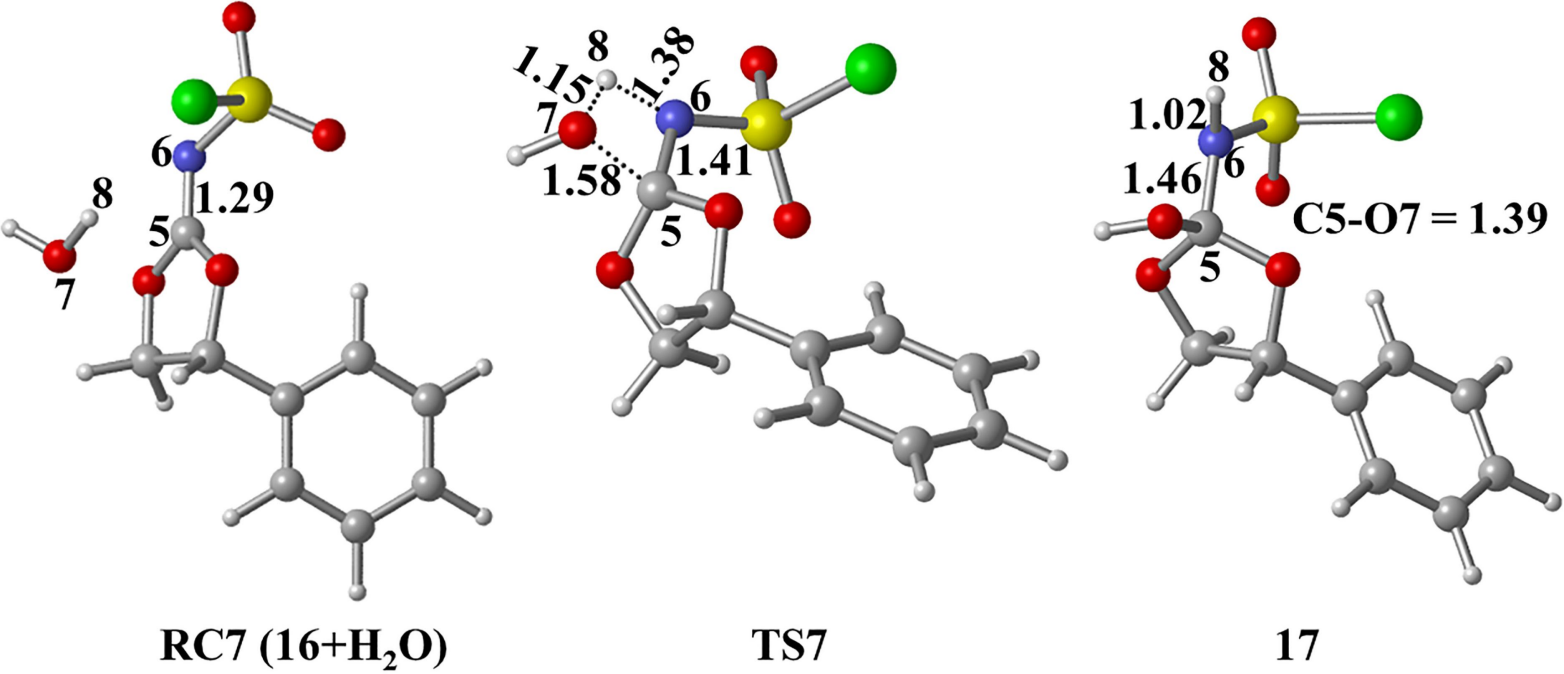
Optimized geometries for the stationary points of step 2 for the formation of **17** at PCM(DCM)/M06-2X/6-31+G(d,p)//M06-2X/6-31+G(d,p) level. Distances are given in Å.

The intermediate **RC7 (16+H_2_O)**, generated by the reaction of CSI with epoxide **7f**, reacts with a water molecule to yield **17**. The bond distance C5–N6 is predicted as 1.29, 1.41, and 1.46 Å in structures **RC7 (16+H_2_O)**, **TS7**, and **17**, respectively (Figure 9). Besides, the C5−O7 distance is 1.58 Å in **TS7**; it is shortened to 1.39 Å in **17**. Here, while the O7–H8 single bond is broken, the N6−H8 bond is formed. The corresponding barrier was calculated to be 13.0 kcal/mol relative to initial reactant complex **RC6 (7f+CSI)** (Figure 7).

**Figure 10:**
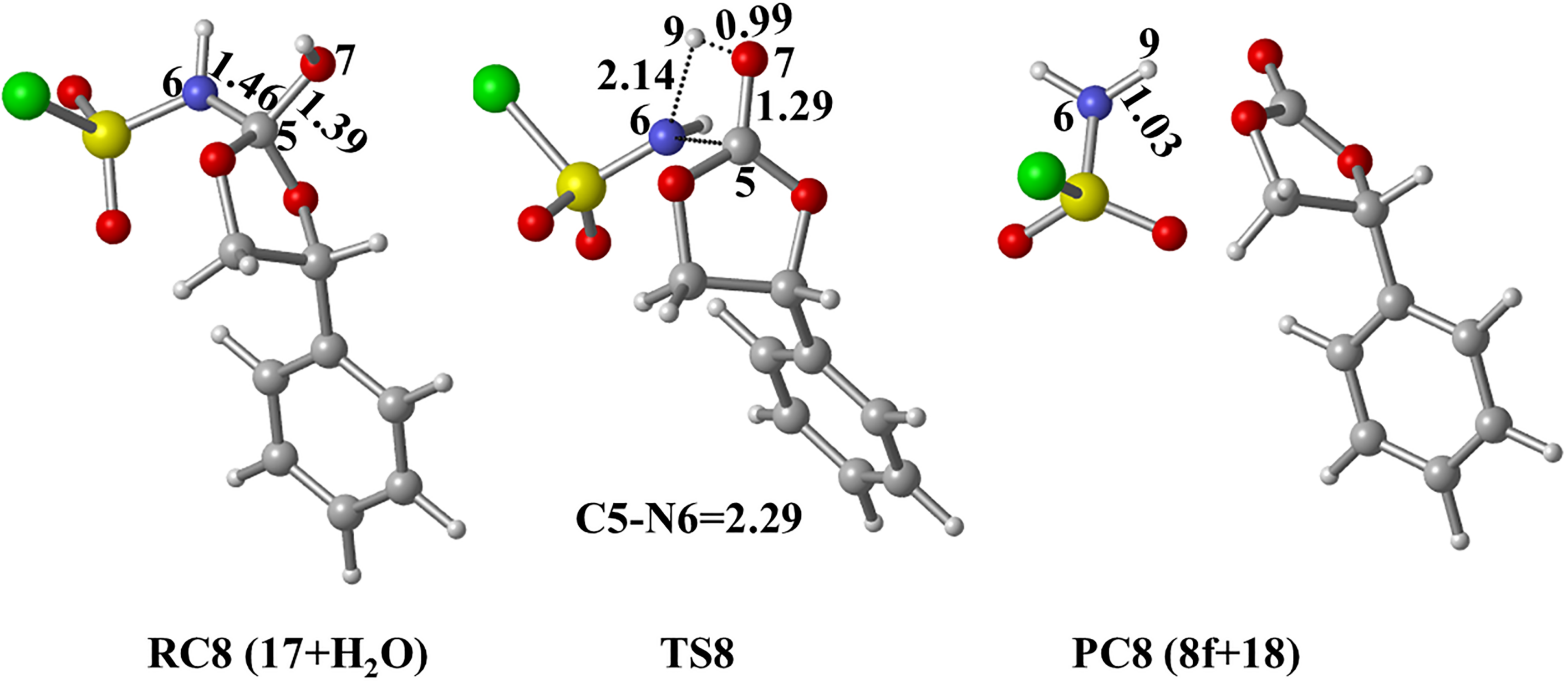
Optimized geometries for the stationary points of step 3 for the formation of **PC8** at PCM(DCM)/M06-2X/6-31+G(d,p)//M06-2X/6-31+G(d,p) level. Distances are given in Å.

Elimination of **18**, accompanied by C=O bond formation, constitutes the final step of the reaction observed. Optimized structures are given in Figure 10. The elimination reaction, via the transition state **TS8**, is facile and leads to the stable product, the five-membered cyclic carbonate **8f** (Figure 7).

In the experimental studies for the reaction of epoxides with CSI, Keshava Murthy and Dhar [41] suggested a stepwise reaction passing through a zwitterionic intermediate (Scheme 2a). De Meijere and co-workers [42] proposed a two-step process involving a 1,5-dipolar intermediate (Scheme 2b). However, in this work, we introduce a new mechanism by providing computational evidence for the asynchronous concerted pathway for the first addition step of epoxides to CSI. Previous computational studies [37,47] involve reactions of CSI with substrates other than epoxides and therefore they are not directly comparable to our reaction; however, they proposed that the reactions of isocyanates may take place through a concerted mechanism. These results are consistent with our computational findings.

## Conclusion

In summary, in the first part of the study, we have improved the general synthesis of five-membered cyclic carbonates and oxazolidinones from various epoxides under mild conditions. We also described the synthesis of novel oxazolidinone derivatives **8i** and **8j** and 1,3-dioxolan-2-ones **9c** and **9i**. Moreover, an effective and simplistic procedure for the synthesis of known compounds **8a–h**, **9a**,**b**, **9d–h** and **9j** has been reported. Compared to the existing methods in the literature, this versatile conversion has enabled us to create a wide range of cyclic carbonates and oxazolidinones in ratios close to 1:1 using a safe, inexpensive, metal-free reagent, a simple purification method and shorter reaction times via a one-pot reaction. The study presents a useful method for one-pot conversion of epoxides to protected 1,2-diols and 2-amino alcohols in one reaction.

In the computational part of the study, the mechanisms leading to oxazolidinone **9f** and cyclic carbonate **8f** were examined. The calculated energy difference between the **TS1** (leading to **9f**) and **TS6** (leading to **8f**) is very small (0.5 kcal/mol) but slightly in favor of carbonate **8f** which is in very good agreement with the experimental observation that isolated yields are 49% for **8f** and 42% for **9f**. The potential energy profiles of the formation of **8f** and **9f** are quite similar. IRC calculations revealed that the first step of the mechanisms for the formation of **8f** and **9f** occur asynchronously although in a concerted fashion. The water addition steps are likely to be rate-determining for both reaction mechanisms. Besides, explicit inclusion of water molecules is crucial for lowering the energy barrier making the process plausible without changing the nature of the rate determining step of the formation of **9f**.

Our computational results adequately explain the relative yields and confirm the product ratio detected in the experiment as well as the regioselectivity in oxazolidinones The proposed mechanisms in this study confirm the product ratio detected in the experiment. The computational findings provided insight into the formation of experimentally observed oxazolidinone **9f** since its precursor intermediate **10** has a remarkably lower activation barrier compared to **11**.

## Methodology

All calculations have been carried with the Gaussian 09 program package [58]. Geometry optimizations of all the minima and transition states involved have been performed using M06-2X [59–60] /6-31+G(d,p) level of theory. The M06-2X functional is known to show good performance in predicting the activation energies and transition state geometries of various reactions [59–61]. Harmonic vibrational frequencies have been calculated at the same level of theory for all stationary points to verify whether they are minima (no imaginary frequencies) or transition states (a single imaginary frequency). Thermodynamic calculations have been performed at 25 °C and 1 atm. The same level of intrinsic reaction coordinate (IRC) [62–63] calculations have been performed to check the energy profiles connecting each transition state to the two associated minima. The effect of the solvent environment on the reaction pathways has been taken into account by single-point energy calculations on the gas-phase stationary points using a polarizable continuum model (PCM) [64] at M06-2X/6-31+G(d,p) level. Structural representations were generated using CYLView [65].

## Experimental

### General considerations

The epoxides were synthesized from related alkenes with *m*-CPBA and purified in a filter column. All solvents and reagents were used as purchased from commercial suppliers without any purification. Melting points were determined on a melting-point apparatus (Gallenkamp; WA11373) and are uncorrected. IR spectra were obtained from solutions in 0.1 mm cells and in CH_2_Cl_2_ with a Perkin–Elmer spectrophotometer. ^1^H and ^13^C NMR spectra were recorded on Varian and Bruker spectrometers at 400 and 100 MHz, respectively, and NMR shifts are presented as δ in ppm. Elemental analyses were performed on a LECO CHNS-932 apparatus. MS spectra were carried out on an LC–MS high-resolution time of flight (TOF) Agilent 1200/6530 instrument. All column chromatography was performed on silica gel (60-mesh, Merck).

### General procedure for the synthesis of five-membered cyclic carbonates and oxazolidinones

Epoxide **7a** (500 mg, 4.54 mmol, 1 equiv) was dissolved in 20 mL dichloromethane. The reaction mixture was cooled to 0 °C, and chlorosulfonyl isocyanate (CSI, 707 mg, 4.99 mmol, 1.1 equiv) was added. The resulting solution was stirred at room temperature for 1 h. Then, water was added to the reaction mixture (2 mL), and the mixture was stirred for 0.5 h. The reaction mixture was extracted with dichloromethane (3 × 20 mL). The organic phase was dried over sodium sulfate and concentrated. Purification was performed through column chromatography on silica gel eluting with hexane/EtOAc (4:1). In all reactions, 1,3-dioxolan-2-ones (**8a–j**) were isolated as the first fraction and oxazolidinones, (**9a–j)** as the second fraction.

**Octahydrocycloocta[*d*]oxazol-2(3*H*)-one (9c):** Colourless solid, *R*_f_ = 0.4 (EtOAc/hexanes, 1:5); mp 91–93 °C; (268 mg, yield 40%); ^1^H NMR (400 MHz, CDCl_3_, ppm) δ 4.70–4.65 (m, 1H, CH-O), 4.57–4.53 (m, 1H, CH-N), 2.28–0.95 (m, 12H, 6×CH_2_); ^13^C NMR (100 MHz, CDCl_3_, ppm) δ 148.9 (C=O), 80.7 (C-O), 65.7 (C-N), 26.9 (CH_2_), 25.3 (CH_2_), 28.4 (CH_2_), 25.3 (CH_2_), 24.9 (CH_2_), 24.2 (CH_2_); IR (CHCl_3_, cm^−1^): 3251, 2928, 2863, 1805, 1410, 1358, 1210, 1175, 1034; anal. calcd for: C, 63.88; H, 8.93; N, 8.28; found: 63.56; H, 9.04; N, 8.54; HRMS–ESI (*m*/*z*): [M + H]^+^ calcd for C_9_H_15_NO_2_^+^, 169,1097; found, 169,1086.

**3a,4,9,9a-Tetrahydro-4,9-methanonaphtho[2,3-*d*][1,3]dioxol-2-one (8i):** Colourless solid, *R*_f_ = 0.5 (EtOAc/hexanes, 1:5); mp 102–104 °C. (287 mg, yield 43%); ^1^H NMR (400 MHz, CDCl_3_, ppm) δ 7.27–7.18 (m, 4H, ArH), 4.62 (s, 2H, CH-O), 3.62 (s, 2H, CH), 2.16 (s, 2H, CH_2_); ^13^C NMR (100 MHz, CDCl_3_, ppm) δ 154.0 (C=O), 142.2 (Ar), 128.1 (Ar), 123.3 (Ar), 80.5 (C-O), 47.9 (CH), 41.6 (CH_2_); IR (CHCl_3_, cm^−1^): 2932, 1804, 1460, 1160, 1066, 976; anal. calcd for: C, 71.28; H, 4.98; found: C, 71.43; H, 4.73; HRMS–ESI (*m*/*z*): [M + H]^+^ calcd for C_12_H_10_O_3_^+^, 202,0624; found, 202,0637.

**3a,4,9,9a-Tetrahydro-4,9-methanonaphtho[2,3-*d*]oxazol-2(3*H*)-one (9i):** Colourless solid, *R*_f_ = 0.3 (EtOAc/hexanes, 1:5); mp 133–135 °C. (235 mg, yield 37%); ^1^H NMR (400 MHz, CDCl_3_, ppm) δ 7.30–7.20 (m, 4H, ArH). 4.94 (s, 1H, CH-O), 3.95 (s, 1H, CH-N), 2.27–2.24 (m, 2H, CH), 2.17–2.11 (m, 2H, CH_2_); ^13^C NMR (100 MHz, CDCl_3_, ppm) δ 162.7 (C=O), 141.4 (Ar), 128.5 (Ar), 123.6 (Ar), 85.1 (Ar), 57.8 (C-O), 47.9 (C-N), 41.9 (CH), 29.9 (CH_2_); IR (CHCl_3_, cm^−1^): 3340, 2918, 1802, 1647, 1461, 1368, 1166, 1001; anal. calcd for: C, 71.63; H, 5.51; N, 6.96; found: C, 71.48; H, 5.72; N, 6.79; HRMS–ESI (*m*/*z*): [M + H]^+^ calcd for C_12_H_11_NO_2_^+^, 201,0784; found, 201,0796.

**3a-Phenylhexahydrobenzo[*d*][1,3]dioxol-2-one (8j):** Colourless solid, *R*_f_ = 0.4 (EtOAc/hexanes, 1:5); mp 97–99 °C; (275 mg, yield 44%); ^1^H NMR (400 MHz, CDCl_3_, ppm) δ 7.42–7.27 (m, 5H, ArH), 4.81 (t, *J* = 4.4 Hz, 1H, CH-O), 2.18–2.09 (m, 8H 4×CH_2_); ^13^C NMR (100 MHz, CDCl_3_, ppm) δ 154.7 (C=O), 140.9 (Ar), 129.1 (Ar), 128.8 (Ar), 124.9 (Ar), 85.2 (C-O), 81.2 (C-O), 34.9 (CH_2_), 26.7 (CH_2_), 19.7 (CH_2_), 18.2 (CH_2_); IR (CHCl_3_, cm^−1^): 2935, 2865, 1803, 1449, 1205, 1028; anal. calcd for: C, 71.54; H, 6.47; found: C, 71.65; H, 6.38; HRMS–ESI (*m*/*z*): [M + H]^+^ calcd for C_13_H_14_O_3_^+^, 218,0937; found, 218,0946.

## Supporting Information

File 1 Experimental, analytical and calculated data.
