# Supplementary material for: One-pot synthesis of oxazolidinones and five-membered cyclic carbonates from epoxides and chlorosulfonyl isocyanate: theoretical evidence for an asynchronous concerted pathway
Source: Beilstein J Org Chem. 2020 Jul 21;16:1805–19. doi: 10.3762/bjoc.16.148 (PMC7385335; doi:10.3762/bjoc.16.148)
Supplement: File 1 — Experimental, analytical and calculated data. [file Beilstein_J_Org_Chem-16-1805-s001.pdf]

BEILSTEIN JOURNAL OF ORGANIC CHEMISTRY

Supporting Information

for

One-pot synthesis of oxazolidinones and five-membered cyclic carbonates from epoxides and chlorosulfonyl isocyanate: theoretical evidence for an asynchronous concerted pathway

Esra Demir, Ozlem Sari, Yasin Çetinkaya, Ufuk Atmaca, Safiye Sağ Erdem and Murat Çelik

Beilstein J. Org. Chem. **2020,** *16,* 1805–1819. doi:10.3762/bjoc.16.148

Experimental, analytical and calculated data

License and Terms: This is a supporting information file under the terms of the Creative Commons Attribution License (http://creativecommons.org/licenses/by/4.0). Please note that the reuse, redistribution and reproduction in particular requires that the authors and source are credited. The license is subject to the *Beilstein Journal of Organic Chemistry* terms and conditions: (https://www.beilstein-journals.org/bjoc)

Contents **Page**

Experimental ……………………………………………………………………………..S2
General remarks ………………………………………………………………………….S2
General procedure synthesis of epoxidation ...…………………………………………...S2
^{1}H NMR and ^{13}C NMR spectra of **8a** ………………………………………………………..S6
^{1}H NMR and ^{13}C NMR spectra of **9a** ……………………………………………………..….S7
^{1}H NMR and ^{13}C NMR spectra of **8b** ………………………………………………………….S8
^{1}H NMR and ^{13}C NMR spectra of **9b** ……………………………………………………......S9
^{1}H NMR and ^{13}C NMR spectra of **8c** …………………………………………………………S10
^{1}H NMR and ^{13}C NMR spectra of **9c** ……………………………………..………...S11
^{1}H NMR and ^{13}C NMR spectra of **8d** …………………………………………………………S12
^{1}H NMR and ^{13}C NMR spectra of **9d** ………………………………………………………..S13
^{1}H NMR and ^{13}C NMR spectra of **8e** …………………………………………………………S14
^{1}H NMR and ^{13}C NMR spectra of **9e** ……………………………………………………..…S15
^{1}H NMR and ^{13}C NMR spectra of **8f** …………………………………………………………..S16
^{1}H NMR and ^{13}C NMR spectra of **9f** …………………………………………………………..S17
^{1}H NMR and ^{13}C NMR spectra of **8g** ………………………………………………………….S18
^{1}H NMR and ^{13}C NMR spectra of **9g** ………………………………………………………….S19
^{1}H NMR and ^{13}C NMR spectra of **8h** ………………………………………………………….S20
^{1}H NMR and ^{13}C NMR spectra of **9h** ………………………………………………………….S21
^{1}H NMR and ^{13}C NMR spectra of **8i** ………………………………………………………….S22
^{1}H NMR and ^{13}C NMR spectra of **9i** ………………………………………………………….S23
^{1}H NMR and ^{13}C NMR spectra of **8j** ……………………………………………………......S24
^{1}H NMR and ^{13}C NMR spectra of **9j** …………………………………………………………..S25
Figure S1. Optimized geometries for the stationary points of path 1a at PCM(DCM)/M06-2X/6-31+G(d,p)//M06-2X/6-31+G(d,p) level. Distances are given in Å…………………...S26
Figure S2. Optimized geometries for the stationary points of path 2 at PCM(DCM)/M06-2X/6-31+G(d,p)//M06-2X/6-31+G(d,p) level. Distances are given in Å…………………...S26
Table S1. Absolute energies of optimized structures for the formation of **10** and **11** in gas phase **(**M06-2X/6-31+G(d,p)) …………………………………………………...S27
Table S2. Single point energies for the formation of **10** and **11** at PCM(DCM)/M06-2X/6-31+G(d,p)//M06-2X/6-31+G(d,p) level ……………………….S27
Table S3. Absolute energies of optimized structures for path 1a, path 1b and path 2 in gas phase **(**M06-2X/6-31+G(d,p)) …………………………………………………...S27
Table S4. Single point energies for path 1a, path 1b and path 2 at PCM(DCM)/M06-2X/6-31+G(d,p)//M06-2X/6-31+G(d,p) level ..……………………S28
Table S5. Absolute energies of optimized structures for the formation of **8f** in gas phase **(**M06-2X/6-31+G(d,p)) …………………………………………………………..S28
Table S6. Single point energies for the formation of **8f** at PCM(DCM)/M06-2X/6-31+G(d,p)//M06-2X/6-31+G(d,p) level ……………………….S28
Cartesian coordinates for the optimized structures given in paths ………………………S29
References ……………………………………………………………………………S45

EXPERIMENTAL

General remarks

The epoxides were synthesized from related alkenes with *m*-CPBA and purified in the filter column. All solvents and reagents were used as purchased from commercial suppliers without any purification. Melting points were determined on a melting-point apparatus (Gallenkamp; WA11373) and are uncorrected. IR spectra were obtained from solutions in 0.1mm cells and in CH_2Cl_2 with a Perkin–Elmer spectrophotometer. ^1H and ^{13}C NMR spectra were recorded on Varian and Bruker spectrometers at 400 and 100 MHz, respectively, and NMR shifts are presented as δ in ppm. Elemental analyses were performed on LECO CHNS-932 apparatus. MS spectra were carried out on an LC/MS High-Resolution Time of Flight (TOF) Agilent 1200/6530 instrument. All column chromatography was performed on silica gel (60-mesh, Merck).

General procedure for the synthesis of epoxides:

1 g of the alkene was dissolved in CH_2Cl_2 (40 mL) and cooled to 0 ºC. Then metachloroperbenzoic acid (1.2 equiv) was slowly added to the solution. The solution was left stirring for 12 h at ambient temperature. The solution was washed with 1 M Na_2SO_3 (3 × 30 mL, 1 M), a saturated solution of $NaHCO_3$ (3 × 30 mL) and brine. The organic phase was dried over sodium sulfate and the product obtained after removal of the solvent in vacuo (rotary evaporator), and purified by the column chromatography.

Hexahydro-4,7-methanobenzo[*d*][1,3]dioxol-2-one (8a): [48] [1] Colourless solid, (295 mg, yield 42%). ^1H NMR (400 MHz, $CDCl_3$, ppm) δ 4.51 (d, *J* = 1.46 Hz, 2H), 2.51-2.53 (m, 2H), 1.10-1.75 (m, 6H); ^{13}C NMR (100 MHz, $CDCl_3$, ppm) δ 155.9, 82.5, 40.4, 31.0, 23.0; IR ($CHCl_3$, cm^{-1}): 2969, 2879, 1802, 1784, 1372, 1164, 1068; Elemental Analysis Calcd for: C, 62.33; H, 6.54; found: 62.48; H, 6.42. HRMS (ESI) m/z calcd for $C_8H_{10}O_3$+: 154,0624; found [M+H]+ 154,0642.

Hexahydro-4,7-methanobenzo[*d*]oxazol-2(3*H*)-one (9a): [49] [2] Colourless solid, (263 mg, yield 38%). ^1H NMR (400 MHz, $CDCl_3$, ppm) δ 4.80 (s, 1H), 3.92 (s, 2H), 2.68 (m, 2H). 1.93-0.83 (m, 6H); ^{13}C NMR (100 MHz, $CDCl_3$, ppm) δ 157.2, 86.5, 57.6, 40.4, 31.3, 22.5; IR ($CHCl_3$, cm^{-1}): 3366, 2961, 2930, 2885, 1801, 1781, 1709, 1644, 1351, 1182, 1067, 1001; Elemental Analysis Calcd for: C, 62.73; H, 7.24; N, 9.14; found: 62.64; H, 7.49; N, 9.32; HRMS (ESI) m/z calcd for $C_8H_{11}NO_2$+: 153,0784; found [M+H]+ 153,0765.

Hexahydro-4*H*-cyclohepta[*d*][1,3]dioxol-2-one (8b): [50] [3]Colourless solid, (334 mg, yield 48%). ^{1}H NMR (400 MHz, $CDCl_3$, ppm) δ 4.82 (m, 2H), 1.94 (m, 4H), 1.80 (m, 2H), 1.63 (m, 1H), 1.50 (m, 1H), 1.32 (m, 2H); ^{13}C NMR (100 MHz, $CDCl_3$, ppm) δ 154.5, 79.6, 30.1, 29.7, 23.2; IR ($CHCl_3$, cm^{-1}): 2930, 2860, 1798, 1721, 1456, 1378, 1180, 1180, 1052; Elemental Analysis Calcd for: C, 61.52; H, 7.74; found: 61.58; H, 7.52; HRMS (ESI) m/z calcd for $C_8H_{12}O_3$+: 156,0781; found [M+H]+ 156,0769.

Octahydro-2*H*-cyclohepta[*d*]oxazol-2-one (9b): [51] [4] Colourless solid, (315 mg, yield 45%). ^{1}H NMR (400 MHz, $CDCl_3$, ppm) δ 4.86-4.92 (m, 1H), 4.59-4.36 (m, 1H), 2.32-1.21 (m, 10H), ^{13}C NMR (100 MHz, $CDCl_3$, ppm) δ 150.1, 79.2, 64.2, 30.3, 29.9, 28.5, 25.4, 22.7; IR ($CHCl_3$, cm^{-1}): 3250, 2931, 2859, 1803, 1748, 1414, 1369, 1176, 1087; Elemental Analysis Calcd for: C, 61.91; H, 8.44; N, 9.03; found: 61.73; H, 8.49; N, 9.34; HRMS (ESI) m/z calcd for $C_8H_{13}NO_2$+: 155,0941; found [M+H]+ 155,0964.

Octahydrocycloocta[*d*][1,3]dioxol-2-one (8c): [50] [3] Colourless solid, (342 mg, yield 51%). ^{1}H NMR (400 MHz, $CDCl_3$, ppm) δ 4.72 (m, 2H), 1.99 (m, 4H), 1.73 (m, 2H), 1.48 (m, 4H), 1.25 (m, 2H); ^{13}C NMR (100 MHz, $CDCl_3$, ppm) δ 154.4, 81.4, 26.5, 26.2, 26.1; IR ($CHCl_3$, cm^{-1}): 2924, 1801, 1472, 1359, 1203, 1174, 1054; Elemental Analysis Calcd for: C, 63.51; H, 8.29; found: 63.42; H, 8.39; HRMS (ESI) m/z calcd for $C_9H_{14}O_3$+: 170,0937; found [M+H]+ 170,0956.

***trans*-4,5-Diphenyl-1,3-dioxolan-2-one (8d):** [50] [3] Colourless solid, (263 mg, yield 43%). ^{1}H NMR (400 MHz, $CDCl_3$, ppm) δ 5.42 (s, 2H), 7.31-7.33 (m, 3H), 7.43-7.45 (m, 6H). ^{13}C NMR (100 MHz, $CDCl_3$, ppm) δ 154.3, 135.1, 130.0, 129.5, 126.3, 85.6; IR ($CHCl_3$, cm^{-1}): 3064, 2925, 1808, 1457, 1257, 1167, 1044; Elemental Analysis Calcd for: C, 74.99; H, 5.03; found: C, 75.32; H, 5.21; HRMS (ESI) m/z calcd for $C_{15}H_{12}O_3$+: 240,0781; found [M+H]+ 240,0796.

***trans*-4,5-Diphenyloxazolidin-2-one (9d):** [52] [5]Colourless solid, (208 mg, yield 34%). ^{1}H NMR (400 MHz, $CDCl_3$, ppm) δ 7.39-7.42 (m, 6H) 7.25-7.32 (m, 4H), 5.48 (bs, 1H), 5.30 (m, 1H), 4.75 (d, J = 7.7 Hz, 1H); ^{13}C NMR (100 MHz, $CDCl_3$, ppm) δ 158.7, 138.5, 137.5, 129.5, 129.3, 129.2, 128.5, 126.7, 126.1, 86.4, 65.1; IR ($CHCl_3$, cm^{-1}): 3290, 2923, 2853, 1754, 1456, 1380, 1199, 1015; Elemental Analysis Calcd for: C, 75.30; H, 5.48; N, 5.85; found: C, 75.42; H, 5.36; N, 5.67; HRMS (ESI) m/z calcd for $C_{15}H_{13}NO_2$+; 239,0941; found [M+H]+ 239,0947.

***cis*-4,5-Diphenyl-1,3-dioxolan-2-one (8e):** [50] [3]Colourless solid, (269 mg, yield 44%). ^{1}H NMR (400 MHz, $CDCl_3$, ppm) δ 7.42-7.45 (m, 6H). 7.30-7.33 (m, 4H). 5.44 (s, 2H); ^{13}C NMR (100 MHz, $CDCl_3$, ppm) δ 154.3, 135.0, 130.0, 127.3, 126.3, 85.6; IR ($CHCl_3$, cm^{-1}): 2918, 1811, 1454, 1275,

1091; Elemental Analysis Calcd for: C, 74.99; H, 5.03; found: C, 75.12; H, 5.24; HRMS (ESI) m/z calcd for $C_{15}H_{12}O_3$+: 240,0781; found [M+H]+ 240,0794.

***cis*-4,5-Diphenyloxazolidin-2-one (9e):** [52] [5] Colourless solid, (250 mg, yield 41%). ^{1}H NMR (400 MHz, $CDCl_3$, ppm) δ 7.12-7.09 (m, 6H), 6.98-6.94 (m, 4H), 5.95 (d, *J* = 8.1 Hz, 1H), 5.5 (bs, 1H), 5.2 (d, *J* = 8.1 Hz, 1H); ^{13}C NMR (100 MHz, $CDCl_3$, ppm) δ 160.1, 136.2, 128.6, 128.5, 128.3, 128.1, 127.2, 126.7, 126.3, 126.0, 82.6, 61.6; IR ($CHCl_3$, cm^{-1}): 3295, 2923, 1754, 1455, 1356, 1217, 1091, 1020; Elemental Analysis Calcd for: C, 75.30; H, 5.48; N, 5.85; found: C, 75.36; H, 5.57; N, 5.66; HRMS (ESI) m/z calcd for $C_{15}H_{13}NO_2$+: 239,0941; found [M+H]+ 239,0955.

4-Phenyl-1,3-dioxolan-2-one (8f): [16] [6] White solid, (355 mg, yield 49%). ^{1}H NMR (400 MHz, $CDCl_3$, ppm) δ 7.27-7.46 (m, 5H), 5.68 (t, *J* = 8.6 Hz, 1H), 4.82 (t, *J* = 8.6 Hz, 1H), 4.36 (t, *J* = 8.6 Hz, 1H); ^{13}C NMR (100 MHz, $CDCl_3$, ppm) δ 155.0, 136.0, 129.5, 129.2, 126.0, 78.2, 71.4; IR ($CHCl_3$, cm^{-1}): 2932, 1809, 1795, 1457, 1358, 1270, 1168, 1067; Elemental Analysis Calcd for: C, 65.85; H, 4.91; found: C, 65.92; H, 4.85; HRMS (ESI) m/z calcd for $C_9H_8O_3$+: 164,0468; found [M+H]+ 164,0475.

4-Phenyloxazolidin-2-one (9f): [16] [6] White solid, (285 mg, yield 42%). ^{1}H NMR (400 MHz, $CDCl_3$, ppm) δ 7.38-7.43 (m, 5H), 5.46 (s, 1H, NH), 4.98 (dd, *J* = 8.6, 7.0 Hz, 1H), 4.76 (dd, *J* = 8.6, 7.0 Hz, 1H), 4.21 (dd, *J* = 8.6, 7.0 Hz, 1H); ^{13}C NMR (100 MHz, $CDCl_3$, ppm) δ 56.6, 72.8, 126.3, 129.2, 129.5, 139.6. 158.2; IR ($CHCl_3$, cm^{-1}): 3284, 2922, 1744, 1457, 1404, 1238, 1040; Elemental Analysis Calcd for: C, 66.25; H, 5.56; N, 8.58; found: C, 66.48; H, 5.32; N, 8.45; HRMS (ESI) m/z calcd for $C_9H_9NO_2$+: 163,0628; found [M+H]+ 163,0643.

4-Benzyl-1,3-dioxolan-2-one (8g): [16] [6] Colourless oil, (270 mg, yield 41%). ^{1}H NMR (400 MHz, $CDCl_3$, ppm) δ 2.99 (dd, *J* = 6.7, 14.2 Hz, 1H), 3.15 (dd, *J* = 6.4, 14.2 Hz, 1H), 4.17 (dd, *J* = 6.9, 8.6 Hz, 1H), 4.44 (t, *J* = 8.1 Hz, 1H), 4.93 (m, 1H), 7.18-7.25 (m, 2H), 7.28-7.36 (m, 3H); ^{13}C NMR (100 MHz, $CDCl_3$, ppm) δ 155.1, 134.1, 129.6, 129.2, 127.8, 77.1, 68.7, 39.8; IR ($CHCl_3$, cm^{-1}): 2921, 1803, 1455, 1371, 1272, 1170, 1080; Elemental Analysis Calcd for: C, 67.41; H, 5.66; found: C, 67.49; H, 5.72; HRMS (ESI) m/z calcd for $C_{10}H_{10}O_3$+: 178,0624; found [M+H]+ 178,0643.

4-Benzyloxazolidin-2-one (9g): [53] [7]Colourless solid, (230 mg, yield 35%). ^{1}H NMR (400 MHz, $CDCl_3$, ppm) δ 7.17-7.35 (m, 5H), 4.84 (bs, 1H), 4.47- 4.49 (m, 1H), 4.07-4.13 (m, 2H), 2.76-2.84 (m, 2H), ^{13}C NMR (100 MHz, $CDCl_3$, ppm) δ 159.6, 135.8, 129.1, 128.9, 127.3, 69.6, 53.7, 41.4; IR ($CHCl_3$, cm^{-1}): 3282, 2925, 1744, 1456, 1410, 1223, 1030; Elemental Analysis Calcd for: C, 67.78; H,

6.26; N, 7.90 found: C, 67.71; H, 6.38; N, 7.75; HRMS (ESI) m/z calcd for $C_{10}H_{11}NO_2$+: 177,0784; found [M+H]+ 177,0789.

3a,8a-Dihydro-8*H*-indeno[1,2-*d*][1,3]dioxol-2-one (8h): [54] [8]White solid, (300 mg, yield 45%). ^{1}H NMR (400 MHz, $CDCl_3$, ppm) δ 7.32-7.53 (m, 4H), 6.01 (d, J = 6.8 Hz, 1H), 5.44-5.46 (m, 1H), 3.40 (m, 2H); ^{13}C NMR (100 MHz, $CDCl_3$, ppm) δ 154.9, 140.3, 136.7, 131.3, 128.5, 126.8, 125.6, 83.7, 79.7, 38.3; IR ($CHCl_3$, cm^{-1}): 2925, 1789, 1463, 1362, 1160, 1058, 1016; Elemental Analysis Calcd for: C, 68.18; H, 4.58; found: C, 68.34; H, 4.65; HRMS (ESI) m/z calcd for $C_{10}H_8O_3$+: 176,0468; found [M+H]+ 176,0478.

3,3a,8,8a-Tetrahydro-2*H*-indeno[1,2-*d*]oxazol-2-one (9h): [52] [5]White solid, (265 mg, yield 40%). ^{1}H NMR (400 MHz, $CDCl_3$, ppm) δ 7.25 –7.36 (m, 4H), 7.10 (bs, 1H), 5.40–5.45 (m, 1H), 5.19 (d, *J* = 7.1 Hz, 1H), 3.42 (d, *J* = 18.5 Hz 1H), 3.34 (d, *J* = 16.9 Hz, 1H); ^{13}C NMR (100 MHz, $CDCl_3$, ppm) δ 159.7, 140.2, 139.6, 129.4, 127.7, 125.4, 124.9, 80.7, 61.3, 38.9; IR ($CHCl_3$, cm^{-1}): 3490, 2921, 1803, 1455, 1272, 1170, 1161; Elemental Analysis Calcd for: C, 68.56; H, 5.18; N, 8.00; found: C, 68.64; H, 5.34; N, 8.23; HRMS (ESI) m/z calcd for $C_{10}H_9NO_2$+: 175,0628; found [M+H]+ 175,0643.

3a-Phenylhexahydrobenzo[*d*]oxazol-2(3*H*)-one (9j): [55] [9]Colourless oil, (265 mg, yield 42%). ^{1}H NMR (400 MHz, $CDCl_3$, ppm) δ 7.42–7.38 (m, 2H), 7.36–7.33 (m, 2H), 7.30–7.26 (m, 1H), 4.66 (t, *J* = 4.2 Hz, 1H), 2.19–2.14 (m, 1H), 2.06–2.01 (m, 1H), 1.89–1.66 (m, 4H), 1.62–1.53 (m, 2H); ^{13}C-NMR (100 MHz, $CDCl_3$, ppm) δ 159.8, 143.3, 128.6, 127.8, 125.3, 82.0, 61.8, 34.8, 25.9, 19.5, 17.6; IR ($CHCl_3$, cm^{-1}): 3220, 2943, 1803, 1448, 1205, 1028, 1005; Elemental Analysis Calcd for: C, 71.87; H, 6.96; N, 6.45; found: C, 71.68; H, 6.91; N, 6.54; HRMS (ESI) m/z calcd for $C_{13}H_{15}NO_2$+: 217,1097; found [M+H]+ 217,1086.

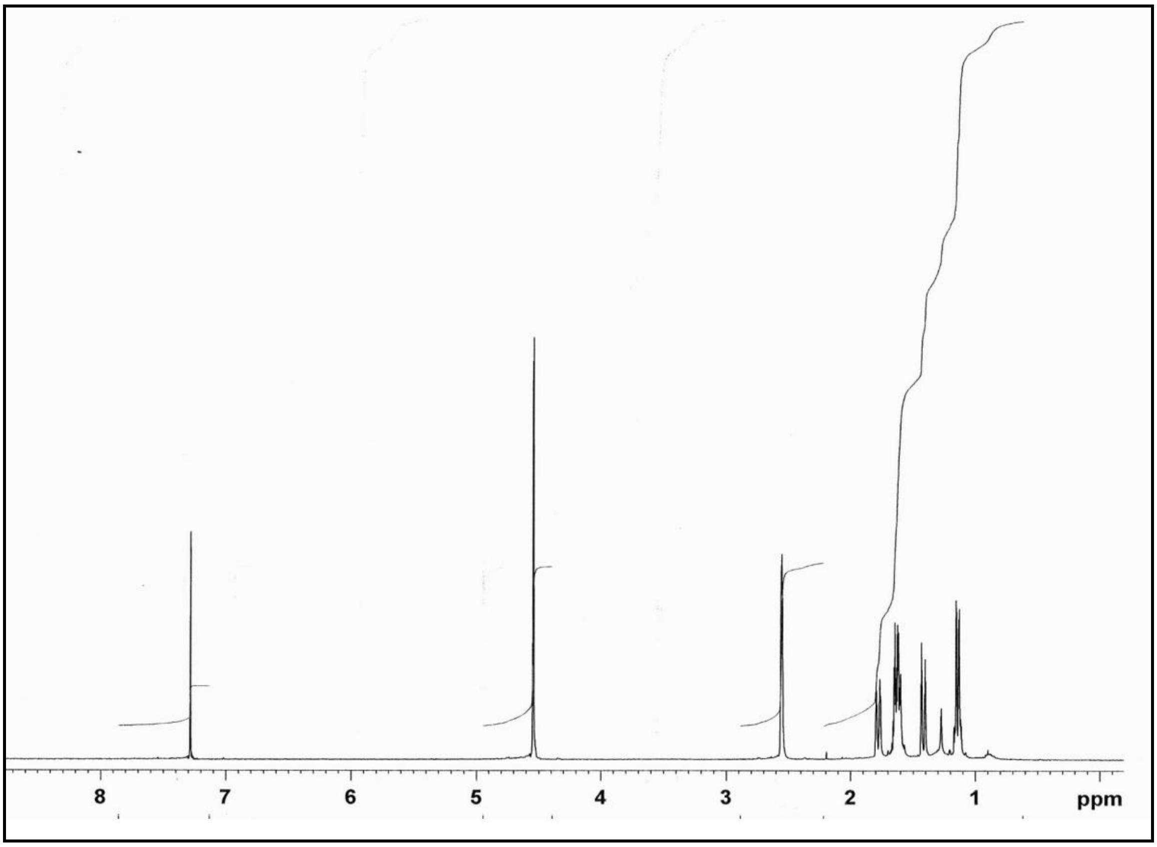

^{1}H NMR spectrum of hexahydro-4,7-methanobenzo[*d*][1,3]dioxol-2-one (**8a**)

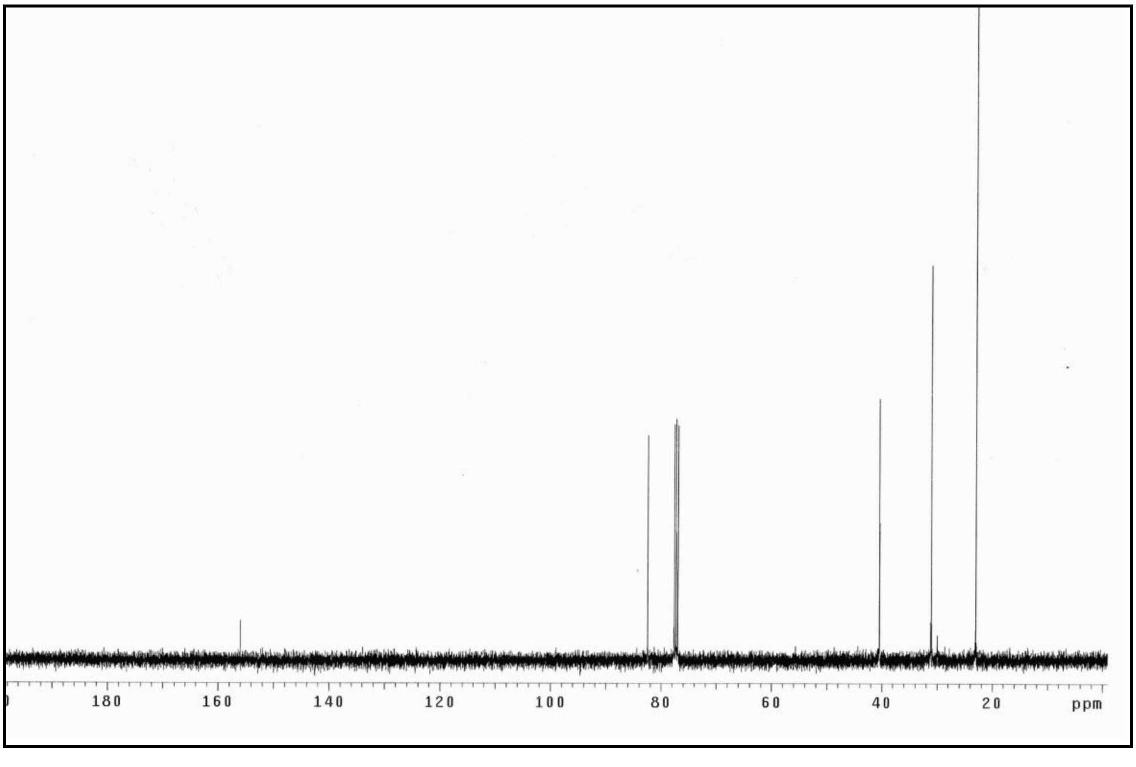

^{13}C NMR spectrum of hexahydro-4,7-methanobenzo[*d*][1,3]dioxol-2-one (**8a**)

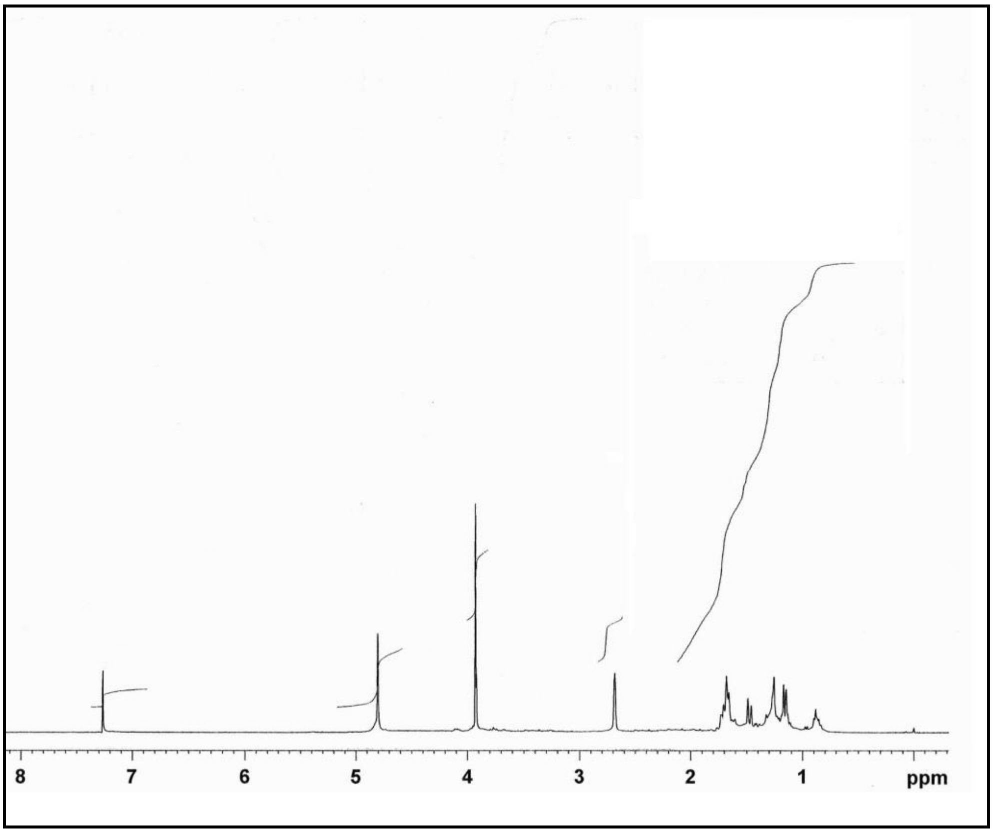

^{1}H NMR spectrum of hexahydro-4,7-methanobenzo[*d*]oxazol-2(3*H*)-one (**9a**)

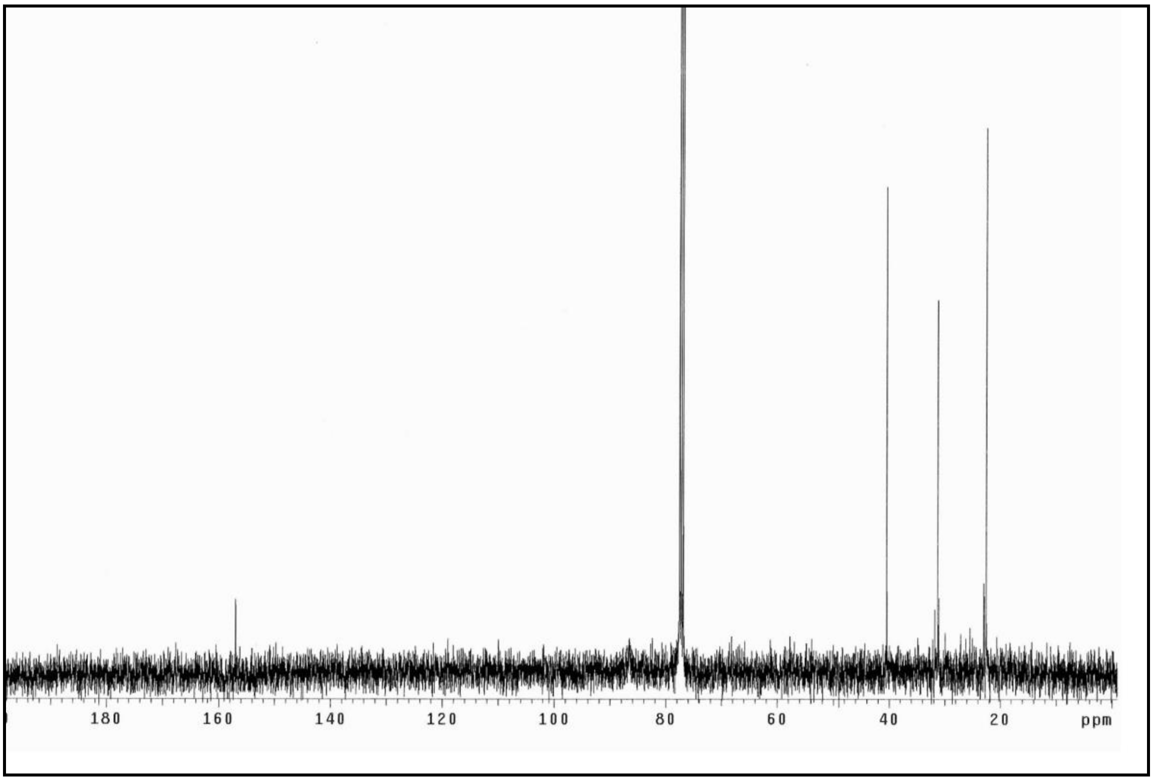

^{13}C NMR spectrum of hexahydro-4,7-methanobenzo[*d*]oxazol-2(3*H*)-one (**9a**)

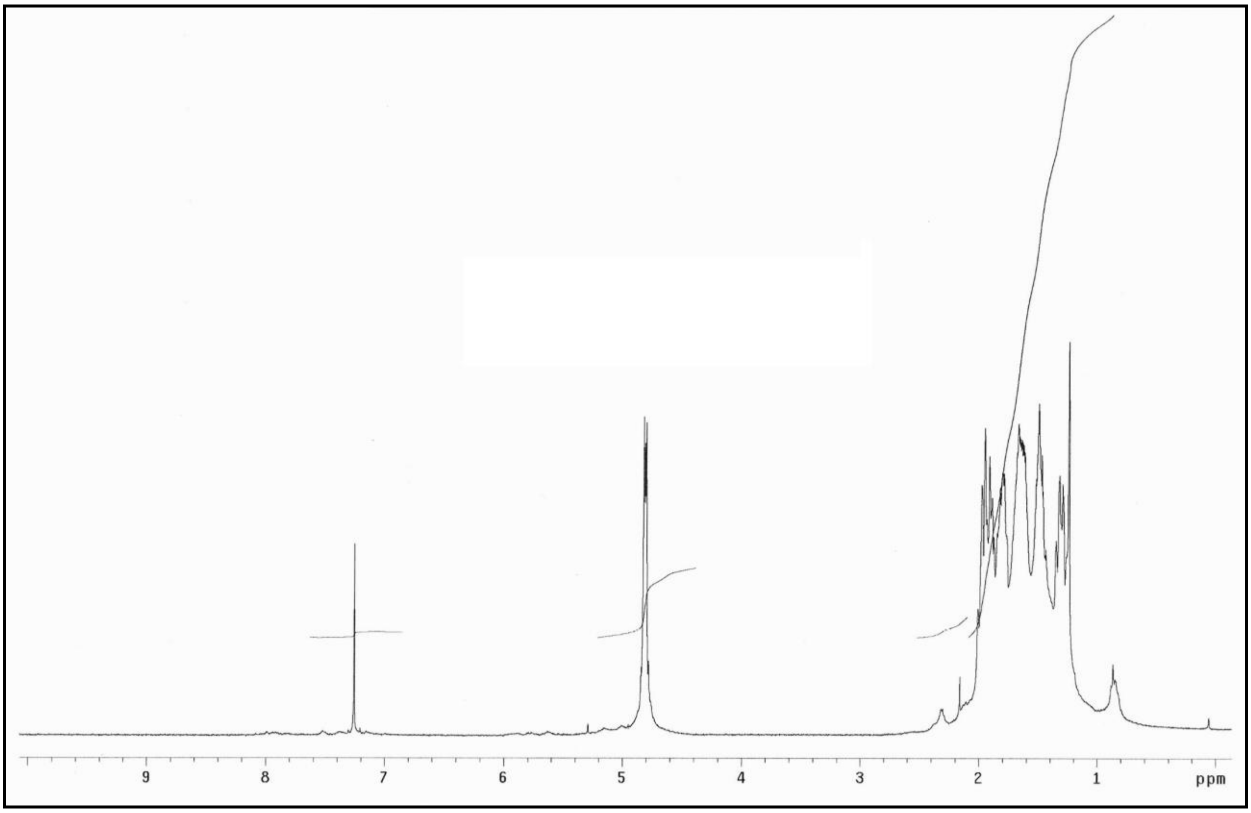

^{1}H NMR spectrum of hexahydro-4*H*-cyclohepta[*d*][1,3]dioxol-2-one (**8b**)

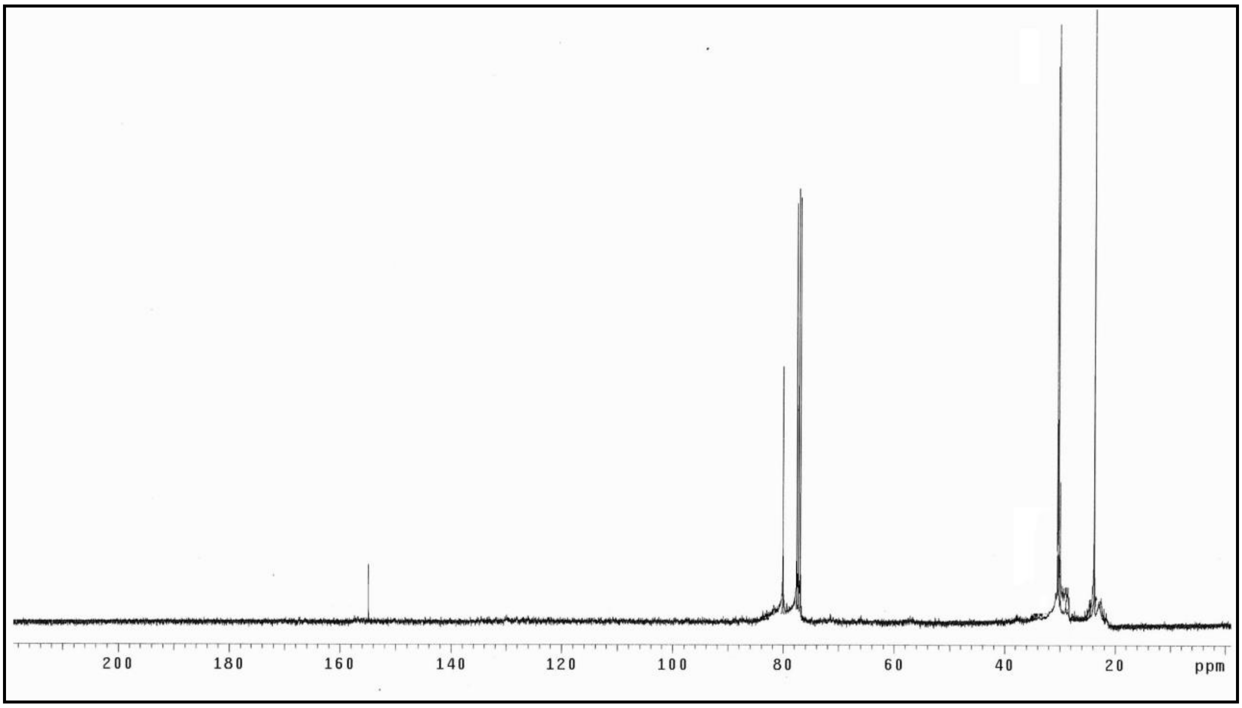

^{13}C NMR spectrum of hexahydro-4*H*-cyclohepta[*d*][1,3]dioxol-2-one (**8b**)

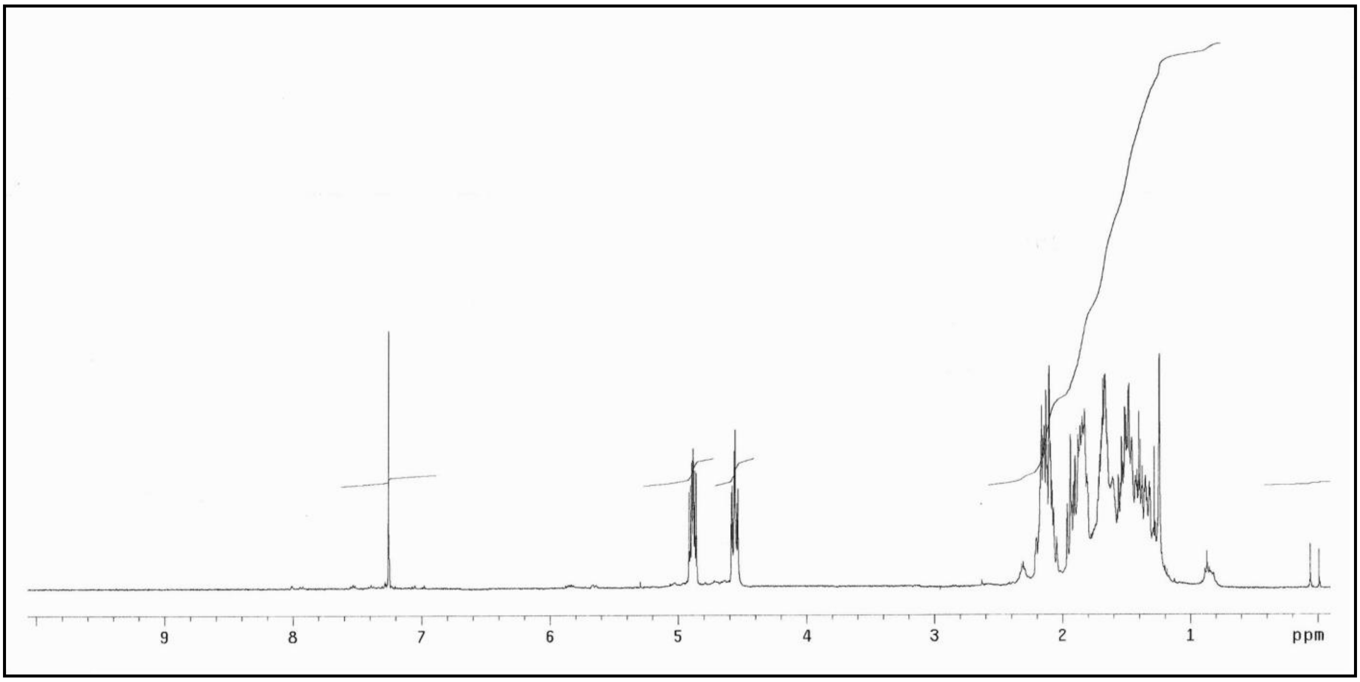

^{1}H NMR spectrum of octahydro-2*H*-cyclohepta[*d*]oxazol-2-one (**9b**)

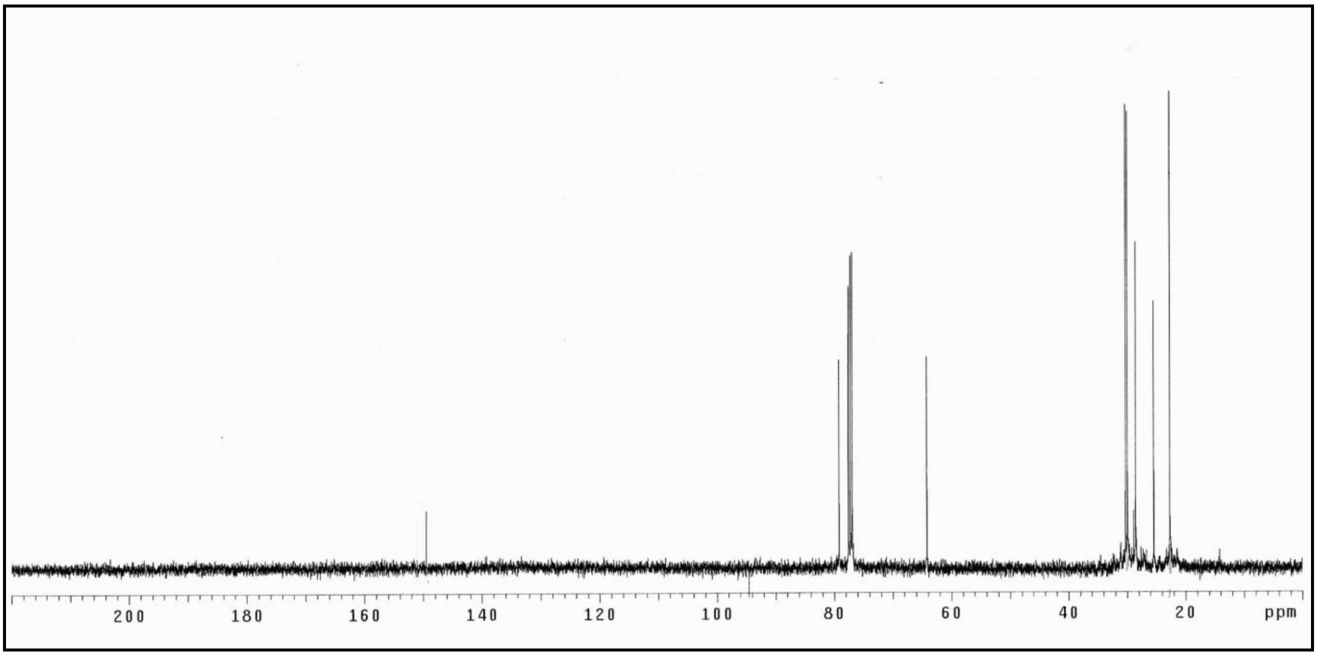

^{13}C NMR spectrum of octahydro-2*H*-cyclohepta[*d*]oxazol-2-one (**9b**)

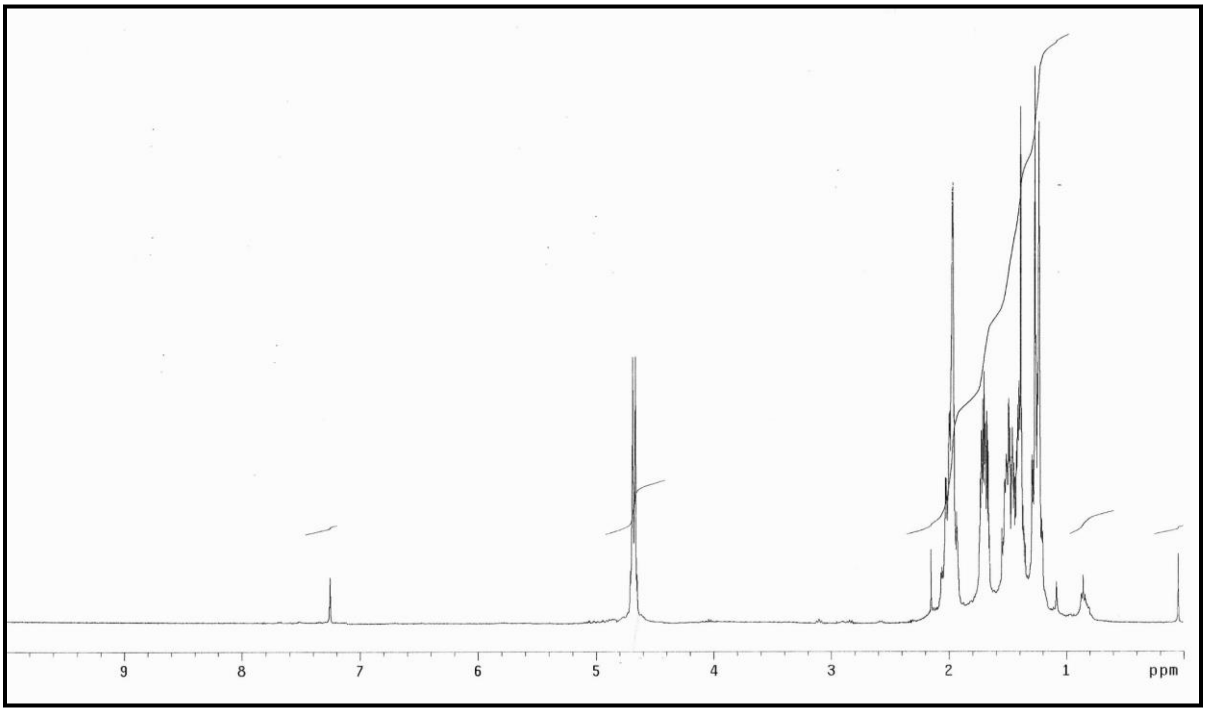

^{1}H NMR spectrum of octahydrocycloocta[*d*][1,3]dioxol-2-one (**8c**)

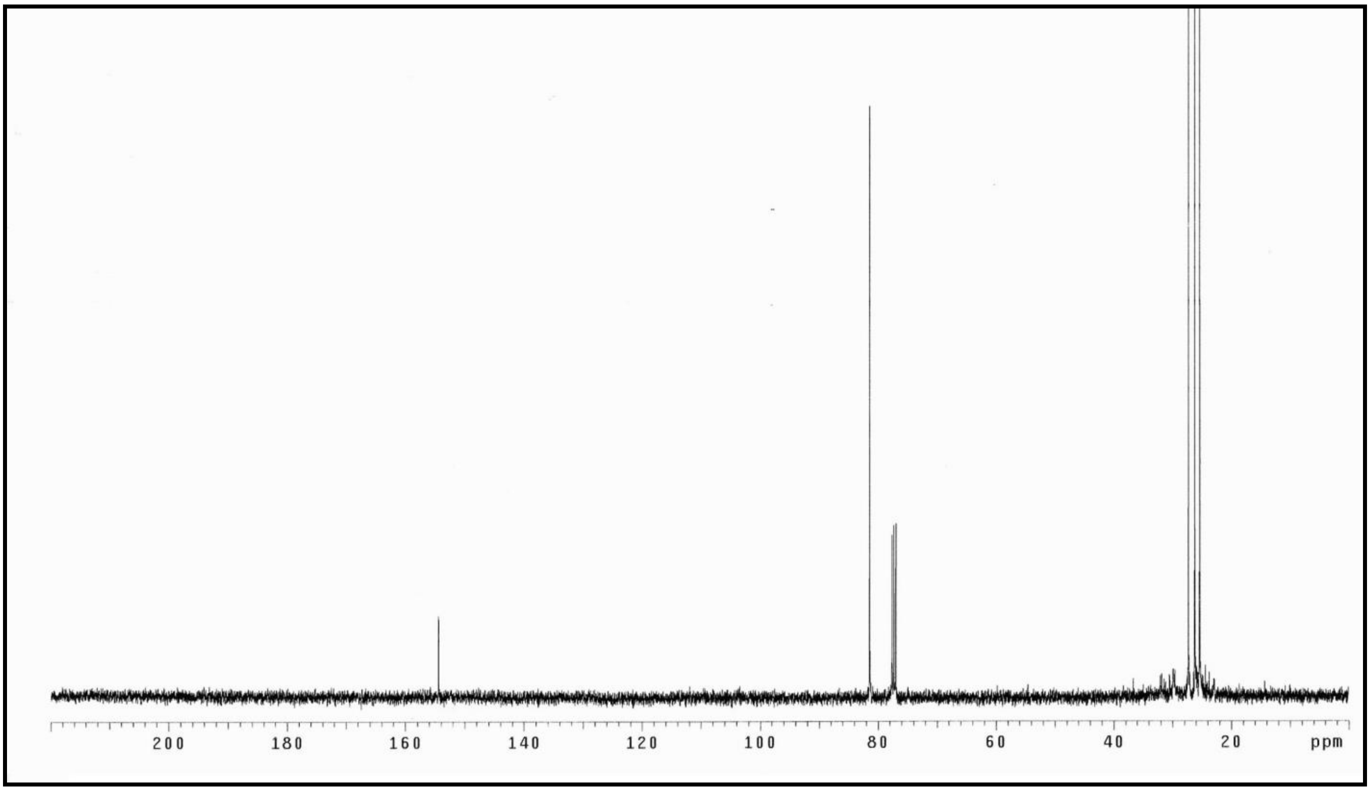

^{13}C NMR spectrum of octahydrocycloocta[*d*][1,3]dioxol-2-one (**8c**)

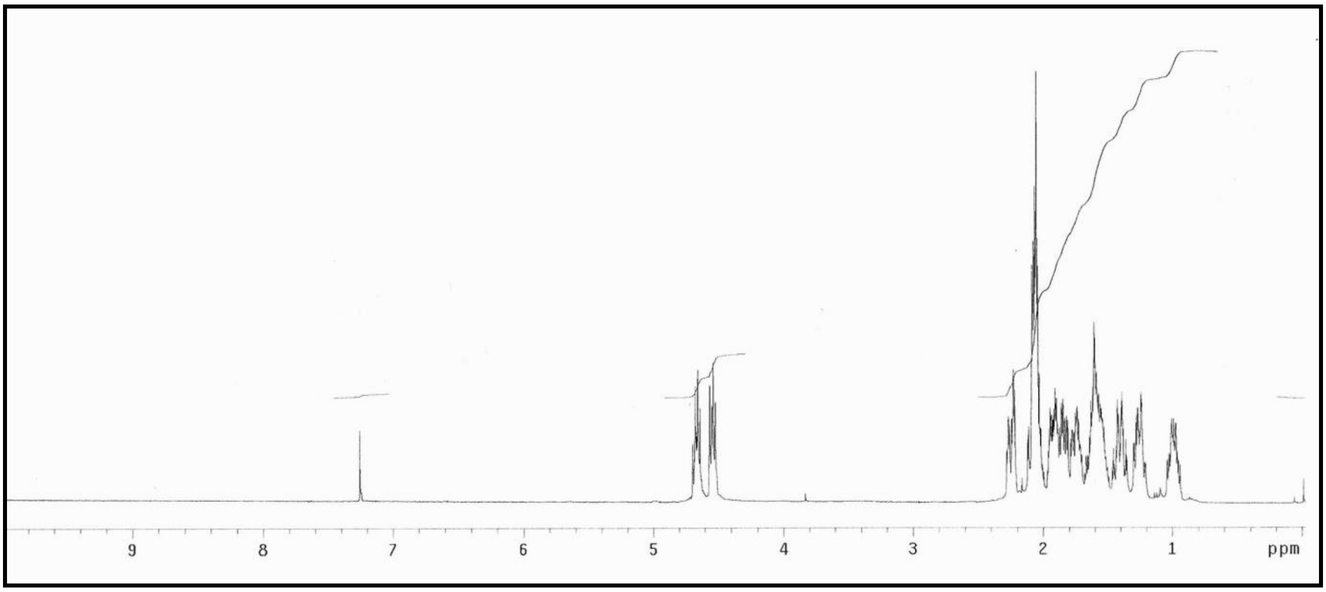

^{1}H NMR spectrum of octahydrocycloocta[*d*]oxazol-2(3*H*)-one (**9c**)

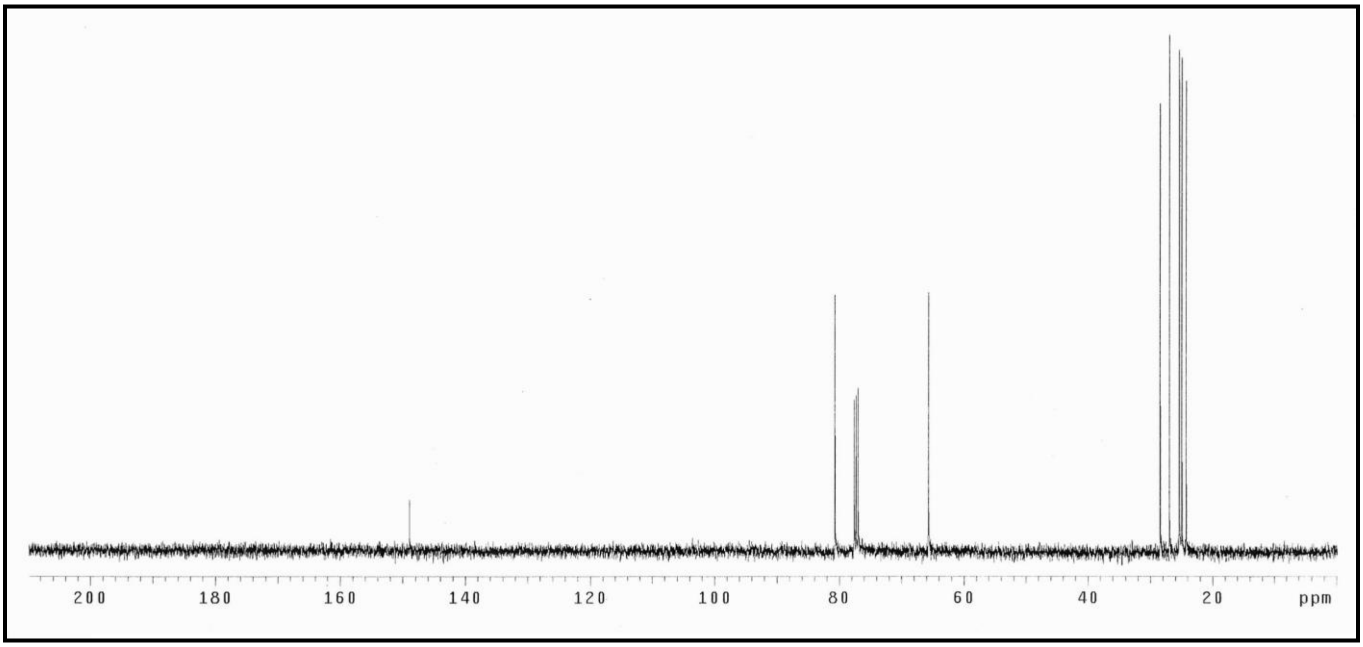

^{13}C NMR spectrum of octahydrocycloocta[*d*]oxazol-2(3*H*)-one (**9c**)

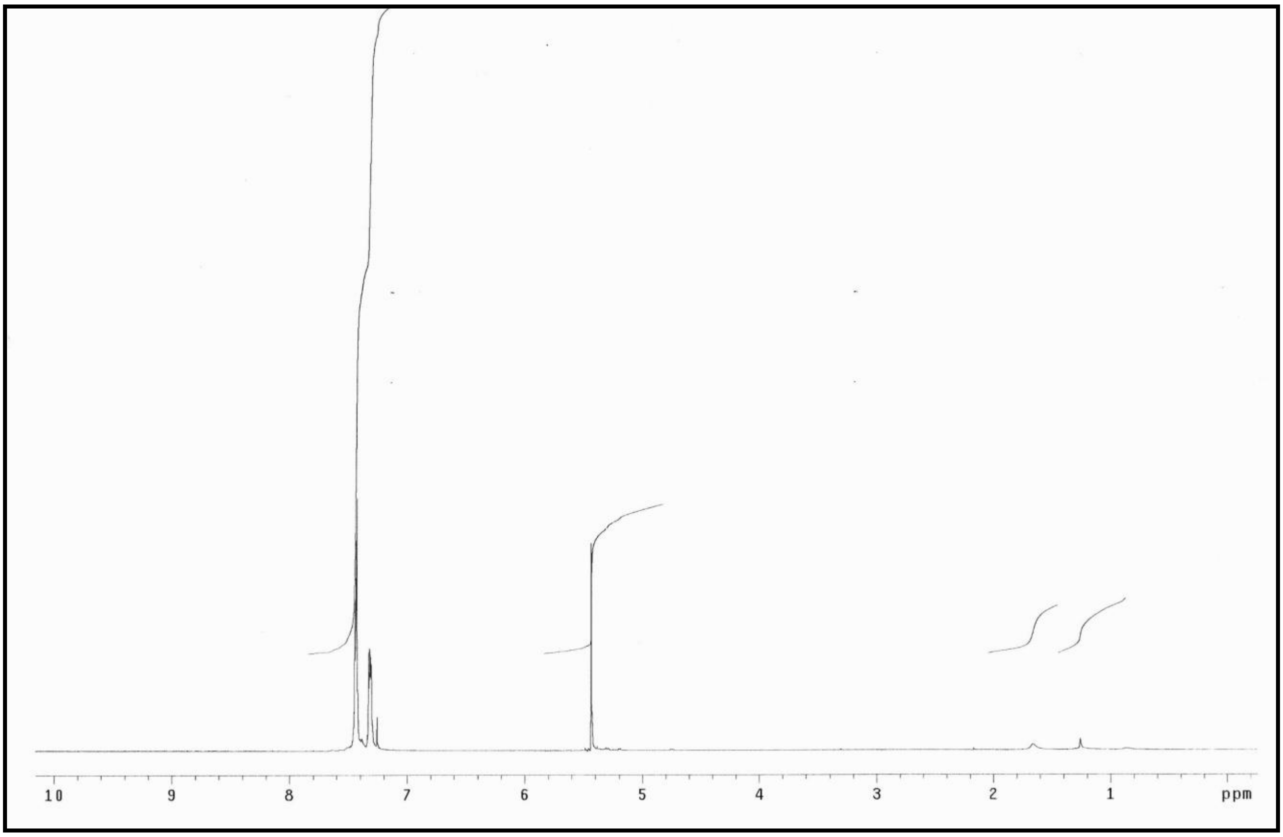

^{1}H NMR spectrum of *trans*-4,5-diphenyl-1,3-dioxolan-2-one (**8d**)

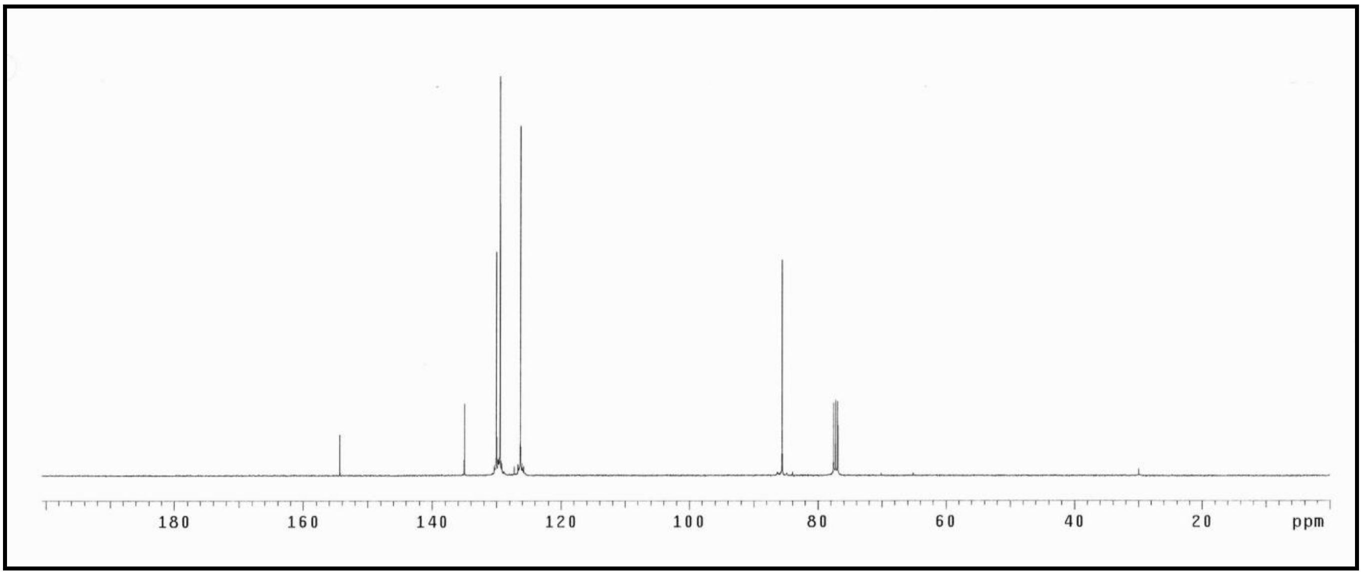

^{13}C NMR spectrum of *trans*-4,5-diphenyl-1,3-dioxolan-2-one (**8d**)

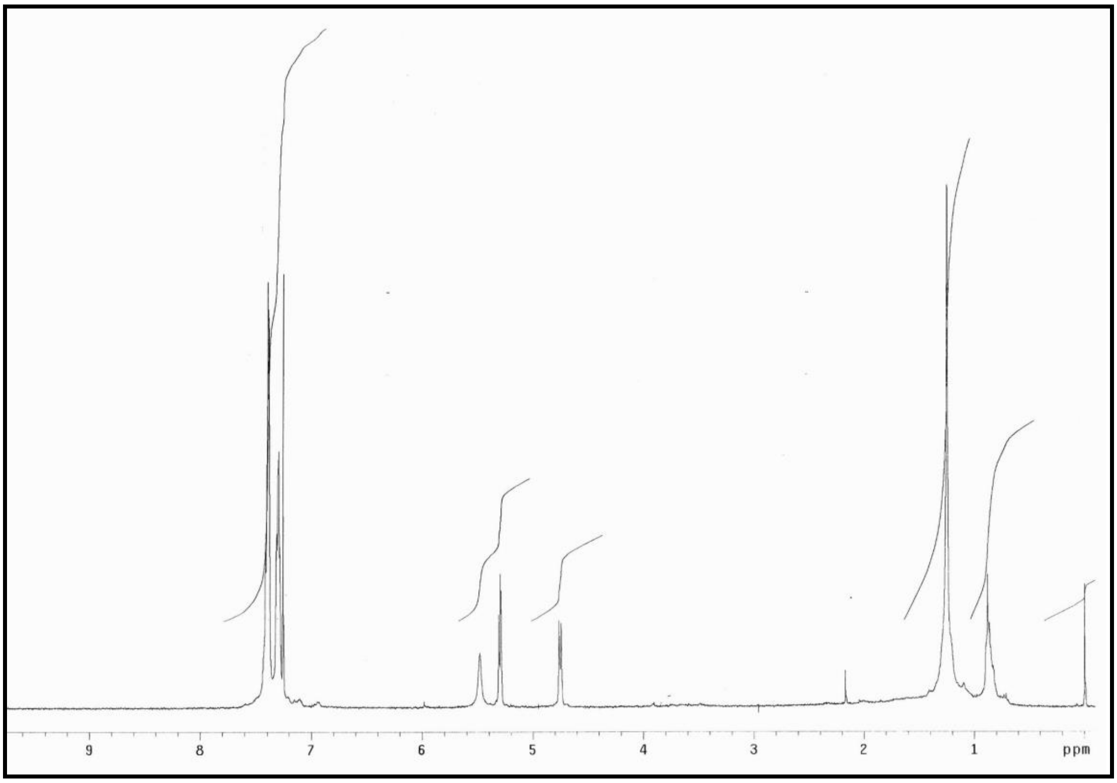

^{1}H NMR spectrum of *trans*-4,5-diphenyloxazolidin-2-one (**9d**)

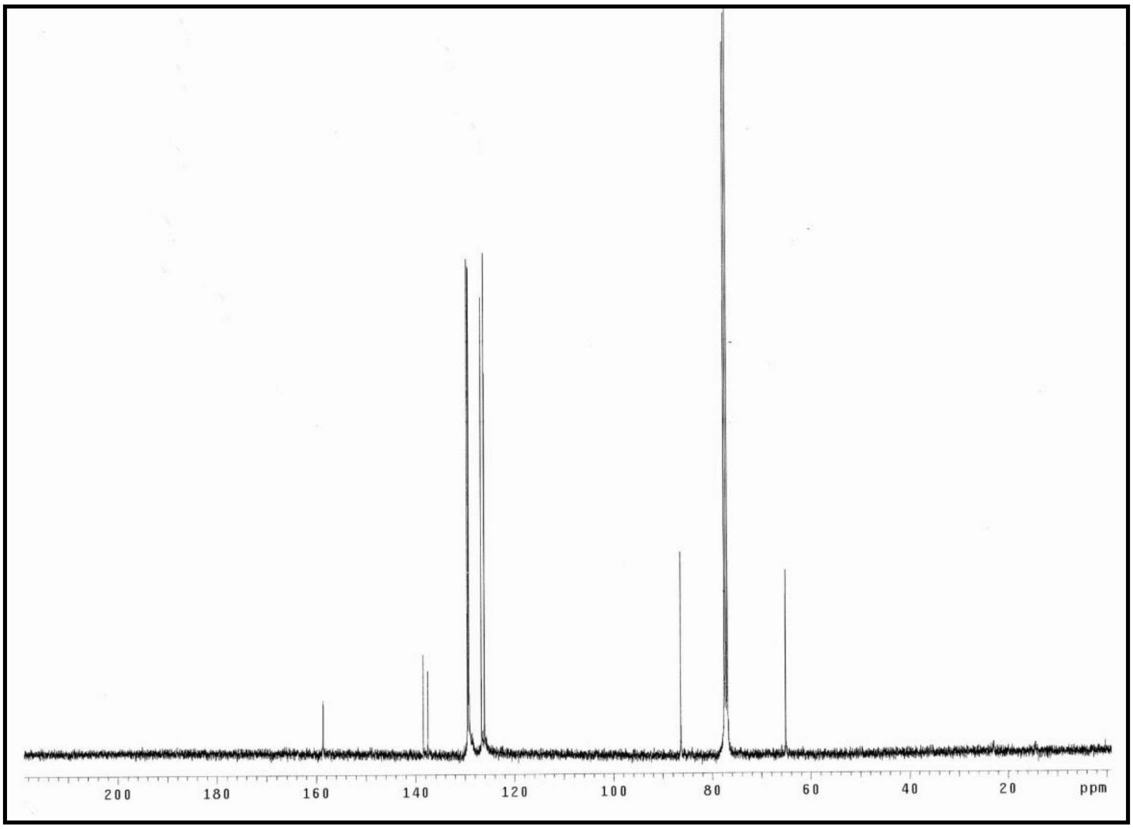

^{13}C NMR spectrum of *trans*-4,5-diphenyloxazolidin-2-one (**9d**)

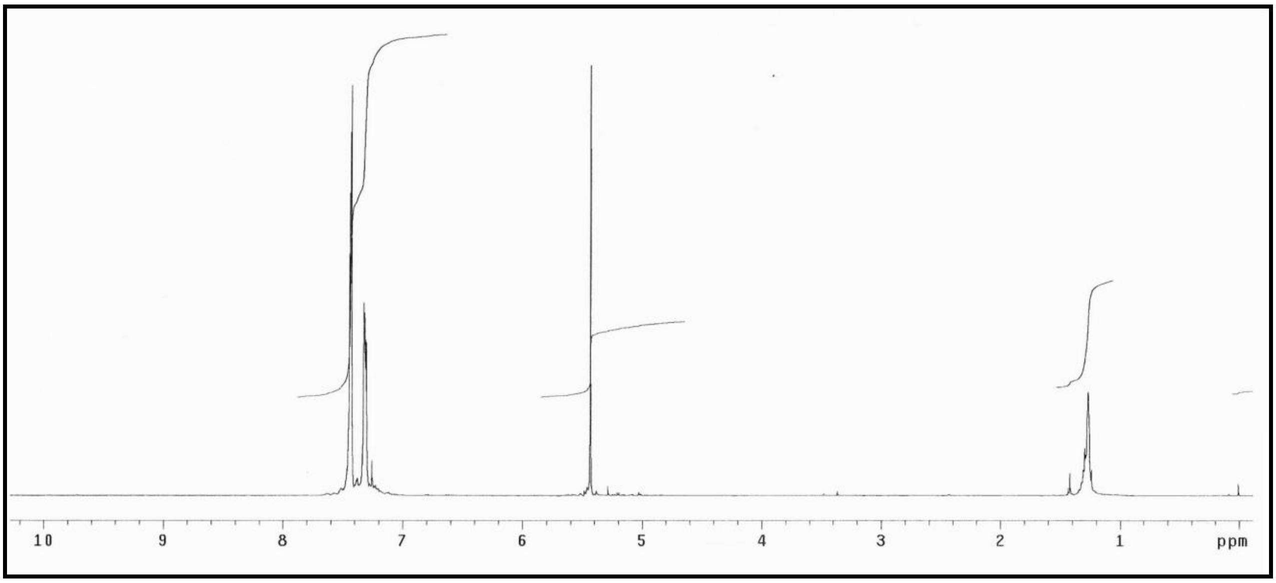

^{1}H NMR spectrum of *cis*-4,5-diphenyl-1,3-dioxolan-2-one (**8e**)

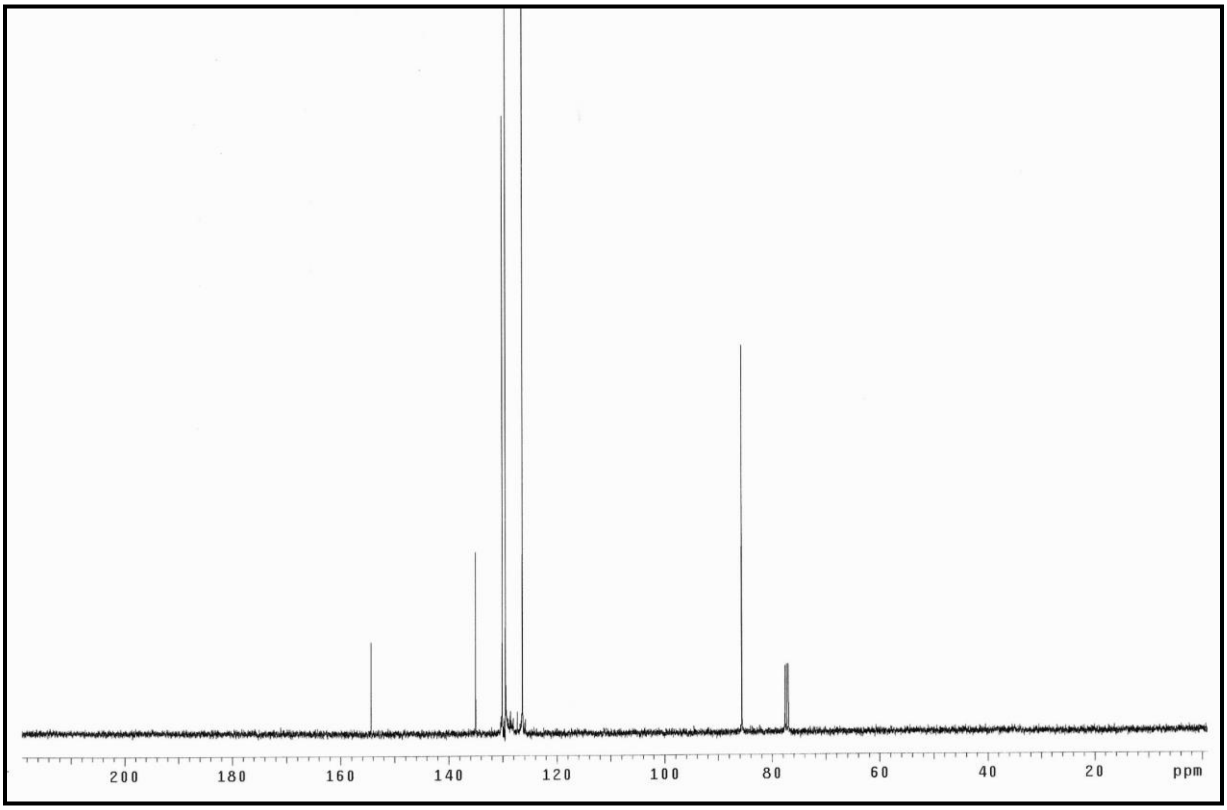

^{13}C NMR spectrum of *cis*-4,5-diphenyl-1,3-dioxolan-2-one (**8e**)

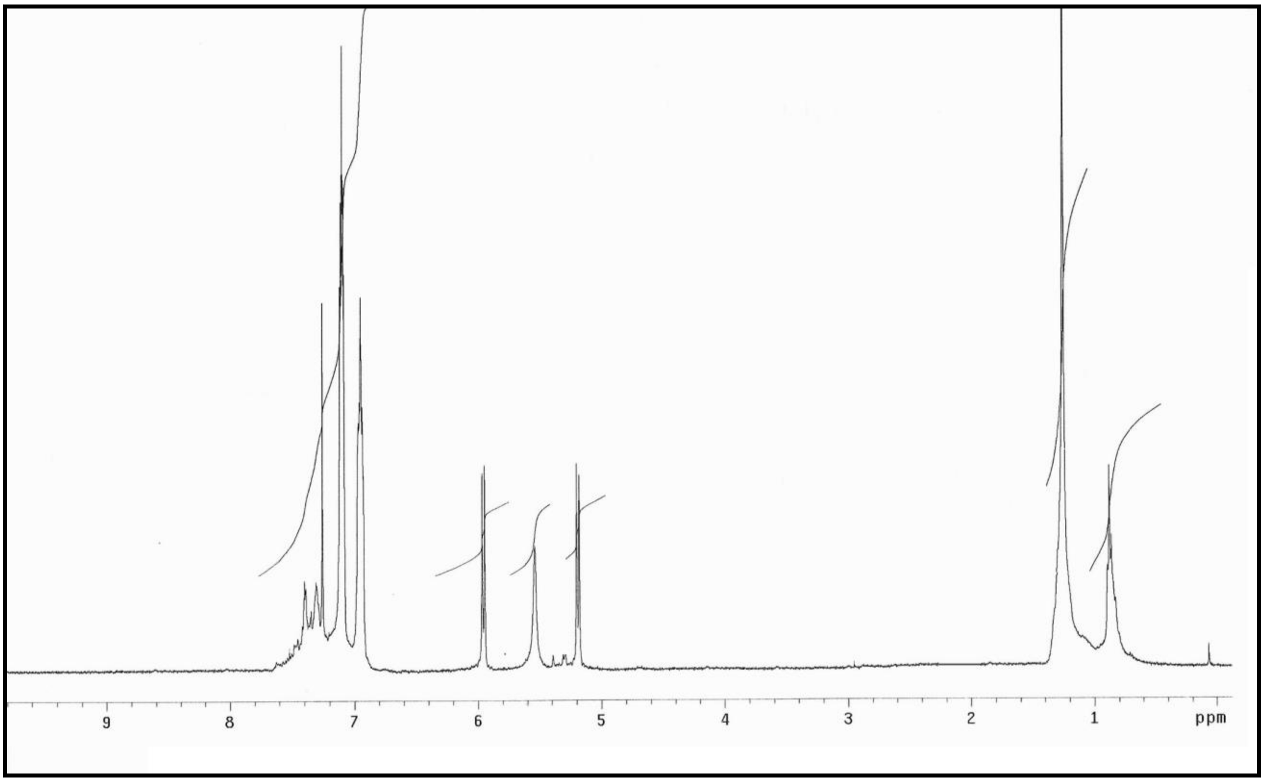

^{1}H NMR spectrum of *cis*-4,5-diphenyloxazolidin-2-one (**9e**)

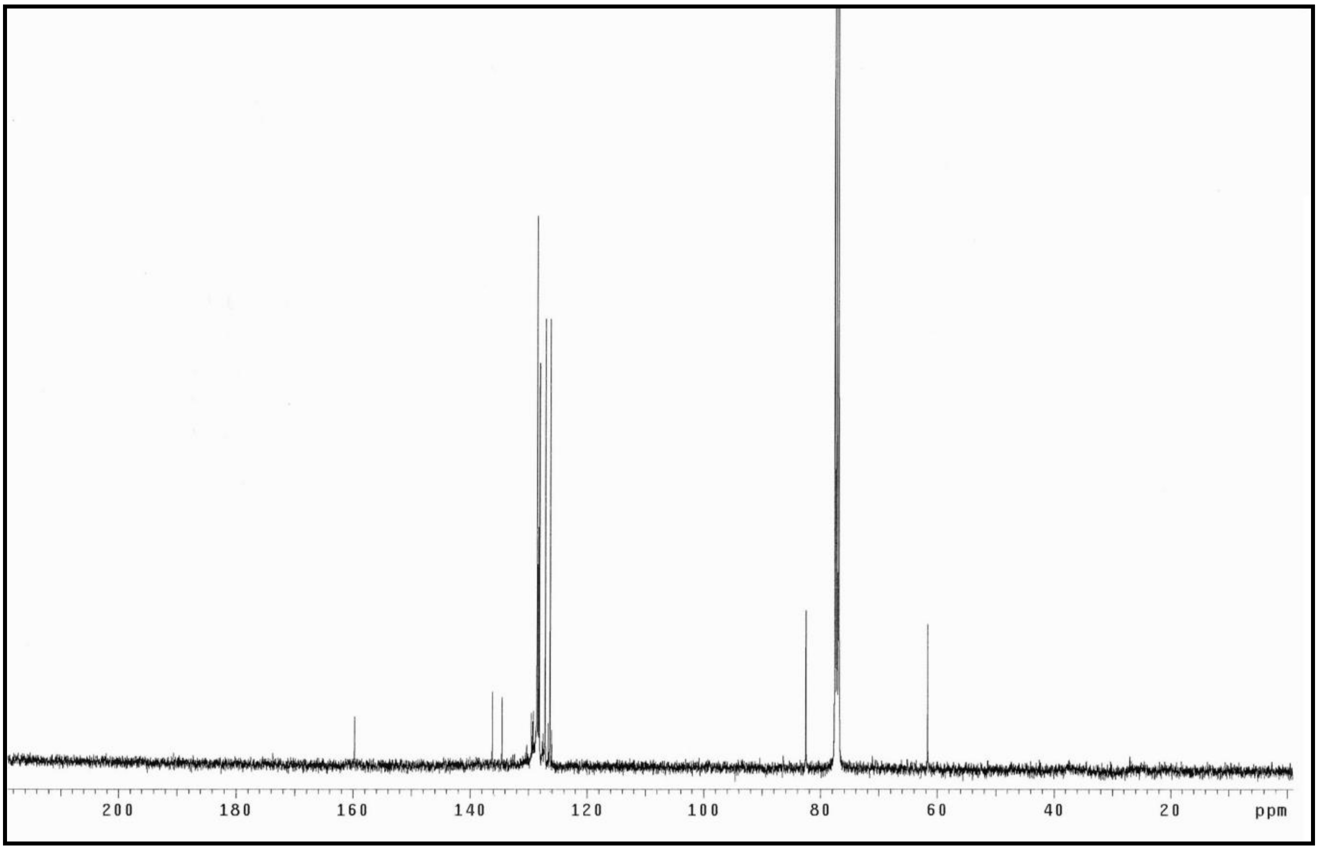

^{13}C NMR spectrum of *cis*-4,5-diphenyloxazolidin-2-one (**9e**)

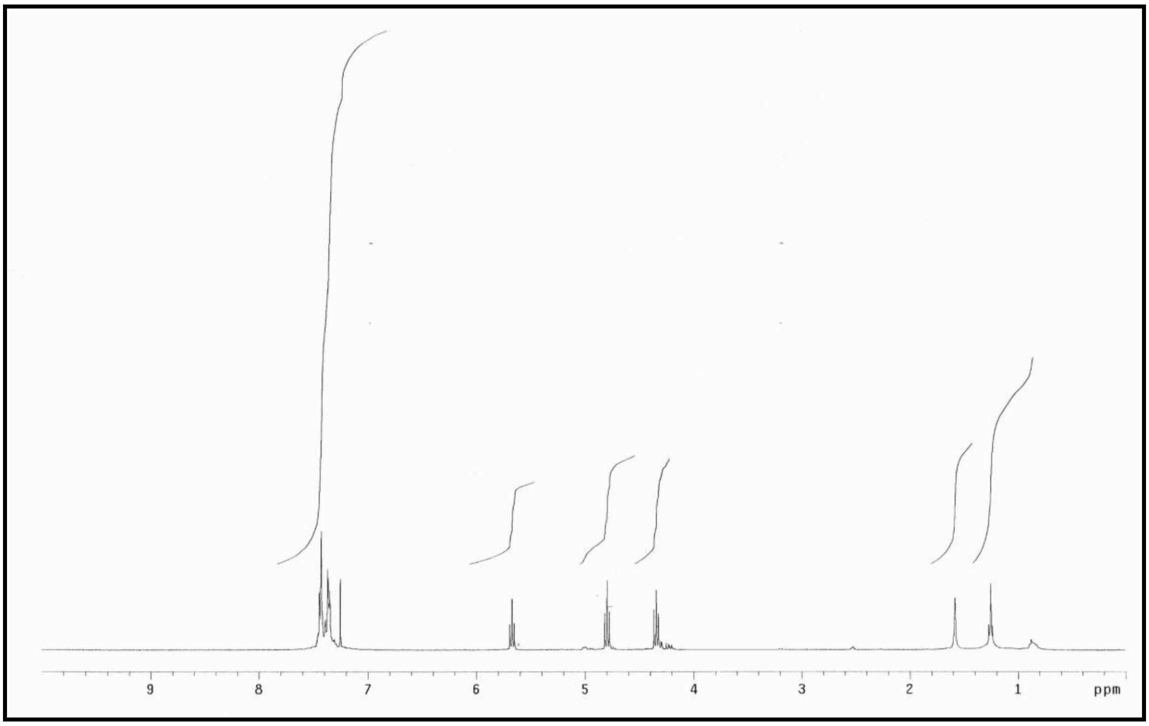

^{1}H NMR spectrum of 4-phenyl-1,3-dioxolan-2-one (**8f**)

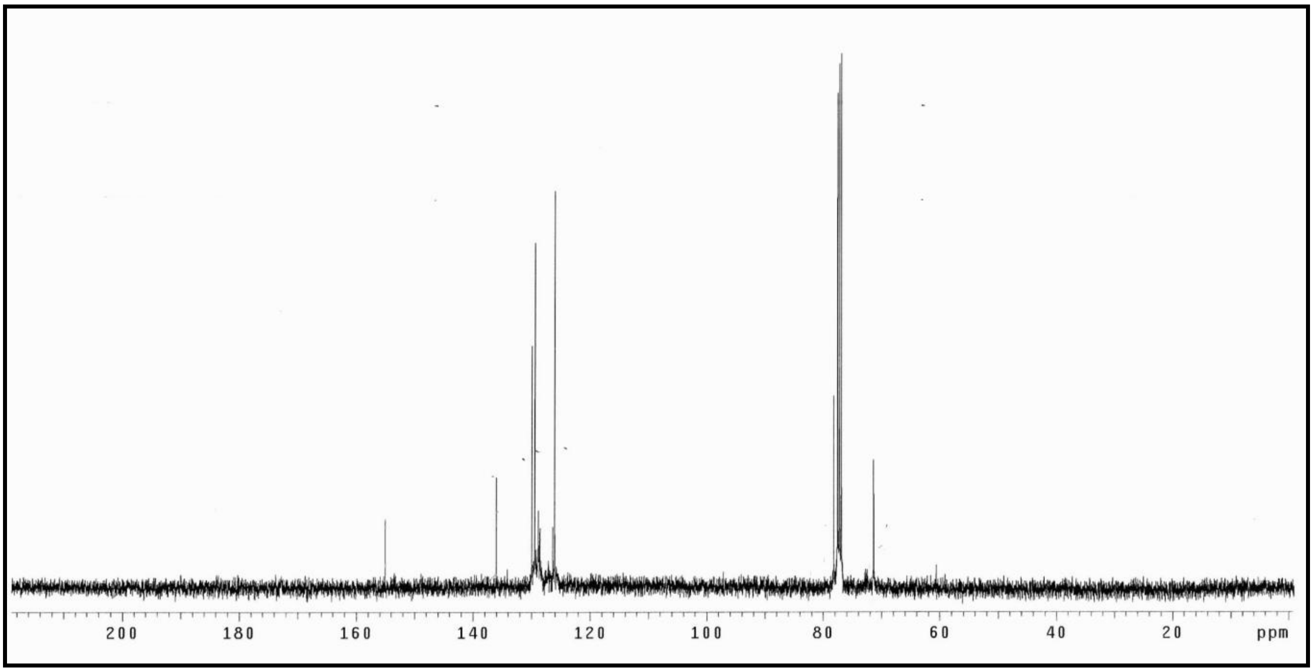

^{13}C NMR spectrum of 4-phenyl-1,3-dioxolan-2-one (**8f**)

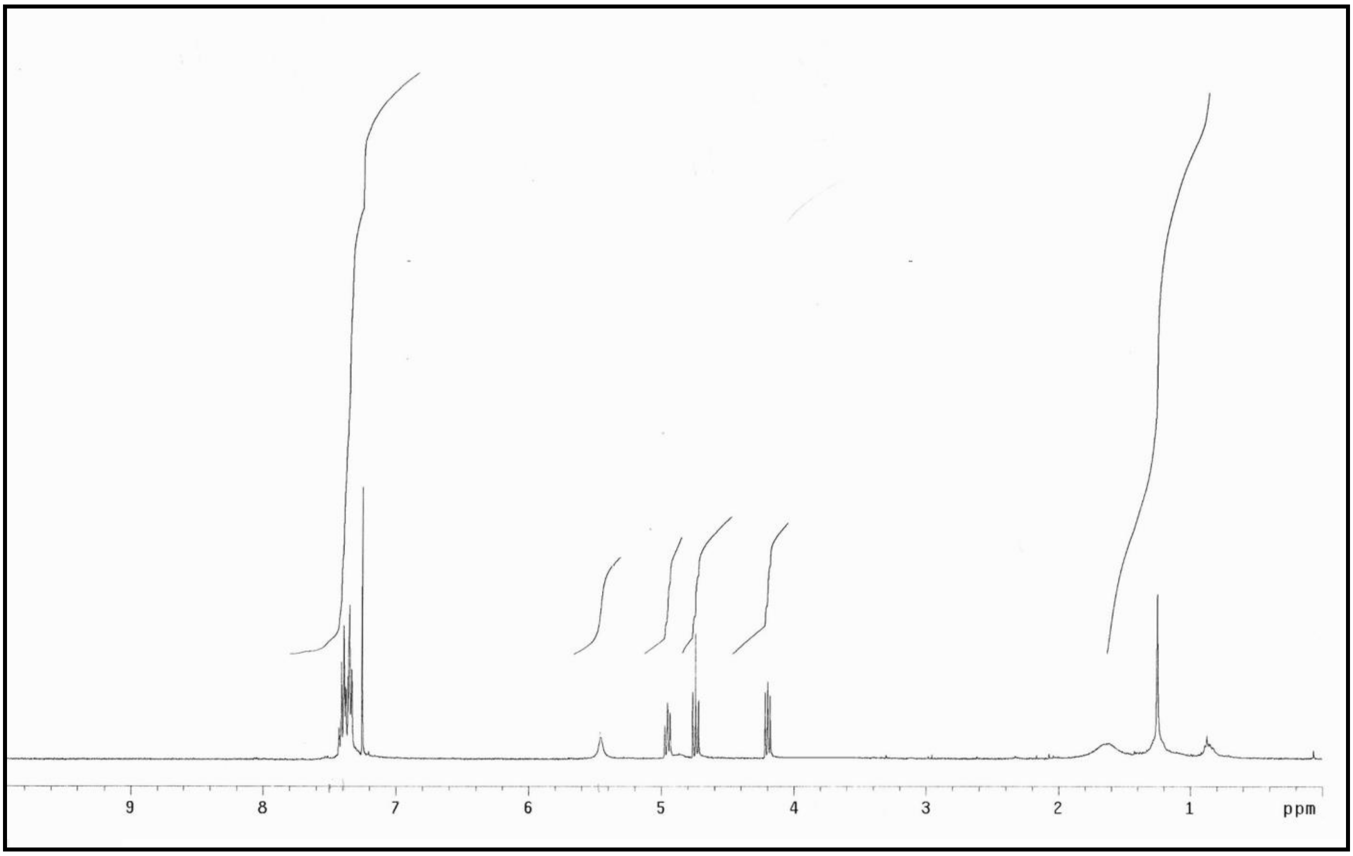

^{1}H NMR spectrum of 4-phenyloxazolidin-2-one (**9f**)

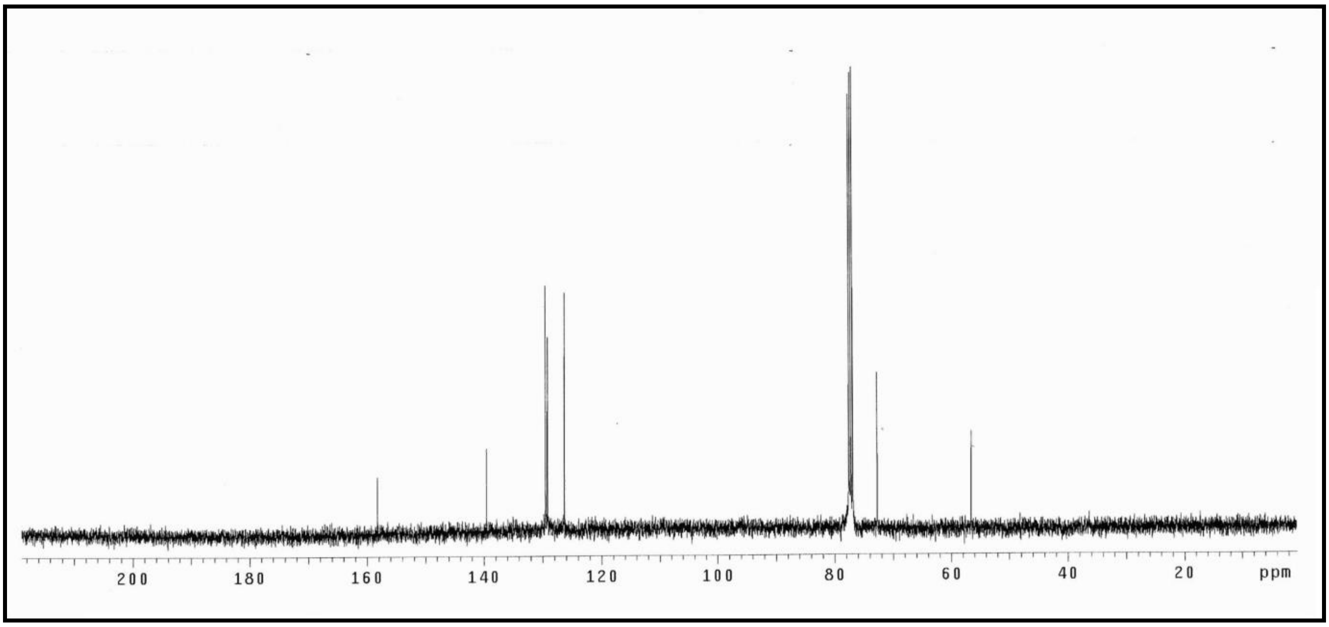

^{13}C NMR spectrum of 4-phenyloxazolidin-2-one (**9f**)

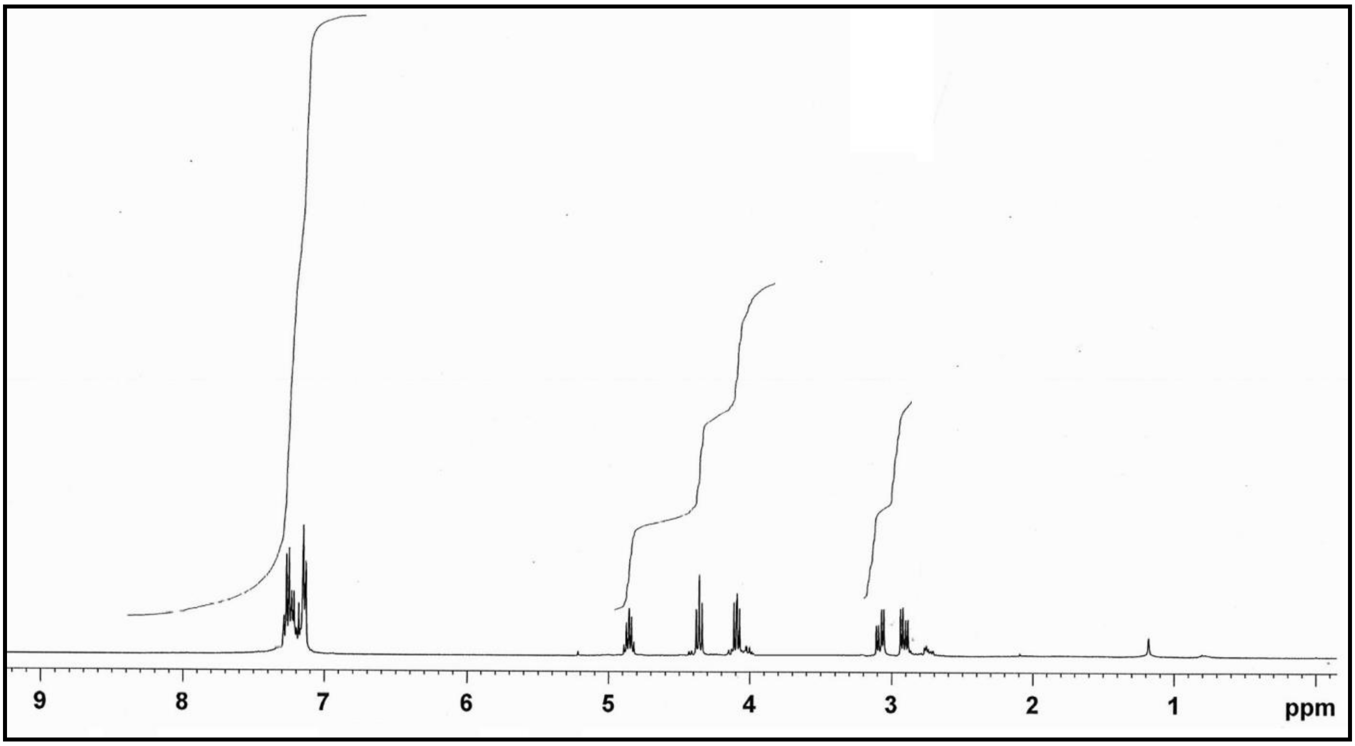

^{1}H NMR spectrum of 4-benzyl-1,3-dioxolan-2-one (**8g**)

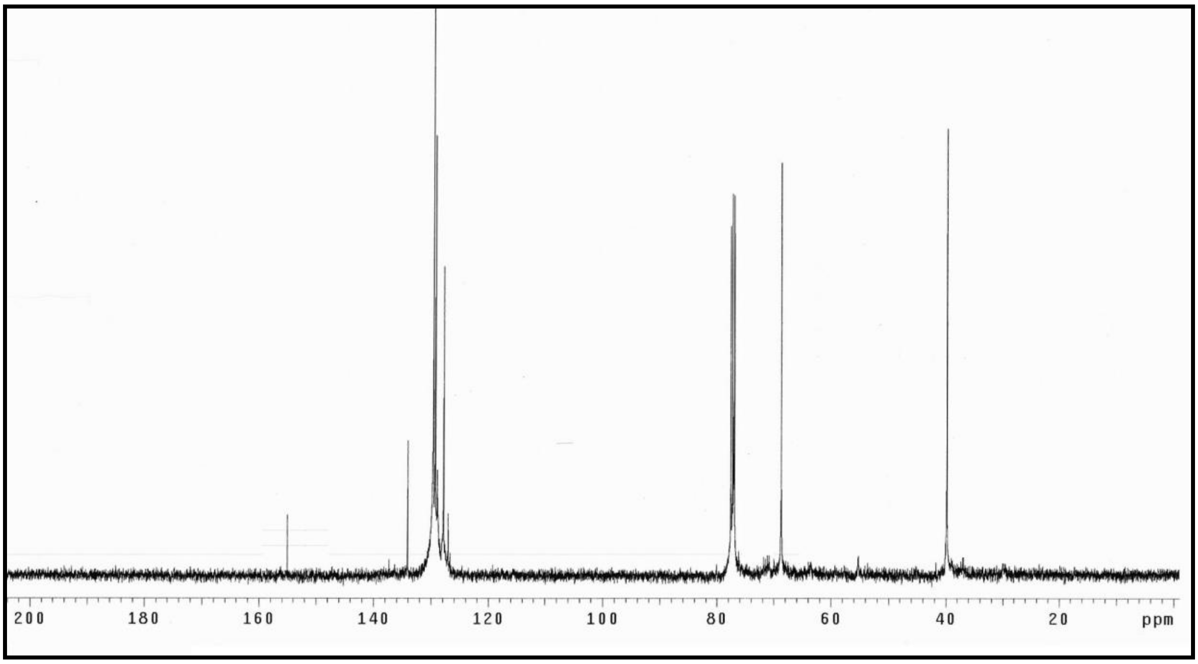

^{13}C NMR spectrum of 4-benzyl-1,3-dioxolan-2-one (**8g**)

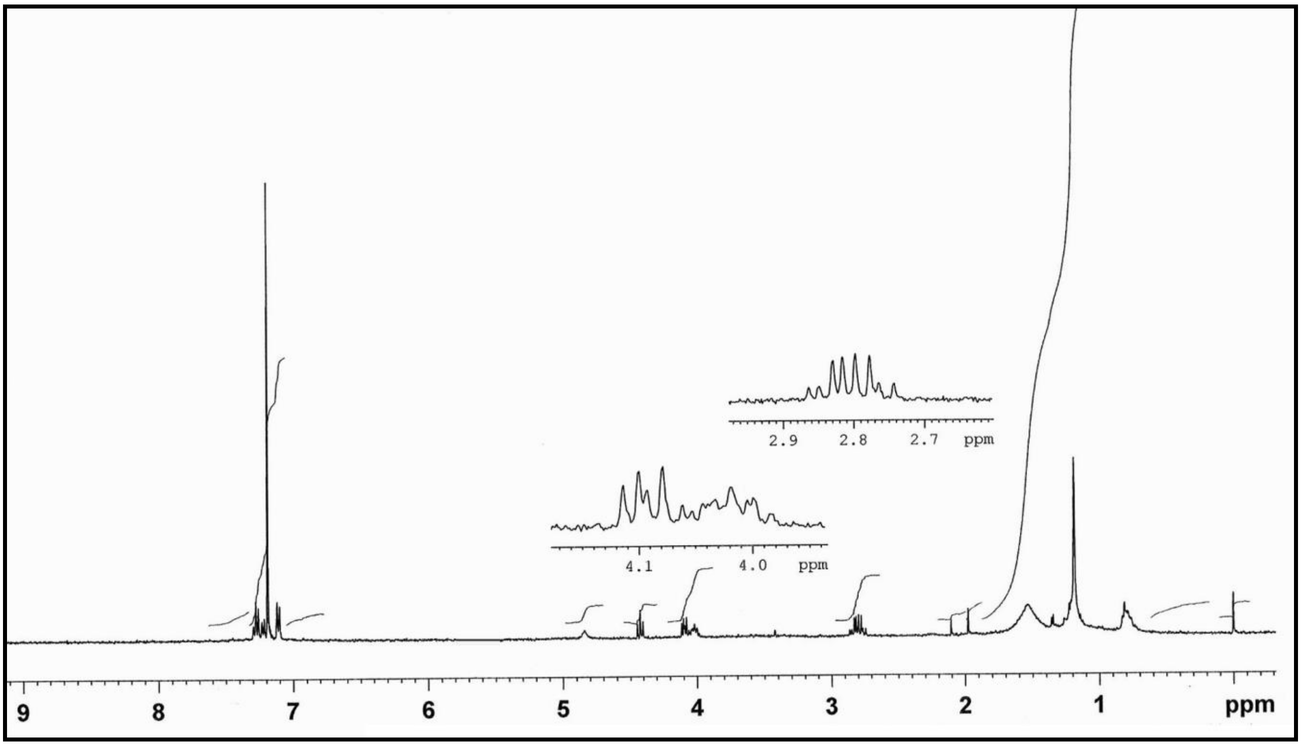

^{1}H NMR spectrum of 4-benzyloxazolidin-2-one (**9g**)

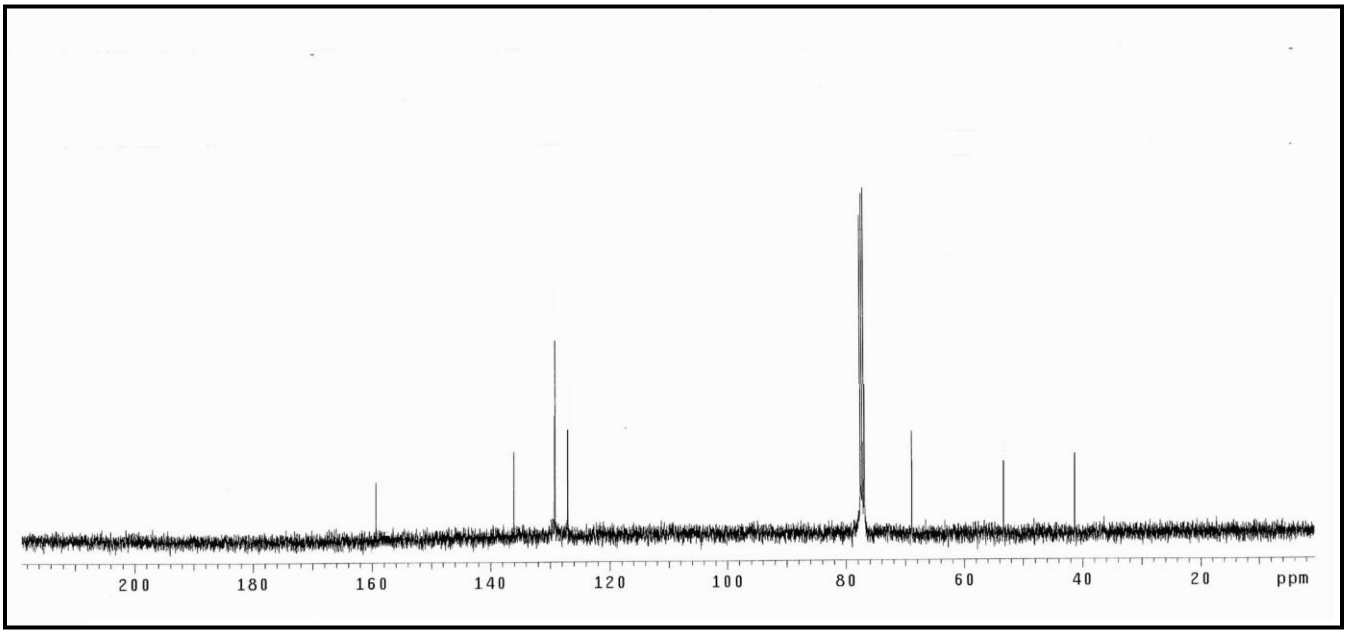

^{13}C NMR spectrum of 4-benzyloxazolidin-2-one (**9g**)

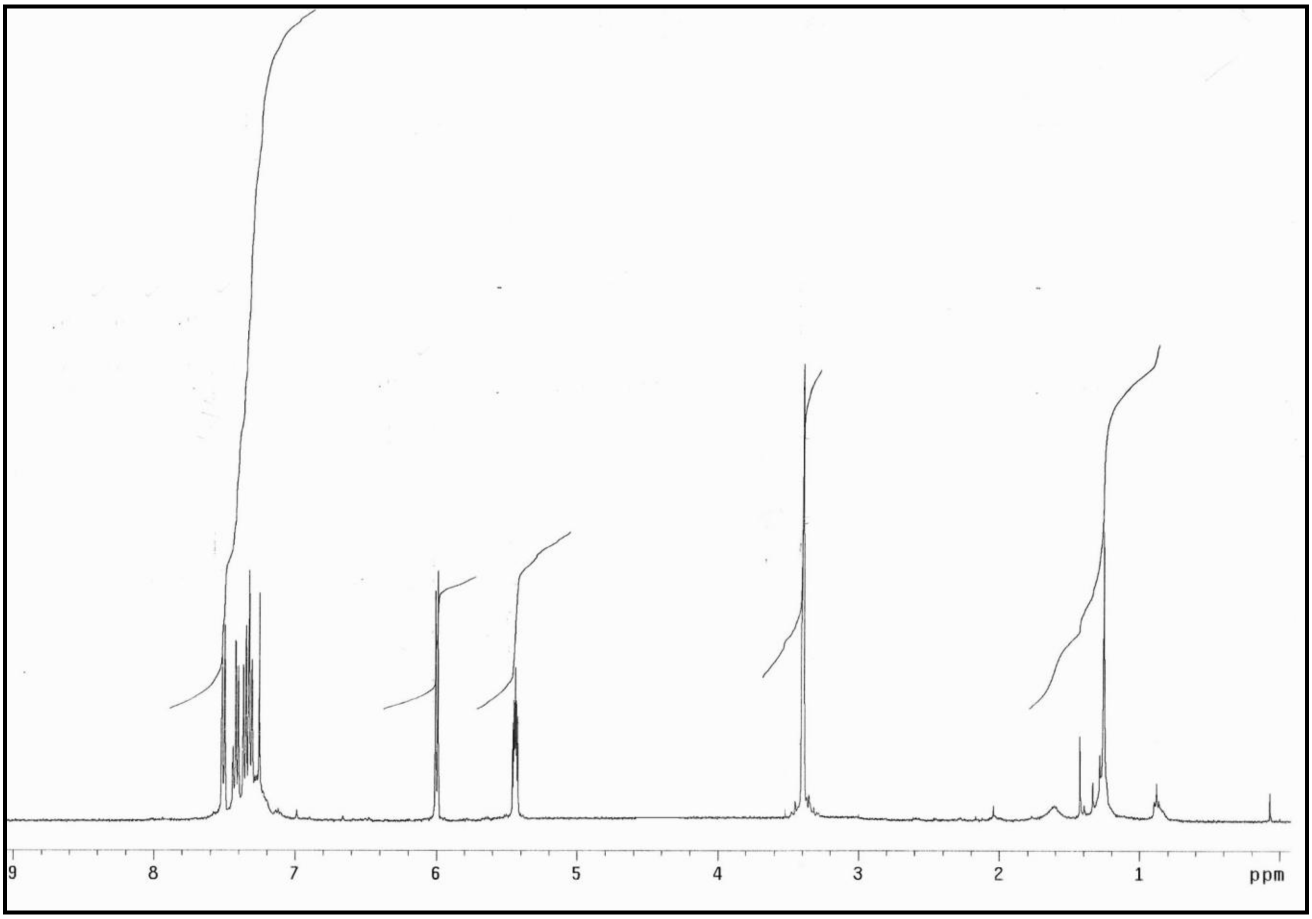

^{1}H NMR spectrum of 8,8a-dihydro-3a*H*-indeno[1,2-*d*][1,3]dioxol-2-one (**8h**)

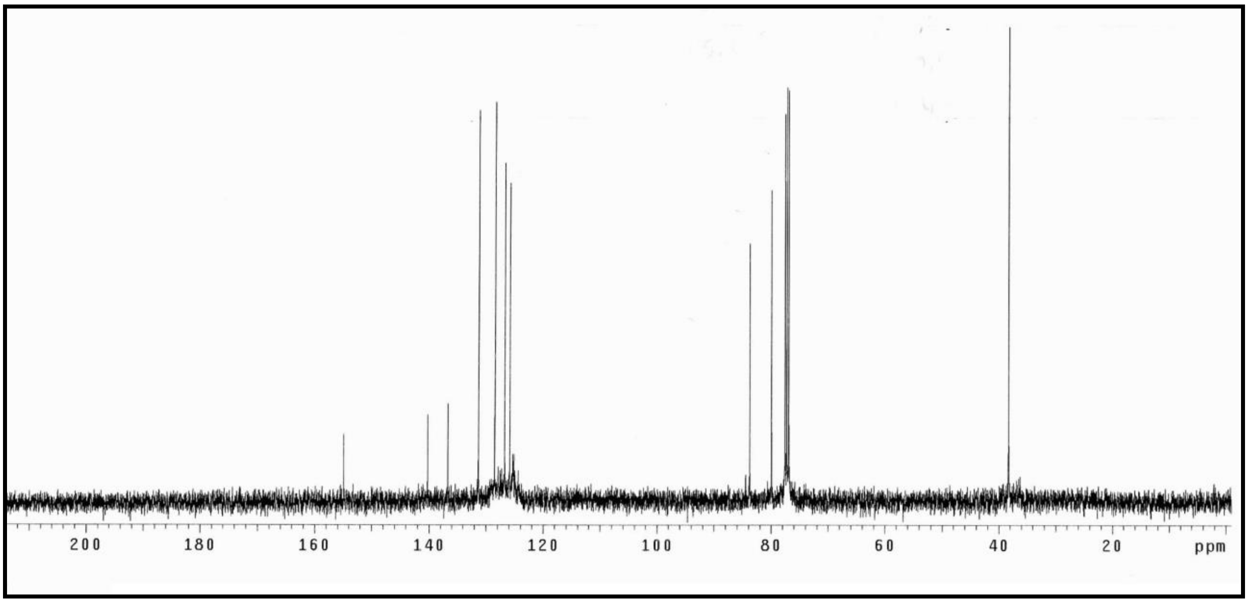

^{13}C NMR spectrum of 8,8a-dihydro-3a*H*-indeno[1,2-*d*][1,3]dioxol-2-one (**8h**)

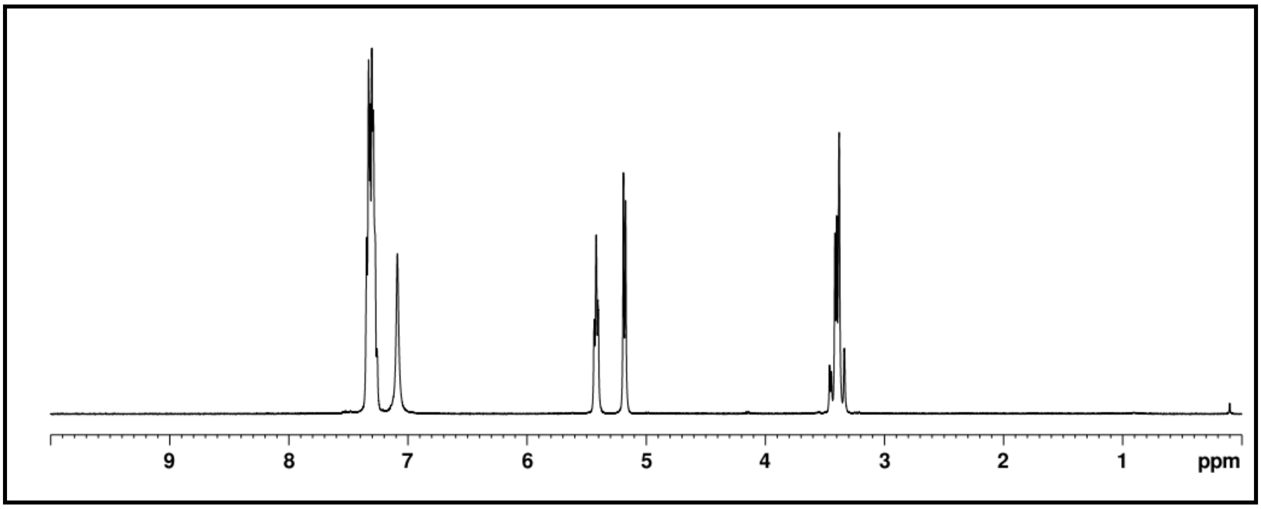

^{1}H NMR spectrum of 3,3a,8,8a-tetrahydro-2*H*-indeno[1,2-*d*]oxazol-2-one (**9h**)

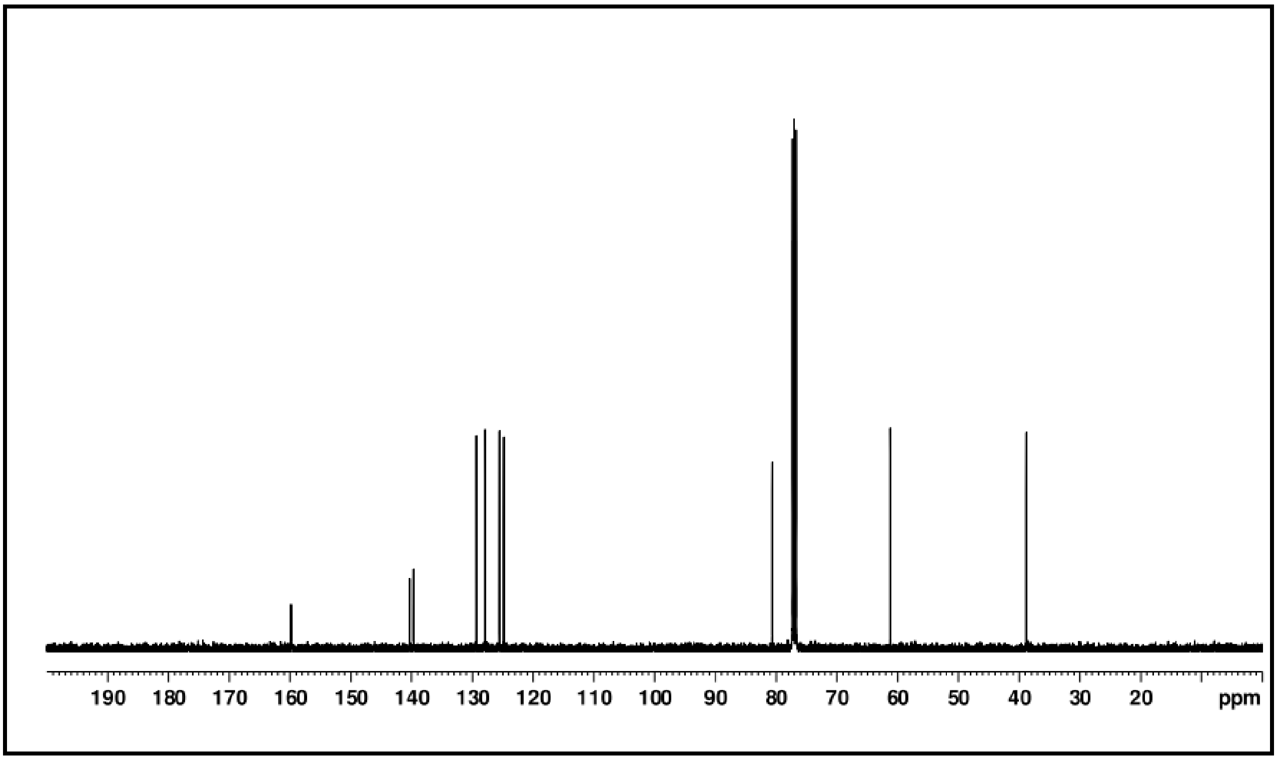

^{13}C NMR spectrum of 3,3a,8,8a-tetrahydro-2*H*-indeno[1,2-*d*]oxazol-2-one (**9h**)

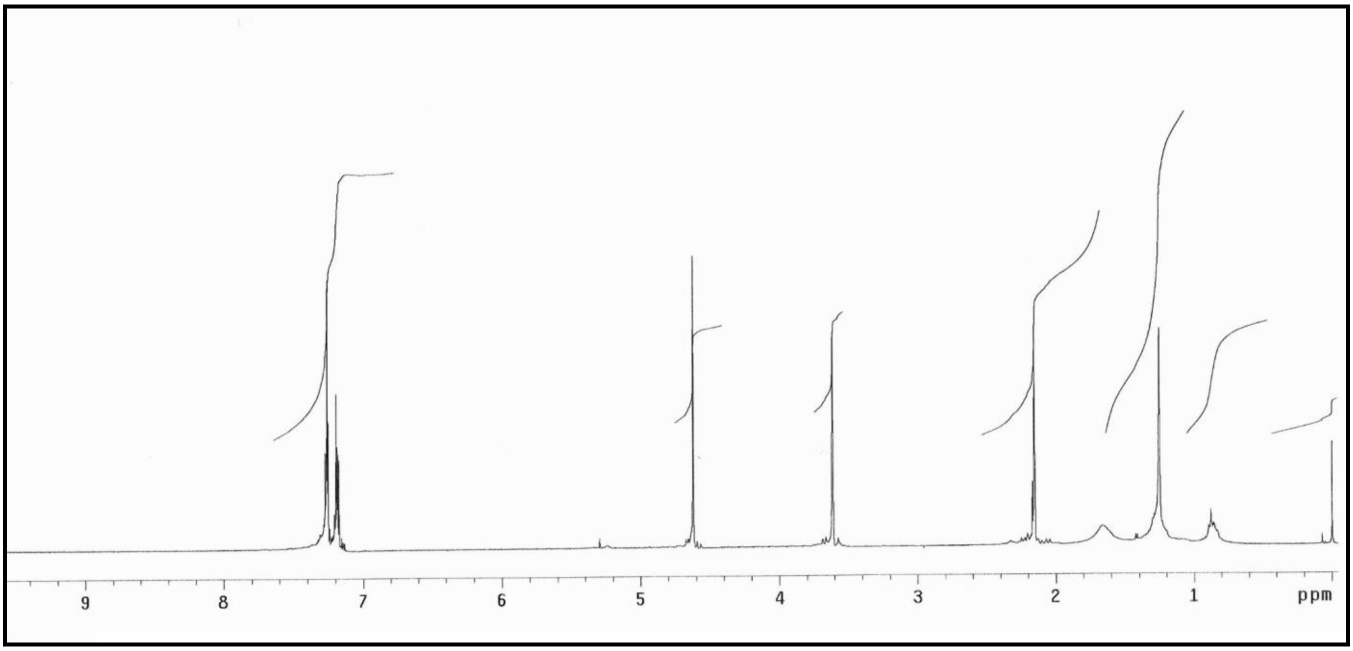

^{1}H NMR spectrum of 3a,4,9,9a-tetrahydro-4,9-methanonaphtho[2,3-*d*][1,3]dioxol-2-one (**8i**)

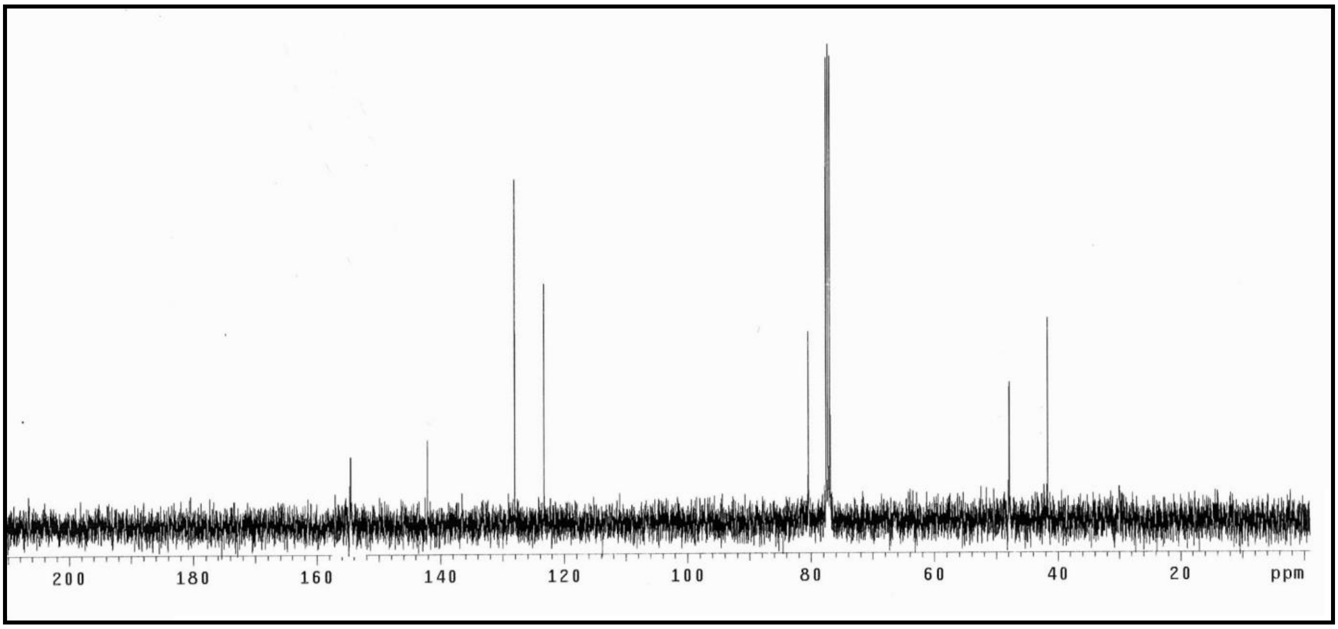

^{13}C NMR spectrum of 3a,4,9,9a-tetrahydro-4,9-methanonaphtho[2,3-*d*][1,3]dioxol-2-one (**8i**)

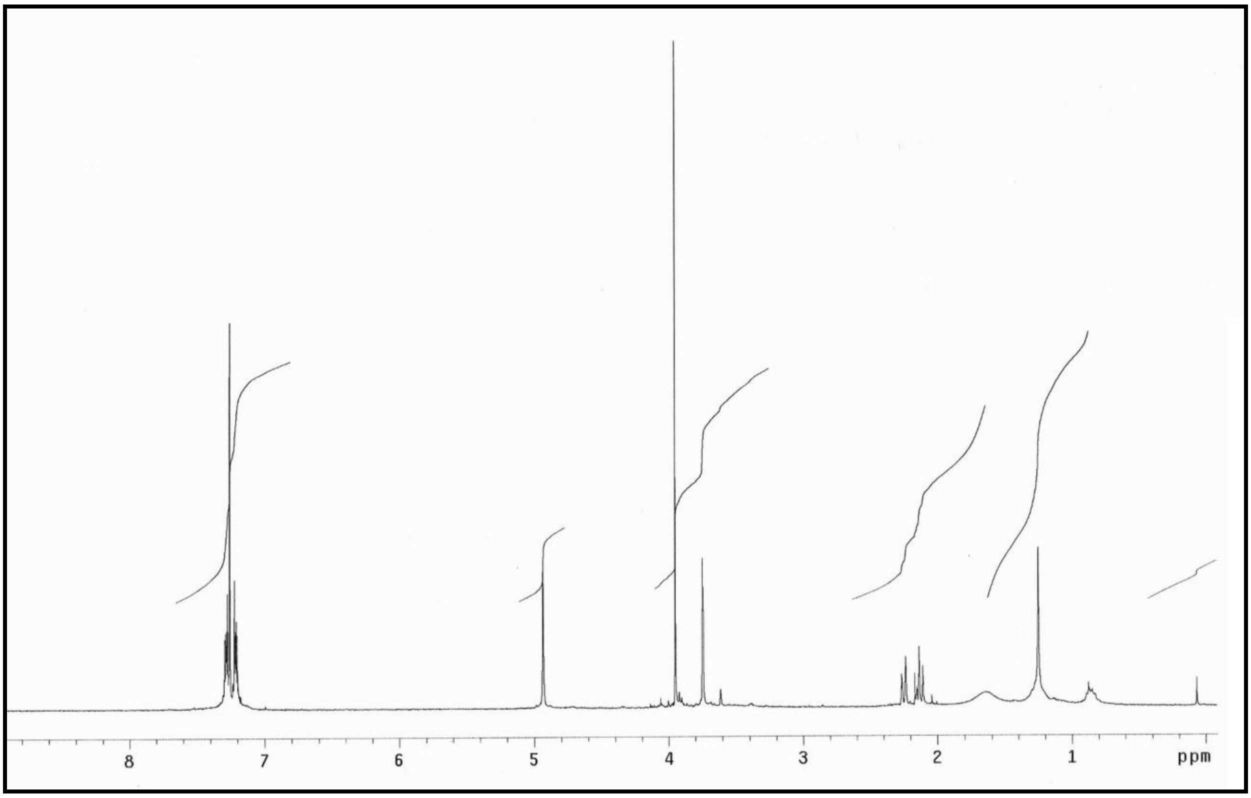

^{1}H NMR spectrum of 3a,4,9,9a-tetrahydro-4,9-methanonaphtho[2,3-*d*]oxazol-2(3*H*)-one (**9i**)

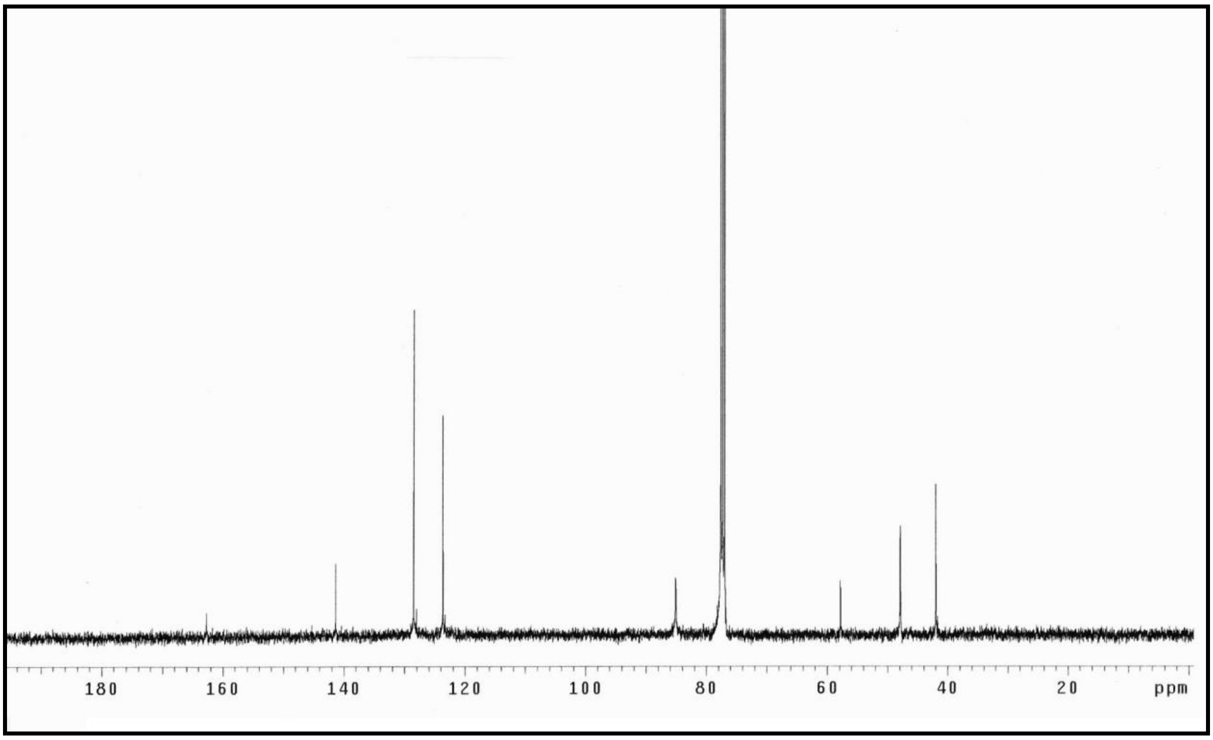

^{13}C NMR spectrum of 3a,4,9,9a-tetrahydro-4,9-methanonaphtho[2,3-*d*]oxazol-2(3*H*)-one (**9i**)

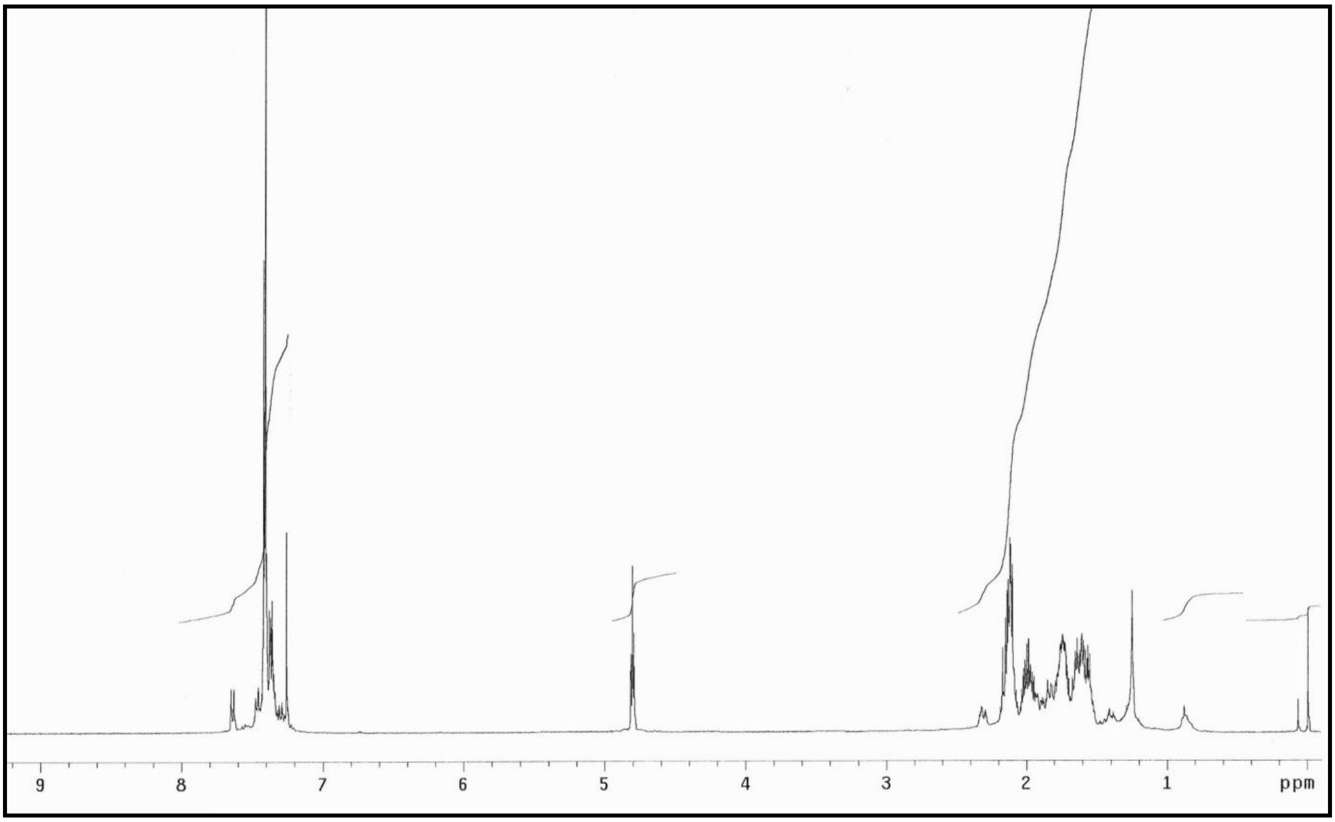

^{1}H NMR spectrum of 3a-phenylhexahydrobenzo[*d*][1,3]dioxol-2-one (**8j**)

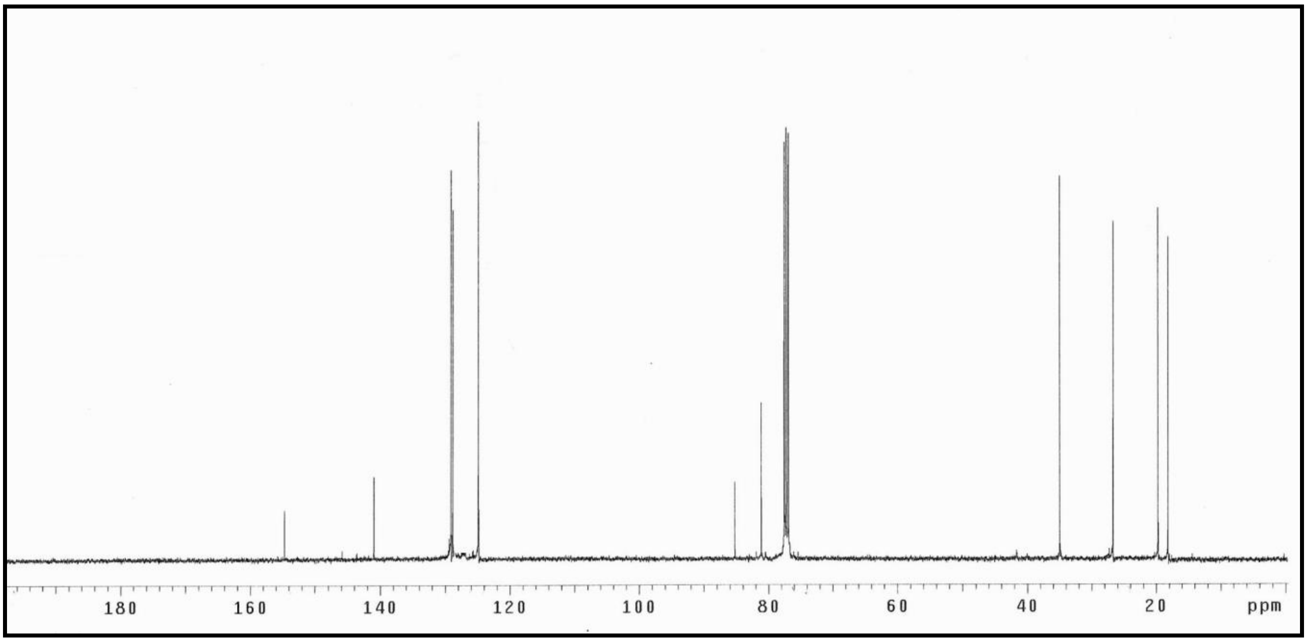

^{13}C NMR spectrum of 3a-phenylhexahydrobenzo[*d*][1,3]dioxol-2-one (**8j**)

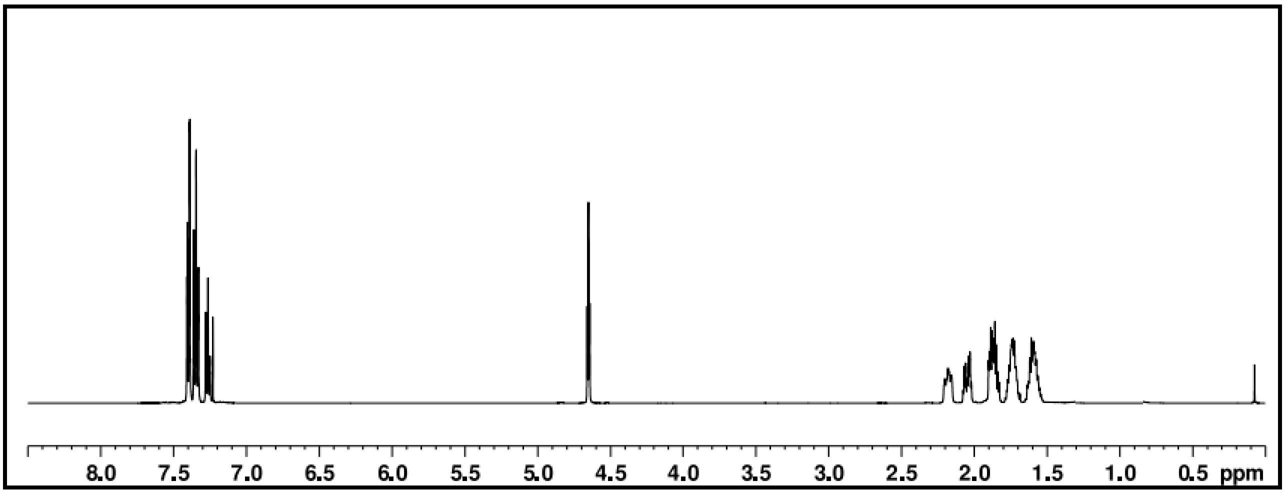

^{1}H NMR spectrum of 3a-phenylhexahydrobenzo[*d*]oxazol-2(3*H*)-one (**9j**)

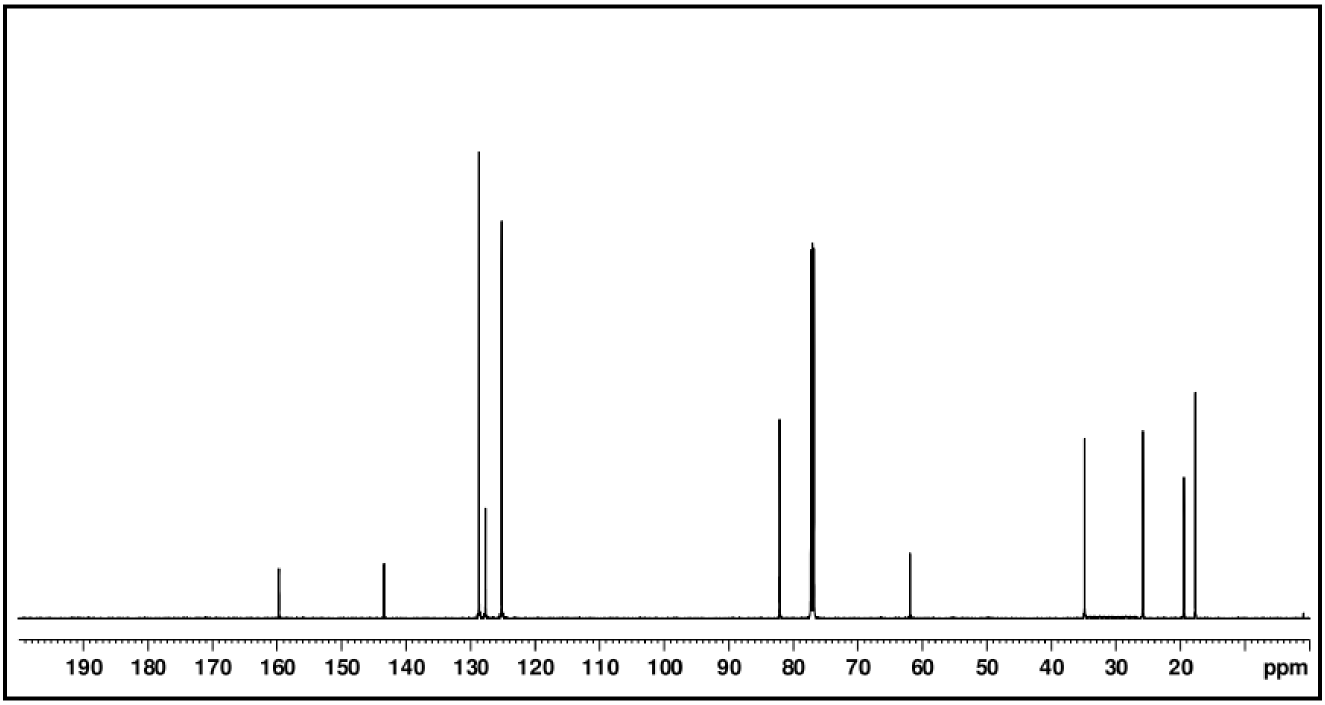

^{13}C NMR spectrum of 3a-phenylhexahydrobenzo[*d*]oxazol-2(3*H*)-one (**9j**)

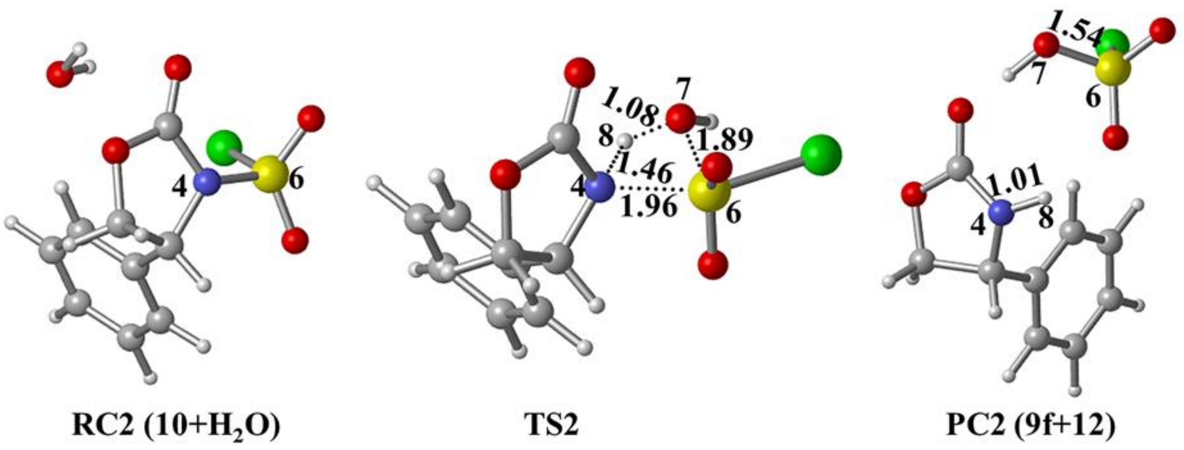

Figure S1. Optimized geometries for the stationary points of path 1a at PCM(DCM)/M06-2X/6-31+G(d,p)//M06-2X/6-31+G(d,p) level. Distances are given in Å.

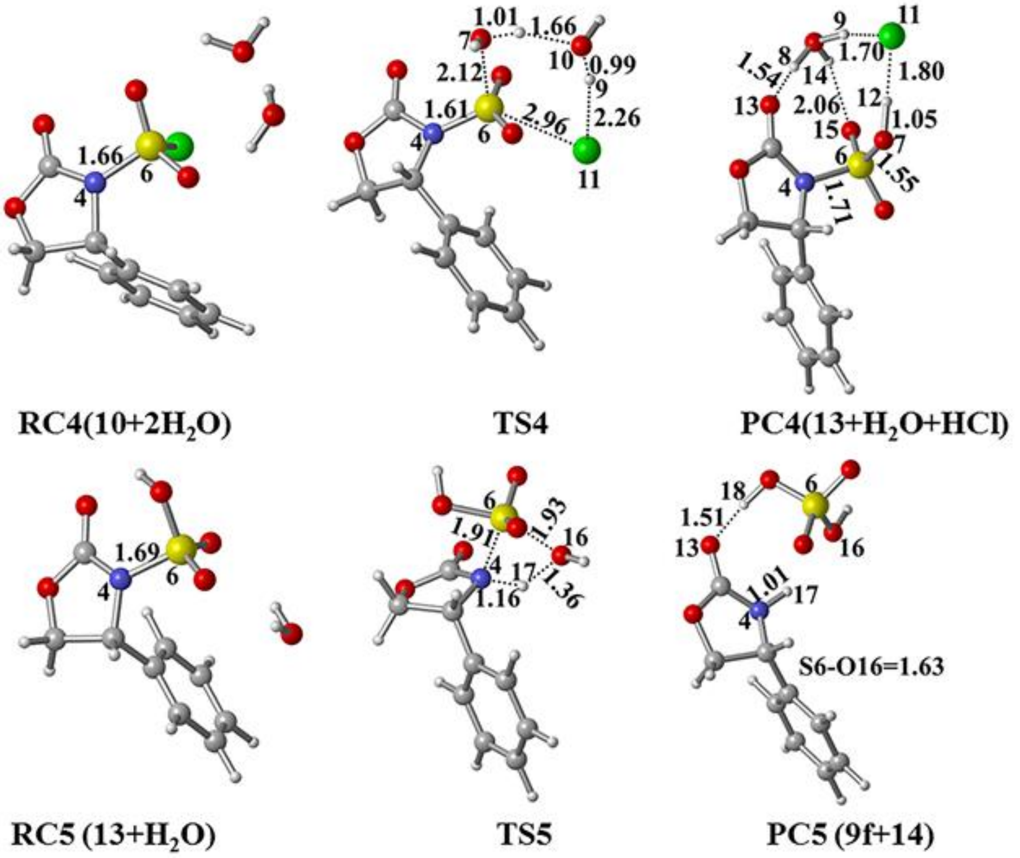

Figure S2. Optimized geometries for the stationary points of path 2 at PCM(DCM)/M06-2X/6-31+G(d,p)//M06-2X/6-31+G(d,p) level. Distances are given in Å.

Absolute energies of structures

Energies are given in terms of ZPE-corrected total energy (Eel+ZPE), enthalpy (Eel+H) and Gibbs free-energy (Eel+G) as extracted from Gaussian [10] output of each structure.

Table S1. Absolute energies of optimized structures for the formation of **10** and **11** in gas phase **(**M06-2X/6-31+G(d,p))

Compound No	*Eel[a]+ZPE[b] (au)*	*Eel+H[c] (au)*	*Eel+G[d] (au)*	*Imaginary Frequency (i)*
RC1(7f+CSI)	-1561.194746	-1561.178848	-1561.241301	–
TS1	-1561.153888	-1561.138832	-1561.197491	-100.6
10	-1561.259136	-1561.244428	-1561.302205	–
RC(7f+CSI)	-1561.194356	-1561.177605	-1561.242869	–
TS1'	-1561.124586	-1561.109231	-1561.169734	-638.32
11	-1561.259812	-1561.244978	-1561.303961	–

[a] Eel = Total electronic energy. [b] ZPE = Zero point energy correction. [c] H = Enthalpy correction. [d] G = Gibbs free energy correction.

Table S2. Single point energies for the formation of **10** and **11** at PCM(DCM)/M06-2X/6-31+G(d,p)//M06-2X/6-31+G(d,p) level.

Compound No	*Eel[a] (au)*	*Eel+G[b] (au)*
RC1(7f+CSI)	-1561.369067	-1561.24944
TS1	-1561.361003	-1561.236926
10	-1561.443435	-1561.31507
RC(7f+CSI)	-1561.368896	-1561.250916
TS1'	-1561.315231	-1561.194255
11	-1561.444182	-1561.317096

Table S3. Absolute energies of optimized structures for path 1a, path 1b and path 2 in gas phase (M06-2X/6-31+G(d,p))

Compound No	*Eel[a]+ZPE[b] (au)*	*Eel+H[c] (au)*	*Eel+G[d] (au)*	*Imaginary Frequency (i)*
RC2(10+H_2O)	-1637.645459	-1637.62761	-1637.691332	–
TS2	-1637.547131	-1637.530831	-1637.592093	-723.3
PC2(9f+12)	-1637.671332	-1637.655843	-1637.715451	–
RC3(10+2H_2O)	-1714.024727	-1714.003944	-1714.074618	–
TS3	-1713.958869	-1713.941923	-1714.004302	-527.9
PC3(9f+12+H_2O)	-1714.04547	-1714.025749	-1714.095205	–
RC4(10+2H_2O)	-1714.024112	-1714.002993	-1714.074864	–
TS4	-1713.935637	-1713.916326	-1713.983151	-230.1
13+H_2O+HCl	-1714.024503	-1714.003043	-1714.102387	–
RC5(13+H_2O)	-1253.279008	-1253.261331	-1253.323976	–
TS5	-1253.182873	-1253.166892	-1253.226232	-966.5
PC5(9f+14)	-1253.296965	-1253.28041	-1253.343945	–

Table S4. Single point energies for path 1a, path 1b and path 2 at PCM(DCM)/M06-2X/6-31+G(d,p)//M06-2X/6-31+G(d,p) level.

Compound No	***Eel[a] (au)***	***Eel+G[b] (au)***
RC2(10+H_2O)	-1637.855513	-1637.705082
TS2	-1637.758664	-1637.61003
PC2(9f+12)	-1637.881053	-1637.727922
RC3(10+2H_2O)	-1714.261539	-1714.089819
TS3	-1714.207323	-1714.032324
PC3(9f+12+H_2O)	-1714.286479	-1714.11308
RC4(10+2H_2O)	-1714.26671	-1714.096141
TS4	-1714.195502	-1714.021157
13+H_2O+HCl	-1714.262539	-1714.126365
RC5(13+H_2O)	-1253.504294	-1253.339321
TS5	-1253.407798	-1253.243687
PC5(9f+14)	-1253.523423	-1253.35909

Table S5. Absolute energies of optimized structures for the formation of **8f** in gas phase **(**M06-2X/6-31+G(d,p))

Compound No	***Eel[a]+ZPE[b] (au)***	***Eel+H[c] (au)***	***Eel+G[d] (au)***	***Imaginary Frequency (i)***
RC6(7f+CSI)	-1561.191475	-1561.174617	-1561.240605	–
TS6	-1561.150784	-1561.135859	-1561.194273	-118.2
16	-1561.239888	-1561.225413	-1561.282908	–
RC7(16+H_2O)	-1637.627309	-1637.609558	-1637.673558	–
TS7	-1637.566139	-1637.55039	-1637.60951	-1251.3
17	-1637.633906	-1637.617977	-1637.676911	–
RC8(17+H_2O)	-1637.632267	-1637.616177	-1637.676000	–
TS8	-1637.591580	-1637.575304	-1637.636346	-199.9
PC8(8f+18)	-1637.655022	-1637.639003	-1637.699032	–

Table S6. Single point energies for the formation of **8f** at PCM(DCM)/M06-2X/6-31+G(d,p)//M06-2X/6-31+G(d,p) level.

Compound No	***Eel[a] (au)***	***Eel+G[b] (au)***
RC6(7f+CSI)	-1561.366947	-1561.249535
TS6	-1561.362049	-1561.237508
16	-1561.426792	-1561.29803
RC7(16+H_2O)	-1637.839263	-1637.688891
TS7	-1637.777903	-1637.627503
17	-1637.845407	-1637.689378
RC8(17+H_2O)	-1637.843896	-1637.688757
TS8	-1637.809078	-1637.657218
PC8(8f+18)	-1637.866644	-1637.712687

Cartesian coordinates for the optimized structures given in paths

Structure No: ***RC1 (7f+CSI) (M06-2X/6-31+G(d,p))***

	X	Y	Z
6	-0.798109	0.522489	1.479827
6	-1.373568	-0.051007	0.249713
6	1.574494	1.422758	-0.782489
1	0.196886	0.213123	1.796450
1	-0.756765	-0.766881	-0.294025
8	-0.839363	1.268893	0.268905
8	1.463753	2.534085	-1.056486
7	1.637964	0.216568	-0.502202
16	3.075441	-0.608660	-0.437455
8	4.067541	0.048924	-1.247780
8	2.757131	-2.000755	-0.563971
17	3.596030	-0.288139	1.523004
6	-2.845837	-0.178208	0.062732
6	-3.389973	-1.412364	-0.296976
6	-3.689281	0.917297	0.260336
6	-4.767974	-1.556567	-0.442116
1	-2.732476	-2.261921	-0.464759
6	-5.066157	0.772585	0.110871
1	-3.255753	1.881084	0.512137
6	-5.608852	-0.464159	-0.237018
1	-5.183425	-2.519758	-0.721611
1	-5.717461	1.628253	0.260341
1	-6.682326	-0.574233	-0.354675
1	-1.474970	0.875150	2.255146

Structure No: ***TS1 (M06-2X/6-31+G(d,p))***

	X	Y	Z
6	-0.746216	-1.451023	-1.209600
6	-1.386374	-0.139017	-0.947315
6	0.950632	-1.049606	0.533417
1	0.077884	-1.374430	-1.918808
1	-0.704333	0.708499	-1.020047
8	-0.291363	-1.660322	0.126473
8	1.399860	-1.470137	1.563410
7	1.318437	-0.118350	-0.361517
16	2.575999	0.825516	-0.027956
8	2.550097	1.377228	1.310875
8	2.767044	1.703036	-1.164589
17	4.213499	-0.480106	-0.061291
6	-2.677897	0.075044	-0.442037
6	-3.038692	1.405546	-0.101599
6	-3.614286	-0.977427	-0.258861
6	-4.303987	1.679538	0.384917

1	-2.305498	2.197378	-0.226432
6	-4.871457	-0.694346	0.234825
1	-3.332656	-1.999469	-0.487731
6	-5.212226	0.629474	0.551292
1	-4.585905	2.693091	0.646088
1	-5.593109	-1.489060	0.385878
1	-6.203498	0.840681	0.941919
1	-1.429497	-2.262251	-1.460595

Structure No: ***10 (M06-2X/6-31+G(d,p))***

	X	Y	Z
6	0.375729	2.265702	-1.079604
6	0.420374	0.720367	-1.066649
6	-1.150129	1.614211	0.492966
1	-0.149068	2.645141	-1.960790
1	0.298508	0.316744	-2.074188
8	-0.372692	2.636244	0.085433
8	-1.979594	1.665385	1.348744
7	-0.803928	0.492707	-0.286480
16	-1.343430	-1.045719	0.061868
8	-1.254388	-1.319914	1.468490
8	-0.738958	-1.885540	-0.940627
17	-3.328576	-0.865909	-0.387980
6	1.666092	0.153059	-0.422441
6	2.690352	-0.327465	-1.238842
6	1.831520	0.153804	0.964804
6	3.876476	-0.794679	-0.677789
1	2.554219	-0.349979	-2.317404
6	3.016477	-0.317496	1.524972
1	1.034921	0.501092	1.618417
6	4.040857	-0.789153	0.705985
1	4.664663	-1.173962	-1.320174
1	3.134701	-0.322289	2.603573
1	4.960789	-1.161268	1.145366
1	1.368012	2.708037	-1.000391

Structure No: ***RC (7f+CSI) (M06-2X/6-31+G(d,p))***

	X	Y	Z
6	1.438491	0.000450	0.382318
6	0.971381	0.812900	1.520024
6	-1.523119	1.324335	-0.796683
1	0.771168	-0.789788	0.037167
1	1.714710	1.286689	2.157790
1	0.001298	0.596796	1.964320
8	0.924597	1.314257	0.188709
6	2.889546	-0.196491	0.107086
6	3.400190	-1.488341	-0.033865
6	3.748266	0.900254	0.004633
6	4.760934	-1.683995	-0.260227
1	2.731003	-2.342651	0.033231

6	5.107362	0.703181	-0.225529
1	3.338057	1.902460	0.089147
6	5.617357	-0.588480	-0.355534
1	5.151283	-2.691203	-0.367486
1	5.769562	1.559406	-0.308900
1	6.676732	-0.740633	-0.536403
8	-1.325215	2.225761	-1.482797
7	-1.677045	0.329367	-0.073218
16	-3.169430	-0.171235	0.450579
8	-4.108650	0.919234	0.394546
8	-2.945068	-0.947767	1.635540
17	-3.678317	-1.478920	-1.041584

Structure No: ***TS1' (M06-2X/6-31+G(d,p))***

	X	Y	Z
6	-1.181822	0.244875	0.215947
6	-0.636570	-0.066117	1.537576
6	0.849790	-0.956046	-0.534661
1	-0.703392	1.095990	-0.272530
1	-1.096992	-0.907508	2.048672
1	0.140326	0.495802	2.037599
8	-0.572733	-1.009155	-0.172141
6	-2.673399	0.210146	0.055054
6	-3.366718	1.415375	-0.068211
6	-3.362757	-1.003851	0.066238
6	-4.755242	1.407249	-0.172619
1	-2.823295	2.356489	-0.093887
6	-4.750758	-1.005492	-0.046705
1	-2.812895	-1.937675	0.132000
6	-5.446859	0.197090	-0.161226
1	-5.293476	2.343785	-0.274249
1	-5.288303	-1.947990	-0.051942
1	-6.528300	0.190510	-0.250887
8	1.158763	-1.456840	-1.572251
7	1.456667	-0.325998	0.476694
16	3.053492	-0.050553	0.449462
8	3.826110	-1.199915	0.049035
8	3.359366	0.679993	1.658176
17	3.242807	1.331140	-1.109056

Structure No: ***11 (M06-2X/6-31+G(d,p))***

	X	Y	Z
6	0.957091	-0.182730	-0.617830
6	0.034819	-0.567907	0.555749
6	-0.778568	1.330080	-0.634340
1	0.785012	-0.851093	-1.468993
1	0.495973	-0.373007	1.527090
1	-0.283050	-1.611428	0.508837
8	0.499544	1.126663	-1.008001
6	2.417226	-0.152884	-0.266534
6	3.259809	-1.165786	-0.721165

6	2.926747	0.860523	0.549212
6	4.605399	-1.175404	-0.355198
1	2.867098	-1.946267	-1.368138
6	4.271485	0.856451	0.905022
1	2.273801	1.661693	0.885496
6	5.111504	-0.164288	0.456861
1	5.256823	-1.966422	-0.712259
1	4.666317	1.650546	1.530552
1	6.160128	-0.165844	0.736775
8	-1.491302	2.190349	-1.042608
7	-1.090689	0.348948	0.348740
16	-2.639675	-0.126238	0.679388
8	-3.510374	1.008883	0.705277
8	-2.535671	-1.087584	1.746088
17	-3.110772	-1.209166	-1.022792

Structure No: ***RC2 (10+H_2O) (M06-2X/6-31+G(d,p))***

	X	Y	Z
6	-0.229395	1.660957	-2.106377
6	0.110273	0.235196	-1.630581
6	-1.640787	1.232389	-0.358845
1	0.652158	2.296847	-2.178833
1	-0.778878	1.652040	-3.050852
1	0.102303	-0.471514	-2.462380
8	-1.088030	2.208731	-1.093262
16	-1.391469	-1.433851	-0.043277
8	-2.789532	-1.539772	0.254211
7	-1.102572	0.009477	-0.808709
8	-2.436838	1.398091	0.519222
1	-1.193022	2.786282	1.708530
8	-0.286914	2.648922	2.013925
1	-0.348960	1.914823	2.634664
8	-0.660140	-2.415890	-0.802365
17	-0.419815	-1.230558	1.757660
6	1.397372	0.112124	-0.846017
6	2.217887	-0.998913	-1.039568
6	1.746491	1.080334	0.100854
6	3.390634	-1.140768	-0.299411
1	1.926047	-1.764449	-1.753923
6	2.916431	0.934542	0.839941
1	1.097997	1.931047	0.301491
6	3.740633	-0.173558	0.639801
1	4.024070	-2.008522	-0.453251
1	3.178183	1.687332	1.577067
1	4.652871	-0.283584	1.218064

Structure No: ***TS2 (M06-2X/6-31+G(d,p))***

	X	Y	Z
6	1.007142	2.021434	-1.355524
6	0.816134	0.534068	-0.994339

6	-0.349353	1.958244	0.479078
1	2.056529	2.299083	-1.462170
1	0.452214	2.298097	-2.255828
1	0.446749	-0.031432	-1.850509
8	0.462721	2.748714	-0.250820
16	-1.927947	-0.316124	-0.280721
8	-2.716173	0.869457	-0.518394
7	-0.235815	0.610836	0.037414
8	-1.020582	2.332244	1.394777
1	-0.412789	-0.251756	1.207207
8	-1.369825	-0.674169	1.488510
1	-1.492090	-1.611493	1.725514
8	-1.339386	-1.251149	-1.220536
17	-3.590431	-1.606022	0.324688
6	2.046214	-0.148138	-0.436911
6	2.392553	-1.428308	-0.868898
6	2.818848	0.478042	0.547504
6	3.503781	-2.076867	-0.328477
1	1.787629	-1.919283	-1.627360
6	3.925407	-0.169092	1.088369
1	2.554862	1.476449	0.892677
6	4.270140	-1.448723	0.649719
1	3.768356	-3.071690	-0.672741
1	4.519713	0.323851	1.851200
1	5.134794	-1.952056	1.070537

Structure No: ***PC2 (9f+12) (M06-2X/6-31+G(d,p))***

	X	Y	Z
6	2.297702	2.413899	0.264136
6	1.943435	1.332819	-0.783303
6	0.078736	2.031120	0.381231
1	3.116527	2.123757	0.921535
1	2.498766	3.382245	-0.201759
1	2.353452	1.608586	-1.759300
8	1.107364	2.545705	1.059439
16	-2.821242	-0.431823	-0.510358
8	-4.016935	-1.002742	-1.056715
7	0.496353	1.484337	-0.778496
8	-1.067927	2.104186	0.824851
1	-0.127670	0.847324	-1.269455
8	-3.093328	0.991763	0.006697
1	-2.199495	1.485281	0.330043
8	-1.573290	-0.463655	-1.257602
17	-2.401704	-1.446860	1.237359
6	2.417094	-0.059267	-0.405316
6	3.765885	-0.376255	-0.595507
6	1.560514	-1.015077	0.139993
6	4.256085	-1.625747	-0.230818
1	4.437184	0.358646	-1.036242
6	2.052064	-2.269320	0.503038
1	0.503103	-0.804028	0.269770
6	3.397117	-2.576810	0.322006
1	5.304463	-1.860720	-0.385136

1	1.373373	-3.005652	0.921528
1	3.775806	-3.554250	0.603000

Structure No: ***RC3 (10+2*** $\pmb{H_2O}$***) (M06-2X/6-31+G(d,p))***

	X	Y	Z
6	-0.148129	2.358322	-1.045614
6	0.175299	0.865674	-1.150016
6	-0.876138	1.408377	0.900881
1	0.689124	3.004432	-1.305418
1	-1.032854	2.626347	-1.629085
1	-0.166064	0.430543	-2.091083
8	-0.448676	2.556301	0.348727
16	-1.730366	-0.891451	-0.194610
8	-1.955043	-1.505192	1.081461
7	-0.670138	0.369969	-0.037198
8	-1.315917	1.296280	2.007636
1	0.893516	-2.053940	1.256147
8	0.743045	-2.653174	0.503175
8	-1.368080	-1.592749	-1.394876
17	-3.490037	0.124960	-0.640004
6	1.613584	0.487281	-0.860468
6	2.070383	-0.767020	-1.264148
6	2.446486	1.314640	-0.096547
6	3.348671	-1.192667	-0.911099
1	1.407001	-1.433201	-1.808094
6	3.726531	0.887498	0.252495
1	2.104751	2.289941	0.241600
6	4.177784	-0.368112	-0.154131
1	3.683421	-2.180202	-1.209631
1	4.367449	1.533974	0.843767
1	5.170885	-0.704038	0.126449
1	0.107198	-3.304605	0.816831
8	1.031945	-0.653873	2.479928
1	1.596808	0.031109	2.100867
1	0.181195	-0.235944	2.675349

Structure No: ***TS3 (M06-2X/6-31+G(d,p))***

	X	Y	Z
6	-0.060067	2.344481	-0.474309
6	0.291738	0.861801	-0.701873
6	-1.087629	1.206764	1.233614
1	0.818500	2.988192	-0.427253
1	-0.769084	2.692705	-1.224183
1	-0.115657	0.524686	-1.656615
8	-0.706004	2.403244	0.806987
16	-1.642104	-1.099734	-0.145645
8	-2.979679	-1.120476	0.400205
7	-0.465216	0.181453	0.397695
8	-1.732686	0.954358	2.201930
1	0.388400	-1.932951	1.632296
8	-0.996751	-2.180666	1.078624

8	-1.027994	-1.729141	-1.290719
17	-2.551665	0.770078	-1.608147
6	1.752656	0.483677	-0.583179
6	2.208830	-0.630394	-1.295937
6	2.613794	1.125982	0.312120
6	3.509220	-1.099459	-1.110758
1	1.529984	-1.138434	-1.976660
6	3.914721	0.658320	0.495981
1	2.268748	1.981651	0.888204
6	4.362476	-0.457815	-0.211530
1	3.854786	-1.963788	-1.668951
1	4.574963	1.161423	1.195123
1	5.374433	-0.822522	-0.067324
1	-1.722799	-2.312300	1.709439
8	1.098541	-1.207600	1.796703
1	1.933857	-1.398930	1.338129
1	0.494490	-0.463393	1.208151

Structure No: ***PC3 (9f+12+H_2O) (M06-2X/6-31+G(d,p))***

	X	Y	Z
6	1.628801	2.130400	-1.326401
6	1.018560	0.921455	-0.592372
6	0.319357	2.922963	0.358625
1	2.685129	2.255740	-1.062400
1	1.516036	2.080611	-2.409328
1	0.151805	0.538795	-1.149036
8	0.889240	3.249658	-0.835829
16	-2.587216	-1.146905	-0.254031
8	-3.804178	-1.577569	-0.873564
7	0.601518	1.598231	0.625887
8	-0.305751	3.695076	1.033690
1	0.394575	-1.459037	0.919188
8	-2.565683	-1.651126	1.226883
8	-1.288399	-1.383260	-0.852198
17	-2.709451	0.885391	0.007387
6	1.984765	-0.213361	-0.348817
6	1.874787	-1.397975	-1.082168
6	2.983787	-0.100363	0.625668
6	2.756797	-2.456388	-0.848616
1	1.086099	-1.497286	-1.823626
6	3.860947	-1.155673	0.857963
1	3.051301	0.813782	1.209521
6	3.748047	-2.337795	0.122057
1	2.660932	-3.373193	-1.421716
1	4.632395	-1.058762	1.615711
1	4.430366	-3.161252	0.307624
8	-0.084499	-1.215360	1.730305
1	0.490574	-1.419991	2.476943
1	-0.010650	1.126211	1.279641
1	-1.623993	-1.553311	1.585267

Structure No: ***RC4 (10+2H_2O) (M06-2X/6-31+G(d,p))***

	X	Y	Z
6	-1.988521	1.943252	1.488851
6	-1.157976	0.659023	1.325844
6	-0.543547	2.345288	-0.240589
1	-3.060194	1.748589	1.520056
1	-1.675702	2.515717	2.365530
1	-0.745171	0.327538	2.280147
8	-1.711756	2.722557	0.314730
16	1.262385	0.364246	0.061204
8	2.328878	1.278856	-0.247622
8	1.378116	-0.674224	1.055897
17	0.701756	-0.524676	-1.685858
7	-0.067395	1.243233	0.512137
8	-0.031917	2.846649	-1.192408
8	4.160305	-1.716636	1.724697
1	3.220415	-1.575015	1.877188
8	4.116934	-0.755807	-1.015263
1	4.757088	-0.977079	-1.698313
1	4.028446	0.205827	-1.002914
1	4.281970	-1.478219	0.793901
6	-1.865985	-0.483781	0.631291
6	-1.612123	-1.793571	1.038005
6	-2.726443	-0.248829	-0.446714
6	-2.212814	-2.863397	0.376507
1	-0.924896	-1.976459	1.859521
6	-3.325431	-1.317517	-1.106346
1	-2.926182	0.766849	-0.781851
6	-3.067862	-2.626083	-0.696589
1	-2.006390	-3.879429	0.696828
1	-3.990966	-1.128823	-1.942559
1	-3.534941	-3.458039	-1.213967

Structure No: ***TS4 (M06-2X/6-31+G(d,p))***

	X	Y	Z
6	-1.267644	2.751753	-0.339769
6	-0.950427	1.501327	0.497927
6	0.986114	2.456622	-0.591936
1	-1.744609	2.470070	-1.282461
1	-1.878529	3.474354	0.198923
1	-0.867522	1.756187	1.560664
8	0.000481	3.359062	-0.627003
16	1.250469	-0.112010	0.176955
8	2.318927	-0.161672	-0.754381
8	0.504414	-0.940673	1.067032
17	0.788094	-2.633546	-1.314520
7	0.412770	1.249503	-0.032137
8	2.115629	2.622572	-0.908298
8	2.702771	0.177739	1.696558
1	2.316023	0.157207	2.584570
8	3.041867	-2.324792	0.973058

1	2.321994	-2.601922	0.348683
1	3.855750	-2.624226	0.552144
1	3.048108	-0.761254	1.524921
6	-1.954081	0.394996	0.299043
6	-2.945123	0.221415	1.265751
6	-1.932032	-0.419094	-0.834488
6	-3.921359	-0.757221	1.094075
1	-2.949379	0.842282	2.158741
6	-2.888611	-1.417745	-0.986653
1	-1.144418	-0.332839	-1.579553
6	-3.889315	-1.580204	-0.030047
1	-4.690579	-0.889783	1.847804
1	-2.826259	-2.082086	-1.841506
1	-4.632922	-2.361044	-0.151551

Structure No: ***13 (M06-2X/6-31+G(d,p))***

	X	Y	Z
6	-0.078237	2.401228	-0.191584
6	0.165129	0.949901	-0.660306
6	-1.966906	1.245022	0.315756
1	0.490986	2.614200	0.717374
1	0.141461	3.141378	-0.959870
1	0.040753	0.855000	-1.745768
8	-1.478851	2.478360	0.120802
16	-1.344714	-1.325735	-0.101434
8	-1.515428	-1.835442	1.232086
8	-0.399476	-1.869105	-1.035378
7	-0.968808	0.321596	0.027071
8	-3.096218	0.992672	0.654639
8	-2.755159	-1.281123	-0.836589
1	-3.396407	-0.825983	-0.250102
6	1.514093	0.414983	-0.248591
6	2.524963	0.300311	-1.200926
6	1.771689	0.074109	1.081428
6	3.793202	-0.141016	-0.827644
1	2.316391	0.540799	-2.240413
6	3.035621	-0.375736	1.451396
1	0.976857	0.137838	1.821077
6	4.049304	-0.479718	0.498708
1	4.574252	-0.233691	-1.575344
1	3.227609	-0.652477	2.483075
1	5.033079	-0.833885	0.789516

Structure No: ***RC5 (13+H_2O) (M06-2X/6-31+G(d,p))***

	X	Y	Z
6	0.678470	-2.522285	-0.870992
6	0.157845	-1.075253	-1.062515
6	2.013775	-1.199027	0.410163
1	-0.115571	-3.213796	-0.591940
1	1.212734	-2.880912	-1.754076
1	0.117255	-0.818041	-2.123554

8	1.617344	-2.460740	0.218203
16	1.262150	1.291765	-0.135101
8	0.693760	1.543020	1.167917
8	0.704645	1.899966	-1.314909
7	1.287355	-0.368866	-0.437395
8	2.873386	-0.844822	1.180584
8	2.815597	1.580694	-0.104651
1	3.210973	1.048539	0.623022
8	-1.972464	2.612927	0.119657
1	-1.428500	2.307087	0.855604
1	-1.362208	2.649136	-0.626540
6	-1.178803	-0.798197	-0.413885
6	-2.280851	-0.506671	-1.215882
6	-1.330252	-0.861993	0.974562
6	-3.530317	-0.286882	-0.639872
1	-2.157994	-0.432597	-2.293805
6	-2.577020	-0.638441	1.548885
1	-0.474701	-1.066847	1.614885
6	-3.678205	-0.351800	0.741628
1	-4.379581	-0.041054	-1.268504
1	-2.687573	-0.678589	2.627729
1	-4.646705	-0.164161	1.193608

Structure No: ***TS5 (M06-2X/6-31+G(d,p))***

	X	Y	Z
6	0.101777	1.708283	-1.433272
6	0.355352	0.289690	-0.895374
6	-0.931682	1.619273	0.609177
1	1.017611	2.208918	-1.747392
1	-0.640993	1.705157	-2.231843
1	0.029135	-0.465360	-1.614646
8	-0.438888	2.437540	-0.323249
16	-2.068304	-0.887280	-0.021255
8	-3.267586	-0.725536	0.778458
8	-1.647045	-1.998350	-0.865341
7	-0.563706	0.247820	0.275775
8	-1.547434	1.930458	1.575912
8	-2.444039	0.242583	-1.234162
1	-3.385487	0.446347	-1.135423
8	-1.084515	-1.572142	1.496047
1	-0.335016	-0.499704	1.127004
1	-0.823454	-2.484052	1.308137
6	1.766638	0.002218	-0.438440
6	2.311778	-1.264025	-0.655694
6	2.512417	0.968845	0.243255
6	3.596514	-1.560880	-0.203350
1	1.726711	-2.017094	-1.178335
6	3.794211	0.670793	0.696320
1	2.093060	1.956913	0.423756
6	4.338012	-0.594221	0.471955
1	4.016741	-2.545927	-0.379104
1	4.368000	1.425055	1.224945
1	5.338459	-0.824788	0.823792

Structure No: ***PC5 (9f+14) (M06-2X/6-31+G(d,p))***

	X	Y	Z
6	1.174365	2.052064	-0.936788
6	1.057776	0.554249	-0.599895
6	-0.693955	1.748379	0.292789
1	1.953784	2.531715	-0.336251
1	1.331499	2.252176	-1.995786
1	0.608586	0.009323	-1.441219
8	-0.098608	2.598415	-0.554233
16	-3.018254	-0.915701	-0.152190
8	-4.052538	-1.882973	-0.392920
8	-2.116313	-0.462204	-1.183892
7	0.088265	0.654779	0.486016
8	-1.781792	1.993953	0.800474
8	-3.647427	0.293253	0.606236
1	-2.936656	1.025398	0.732189
8	-2.006540	-1.522302	0.973600
1	-0.357652	-0.179497	0.853589
1	-2.516333	-2.004376	1.644624
6	2.367855	-0.085710	-0.214807
6	3.033727	-0.894402	-1.136134
6	2.945459	0.150418	1.035972
6	4.268600	-1.457150	-0.817991
1	2.579627	-1.091276	-2.104246
6	4.175986	-0.416201	1.356378
1	2.419778	0.768372	1.759230
6	4.841024	-1.219178	0.429392
1	4.777146	-2.087161	-1.540791
1	4.615989	-0.234602	2.331995
1	5.799170	-1.662254	0.681982

Structure No: ***RC6 (7f+CSI) (M06-2X/6-31+G(d,p))***

	X	Y	Z
6	-1.620993	1.963123	-1.090884
6	-1.966637	1.128917	0.075091
6	1.497227	1.116206	0.226556
1	-1.042956	2.873962	-0.944971
1	-1.618458	1.471838	1.050020
8	-0.921954	0.755165	-0.815713
16	3.166980	-0.815755	0.278629
8	3.093381	-2.098942	-0.352960
8	3.249576	-0.646451	1.706445
17	4.776242	0.165637	-0.531294
1	-2.268282	1.926543	-1.964518
6	-3.181237	0.266130	0.083645
6	-3.406850	-0.654132	-0.942328
6	-4.110579	0.389575	1.118089
6	-4.560017	-1.435039	-0.936341
1	-2.663936	-0.764583	-1.726975

6	-5.265673	-0.389282	1.120039
1	-3.929778	1.096544	1.924148
6	-5.492796	-1.302708	0.091943
1	-4.727059	-2.154713	-1.731663
1	-5.984729	-0.286980	1.926693
1	-6.389424	-1.914287	0.095662
8	1.075562	2.091751	0.678761
7	1.918519	0.097002	-0.323083

Structure No: ***TS6 (M06-2X/6-31+G(d,p))***

	X	Y	Z
6	1.045920	2.092945	0.117007
6	1.763376	1.101620	-0.726534
6	-0.888221	0.679200	0.094663
1	0.439793	2.775323	-0.479322
1	1.256343	0.843205	-1.656176
8	0.252416	1.153043	0.828300
16	-2.845938	-0.864243	0.274267
8	-3.582333	-1.500762	1.340604
8	-2.461383	-1.629104	-0.896363
17	-4.140614	0.634275	-0.444510
1	1.665494	2.623674	0.838659
6	2.888176	0.355554	-0.349992
6	3.564667	0.568255	0.881971
6	3.349882	-0.649188	-1.242203
6	4.674025	-0.190883	1.192022
1	3.190987	1.304737	1.584782
6	4.470162	-1.395759	-0.926988
1	2.809374	-0.820333	-2.168941
6	5.125155	-1.163775	0.287013
1	5.190938	-0.045871	2.133751
1	4.830439	-2.160958	-1.604891
1	5.998813	-1.756897	0.540624
8	-0.969444	1.001465	-1.079483
7	-1.618518	-0.043230	0.924813

Structure No: 16 (M06-2X/6-31+G(d,p))

	X	Y	Z
6	-1.104860	2.425217	-0.367748
6	-1.191637	1.304232	0.680614
6	0.665180	1.061799	-0.574577
1	-0.804399	3.384880	0.060397
1	-1.310422	1.698872	1.691952
8	-0.048302	1.966754	-1.227710
16	2.463768	-0.695221	-0.368665
8	3.633030	-1.019323	-1.137511
8	1.490162	-1.707893	-0.018969
17	3.161502	0.086304	1.427485
1	-2.007717	2.529602	-0.968148
6	-2.222702	0.239732	0.380464
6	-1.855055	-1.077295	0.106934

6	-3.573124	0.601833	0.394585
6	-2.844820	-2.023402	-0.164987
1	-0.808838	-1.369799	0.116214
6	-4.554081	-0.345943	0.123883
1	-3.862798	1.625885	0.624137
6	-4.188821	-1.662903	-0.159243
1	-2.556089	-3.047209	-0.379658
1	-5.600815	-0.059458	0.136709
1	-4.952888	-2.404529	-0.369071
8	0.140190	0.756593	0.606514
7	1.748145	0.598492	-1.082755

Structure No: ***RC7 (16+H_2O) (M06-2X/6-31+G(d,p))***

	X	Y	Z
6	1.191579	1.145638	-0.308652
6	1.060149	2.070167	0.921935
6	-0.686803	0.664031	0.852614
1	1.182411	1.726329	-1.234052
8	-0.046499	0.412524	-0.272422
8	-0.066629	1.524565	1.630141
7	-1.815029	0.152293	1.216518
16	-2.431518	-1.063090	0.324514
8	-1.436450	-2.053186	-0.021527
8	-3.697101	-1.435166	0.893956
17	-2.926233	-0.102576	-1.496486
8	-1.309446	2.822996	-0.746311
1	-1.997156	3.391822	-0.384123
1	-1.775371	2.162670	-1.277911
1	1.925269	2.024172	1.583251
1	0.815699	3.095526	0.645453
6	2.359996	0.191142	-0.262835
6	2.183322	-1.174512	-0.042156
6	3.646385	0.711257	-0.431998
6	3.298806	-2.011095	0.020868
1	1.183183	-1.586603	0.060368
6	4.754460	-0.126872	-0.365729
1	3.783841	1.774223	-0.621878
6	4.581102	-1.492361	-0.136053
1	3.158003	-3.073873	0.189584
1	5.750445	0.283220	-0.498970
1	5.444165	-2.148530	-0.087958

Structure No: ***TS7 (M06-2X/6-31+G(d,p))***

	X	Y	Z
6	1.142330	1.436558	-0.108117
6	0.549296	1.822607	1.246928
6	-1.127517	1.415452	-0.217610
1	1.441165	2.326826	-0.675330
8	-0.019984	0.864582	-0.747575
8	-0.812075	2.129489	0.906345
7	-2.311765	0.652458	-0.239680

16	-2.280046	-0.815990	0.394738
8	-1.253107	-0.878046	1.422695
8	-3.623678	-1.276614	0.632105
17	-1.551543	-2.048674	-1.128448
8	-1.757853	2.442311	-1.236418
1	-1.973234	3.264306	-0.762476
1	-2.591973	1.654151	-1.150693
1	0.554493	0.978264	1.942740
1	1.004086	2.706976	1.692230
6	2.256191	0.431508	-0.063223
6	2.003047	-0.870113	0.381707
6	3.545365	0.801958	-0.443146
6	3.044294	-1.791006	0.440273
1	0.993522	-1.157853	0.667048
6	4.588379	-0.120607	-0.371965
1	3.735215	1.810578	-0.802374
6	4.336622	-1.417748	0.067384
1	2.845891	-2.803702	0.776314
1	5.590554	0.171652	-0.668620
1	5.145126	-2.140527	0.116130

Structure No: ***17 (M06-2X/6-31+G(d,p))***

	X	Y	Z
6	1.067852	1.418514	-0.070462
6	0.437125	1.735436	1.288023
6	-1.180452	1.542099	-0.323577
1	1.374399	2.341600	-0.579227
8	-0.062184	0.866210	-0.764030
8	-0.911991	2.058938	0.940303
7	-2.335820	0.648335	-0.285724
16	-2.254807	-0.852508	0.362376
8	-1.311880	-0.821820	1.452726
8	-3.603752	-1.336619	0.493659
17	-1.397304	-2.033526	-1.101519
8	-1.524875	2.565063	-1.193024
1	-1.818580	3.313980	-0.657023
1	-2.953546	0.722399	-1.089292
1	0.439954	0.862344	1.946310
1	0.887238	2.596929	1.782798
6	2.201514	0.434080	-0.038741
6	1.983507	-0.873508	0.406979
6	3.480747	0.827732	-0.428849
6	3.041816	-1.775061	0.455535
1	0.984647	-1.179495	0.707431
6	4.542857	-0.074028	-0.370082
1	3.647060	1.840912	-0.786338
6	4.322947	-1.376510	0.070060
1	2.867803	-2.790825	0.796356
1	5.536132	0.239508	-0.675093
1	5.146696	-2.082330	0.111576

Structure No: ***RC8 (17+H_2O) (M06-2X/6-31+G(d,p))***

	X	Y	Z
6	1.304566	1.220124	0.112038
6	0.430818	1.035137	1.355965
6	-0.863980	1.365418	-0.511113
1	1.609155	2.270103	0.006957
8	0.364476	0.900027	-0.920018
8	-0.866780	1.420301	0.886000
7	-1.893367	0.475582	-1.035306
16	-2.245578	-0.960326	-0.350529
8	-3.120013	-1.664180	-1.251733
8	-1.031582	-1.520514	0.186543
17	-3.422218	-0.494405	1.289298
8	-1.137897	2.620989	-1.032448
1	-1.491445	3.159980	-0.312409
1	0.404094	-0.012089	1.671357
1	0.709365	1.685201	2.186126
6	2.499733	0.313829	0.037384
6	2.323920	-1.060809	-0.147013
6	3.783530	0.835632	0.189442
6	3.432459	-1.901319	-0.179771
1	1.320015	-1.457507	-0.272974
6	4.892276	-0.009815	0.166174
1	3.919746	1.906608	0.319256
6	4.717100	-1.378512	-0.020896
1	3.294078	-2.967349	-0.330410
1	5.889193	0.402766	0.284447
1	5.579132	-2.037712	-0.046375
1	-2.660972	0.919923	-1.528434

Structure No: ***TS8 (M06-2X/6-31+G(d,p))***

	X	Y	Z
6	1.674487	1.465716	0.374970
6	0.573169	1.371293	1.444451
6	-0.305607	2.146621	-0.429245
1	2.339963	2.315146	0.550374
8	0.887905	1.845753	-0.817053
8	-0.510224	2.131762	0.847923
7	-1.516355	0.244095	-0.867694
16	-2.002300	-0.854464	0.149548
8	-1.483788	-2.189852	-0.121893
8	-1.916488	-0.337781	1.510213
17	-4.084852	-1.059589	-0.179686
8	-1.105760	2.751208	-1.244338
1	-1.980087	2.317973	-1.131406
1	0.197447	0.361210	1.616713
1	0.834647	1.858040	2.381883
6	2.465370	0.218713	0.122442

6	1.826745	-1.004153	-0.109029
6	3.859458	0.287863	0.124700
6	2.586620	-2.146764	-0.335969
1	0.743010	-1.079231	-0.127006
6	4.617763	-0.860009	-0.094477
1	4.354176	1.240328	0.297833
6	3.980195	-2.076759	-0.325980
1	2.082938	-3.090888	-0.515299
1	5.701207	-0.802065	-0.087627
1	4.568286	-2.972544	-0.499346
1	-1.390187	-0.196036	-1.780355

Structure No: ***PC8 (8f+18) (M06-2X/6-31+G(d,p))***

	X	Y	Z
6	1.635477	1.405601	0.272449
6	0.531303	1.122422	1.299755
6	-0.404986	1.887831	-0.601689
1	2.144214	2.350766	0.493291
8	0.860841	1.609937	-0.928065
8	-0.605421	1.773770	0.721775
7	-3.279486	0.198271	-1.178984
16	-2.539281	-0.942753	-0.311282
8	-3.231078	-2.192532	-0.482817
8	-1.114362	-0.798003	-0.514351
17	-2.846851	-0.434113	1.685085
8	-1.262623	2.208272	-1.382034
1	-2.787600	1.099460	-1.219681
1	0.314813	0.053035	1.382922
1	0.725128	1.558105	2.279153
6	2.639136	0.303613	0.091018
6	2.235099	-0.945930	-0.389600
6	3.975459	0.520044	0.424489
6	3.169870	-1.966226	-0.533389
1	1.194834	-1.112299	-0.656986
6	4.908564	-0.507308	0.289530
1	4.291291	1.495731	0.785532
6	4.506003	-1.750245	-0.191728
1	2.854507	-2.932612	-0.913524
1	5.947221	-0.332003	0.551008
1	5.231757	-2.549330	-0.305196
1	-4.289209	0.214851	-1.090636

References

[1] Laserna, V.; Fiorani, G.; Whiteoak, C. J.; Martin, E.; Escudero-Adán, E.; Kleij, A. W. *Angew. Chemie - Int. Ed.* **2014**, *53*, 10416-10419.

[2] Scholz, K.H.; Heine, H. G.; Hartmann, W. *Liebigs Ann. Chem.* **1977**, *11*, 2027-2035.

[3] Steinbauer, J.; Spannenberg, A.; Werner, T. *Green Chem.* **2017**, *19*, 3769-3779.

[4] Hu, N. X.; Aso, Y.; Otsubo, T.; Ogura, F. *J. Org. Chem.* **1989**, *54*, 4398-4404.

[5] Yang, X.; Bumbu, V. D.; Liu, P.; Li, X.; Jiang, H.; Uffman, E. W.; Guo, L.; Zhang, W.; Jiang, X.; Houk, K. N. *J. Am. Chem. Soc.* **2012**, *134*, 17605-17612.

[6] Peña-López, M.; Neumann, H.; Beller, M. *European J. Org. Chem.* **2016**, *2016*, 3721-3727.

[7] Sukhanova, A. A.; Puchkin, I. A.; Vasil'ev, A. A.; Zlotin, S. G. *Tetrahedron Asymmetry* **2017**, 28, 1834-1841.

[8] Miceli, C.; Rintjema, J.; Martin, E.; Escudero-Adán, E. C.; Zonta, C.; Licini, G.; Kleij, A. W. *ACS Catal.* **2017**, 7, 2367-2373.

[9] Lebel, H.; Mamani Laparra, L.; Khalifa, M.; Trudel, C.; Audubert, C.; Szponarski, M.; Dicaire Leduc, C.; Azek, E.; Ernzerhof, M. *Org. Biomol. Chem.* **2017**, 15, 4144-4158.

[10] M. J. Frisch, G. W. Trucks, H. B. Schlegel, G. E. Scuseria, M. A. Robb, J. R. Cheeseman, G. Scalmani, V. Barone, B. Mennucci, G. A. Petersson, H. Nakatsuji, M. Caricato, X. Li, H. P. Hratchian, A. F. Izmaylov, J. Bloino, G. Zheng, J. L. Sonnenberg, M. Hada, M. Ehara, K. Toyota, R. Fukuda, J. Hasegawa, M. Ishida, T. Nakajima, Y. Honda, O. Kitao, H. Nakai, T. Vreven, J. A. Montgomery Jr., J. E. Peralta, F. Ogliaro, M. Bearpark, J. J. Heyd, E. Brothers, K. N. Kudin, V. N. Staroverov, R. Kobayashi, J. Normand, K. Raghavachari, A. Rendell, J. C. Burant, S. S. Iyengar, J. Tomasi, M. Cossi, N. Rega, J. M. Millam, M. Klene, J. E. Knox, J. B. Cross, V. Bakken, C. Adamo, J. Jaramillo, R. Gomperts, R. E. Stratmann, O. Yazyev, A. J. Austin, R. Cammi, C. Pomelli, J. W. Ochterski, R. L. Martin, K. Morokuma, V. G. Zakrzewski, G. A. Voth, P. Salvador, J. J. Dannenberg, S. Dapprich, A. D. Daniels, Ö. Farkas, J. B. Foresman, J. V. Ortiz, J. Cioslowski, D. J. Fox, Gaussian 09, Revision B.01, Gaussian, Inc., Wallingford CT, **2009**.
